# Supplementary material for: Terminator-free template-independent enzymatic DNA synthesis for digital information storage
Source: Nat Commun. 2019 Jun 3;10:2383. doi: 10.1038/s41467-019-10258-1 (PMC6546792; doi:10.1038/s41467-019-10258-1)
Supplement: Supplementary file 1 — Supplementary Information [file 41467_2019_10258_MOESM1_ESM.pdf]

Supplementary Materials for

Terminator-free template-independent enzymatic DNA synthesis for digital information storage

Lee, HH., Kalhor, R., Goela, N., Bolot, J., Church, GM

Correspondence to George Church (gchurch@genetics.med.harvard.edu) or
Henry H. Lee (hhlee@genetics.med.harvard.edu)

Table of Contents

Supplementary Notes 5

1. TdT:apyrase system 5
   - 1.1 TdT to apyrase ratio optimization 5
   - 1.2 Optimizing the reaction conditions for TdT:apyrase 6
     - 1.2.1 Divalent cations 6
     - 1.2.2 Buffer and salt concentrations 9
     - 1.2.3 Additives 9
     - 1.2.4 Polymerase to initiator ratio 11
   - 1.3 Optimizing extension length for each nucleotide transition type 12
   - 1.4 Synthesizing a template sequence using optimized TdT:apyrase 14
   - 1.5 Use of the nucleotide analogue, 5-Bromo-dCTP 14
2. Codec for information storage with enzymatically-synthesized DNA 15
   - 2.1 Encoder and decoder architecture 15
   - 2.2 Addressing of rows/sequences 17
   - 2.3 Reed-Solomon and Fountain Codes (Block ECC) 17
   - 2.4 Error-Correcting Codes per sequence 19
   - 2.5 Modulation and demodulation (Bits ⇔ Nucleotides) 21
     - 2.5.1 Nucleotide transition matrix 22
     - 2.5.2 Synchronization nucleotides 22
     - 2.5.3 Modulation and demodulation (Experimental) 22
     - 2.5.4 Modulation and demodulation (Simulation) 23
     - 2.5.5 Summary of modulation and demodulation 23
   - 2.6 Efficiency rates for DNA storage with a digital codec 24
     - 2.6.1 Efficiency rates for experimental and simulated systems 24
     - 2.6.2 Higher efficiency rates with increased synthesis accuracy 25
   - 2.7 Alignment of DNA strands by scaffolding and consensus 25
     - 2.7.1 Optimal alignment of diverse strands 27
     - 2.7.2 Alignment by consensus 27
     - 2.7.3 Markov chain model 28
     - 2.7.4 Forward-Backward recursions 29
     - 2.7.5 Decoding based on alignment of multiple DNA strands 31
     - 2.7.6 Computational complexity of optimal vs. consensus alignment 31
     - 2.7.7 Alternative alignment algorithms 32
     - 2.7.8 Considerations of increased diversity for consensus 32
3. Diversity analyses of enzymatic synthesis 33
   - 3.1 Distribution of lengths for compressed DNA strands 33
     - 3.1.1 Exact length distribution (Theory) 35
     - 3.1.2 Binomial distribution (Special case) 35
     - 3.1.3 Experimental distribution of length 36
   - 3.2 Diversity of synthesized strands 37
     - 3.2.1 Mathematical diversity 37
     - 3.2.2 Empirical diversity of DNA strands 38
4. Factors impacting scalable DNA storage systems 39
   - 4.1 Four main factors affecting scalability 39

4.1.1 Cost of synthesis 39
4.1.2 Number of template sequences that can by synthesized....39
4.1.3 Number of nucleotides per template sequence 40
4.1.4 Efficiency rate of storage....40
4.2 Soup of DNA: DNA storage modeled as a permutation channel....40
4.3 Clustering of DNA strands....42
4.3.1 Clustering DNA strands based on relative edit distances....42
4.3.2 Alternative methods for clustering DNA strands 43

5. 2D array-format synthesis 43
5.1 Initiator immobilization and surface preparation....43
5.2 Synthesis, processing, and sequencing 44
5.3 Analyses and future improvements....46

6. Cost projections and cycle time estimation.... 47
6.1 Synthesizing DNA with an inkjet microarray printer 47
6.2 Reagent costs....47
6.2.1 Reagent cost per cycle 48
6.2.2 Reagent cost per megabyte 49
6.2.3 Practical considerations 50
6.3 Equipment costs 52

Supplementary Figures 53
Supplementary Figure 1. A single cycle of phosphoramidite chemistry versus this enzymatic synthesis strategy. 53
Supplementary Figure 2. Optimizing and tuning of TdT:apyrase ratio. 54
Supplementary Figure 4. Buffer and additives optimization for TdT:apyrase. 57
Supplementary Figure 5. Optimizing TdT to initiator ratio. 59
Supplementary Figure 6. Optimal nucleotide concentrations based on 3' nucleotide to be extended....60
Supplementary Figure 7. TdT:apyrase activity over multiple cycles. 62
Supplementary Figure 8. Evaluation of 5-Bromo-dCTP as a substitute for natural dCTP....63
Supplementary Figure 9. Extension lengths for perfect strands of H01-H12. 64
Supplementary Figure 10. Raw lengths for all and perfect strands of H01-H12. 65
Supplementary Figure 11. Synthesis error analysis for all strands of H01-H12....66
Supplementary Figure 12. Nanopore sequencing and decoding of H01-H12. 67
Supplementary Figure 13. Synthesis error analyses and diversity of all synthesized strands of E0....69
Supplementary Figure 14. Constraints for valid transitions between nucleotides. 71
Supplementary Figure 15. Placement and modulation of information into template sequences. 72
Supplementary Figure 16. Markov model for the production of DNA strands. 74
Supplementary Figure 17. Reconstruction of a template sequence by MAP estimation. 76
Supplementary Figure 18. Raw lengths for all and perfect strands for E1-E4. 78
Supplementary Figure 19. Synthesis error analysis for all strands and purified strands of E1-E4....79
Supplementary Figure 20. Lengths, diversity, and edit distance for all and purified strands for E1-E4. 81
Supplementary Figure 21. Diversity of compressed synthesized strands for E0. 83
Supplementary Figure 22. Diversity of compressed synthesized strands for E1. 84
Supplementary Figure 23. Diversity of compressed synthesized strands for E2. 85
Supplementary Figure 24. Diversity of compressed synthesized strands for E3. 86
Supplementary Figure 25. Diversity of compressed synthesized strands for E4. 87
Supplementary Figure 26. Decoding curves for E1-E4 template sequences for "Eureka!". 88

Supplementary Figure 27. Estimated capacity in bits per template sequence with increased synthesis accuracy for simulated DNA storage systems....90
Supplementary Figure 28. Waterfall decoding curves for simulated DNA storage systems....92
Supplementary Figure 29. Majority alignment of DNA strands per sequence....94
Supplementary Figure 30. System architecture of codec for storing information in DNA. 95
Supplementary Figure 31. Array-format enzymatic synthesis platform....97
Supplementary Figure 32. Raw lengths for all and perfect raw strands for S01-S03....99
Supplementary Figure 33. Synthesis error analysis for all and purified strands for S01-S03. 100
Supplementary Figure 34. Lengths, diversity, and edit distance for all and purified strands for S01-S03. 102
Supplementary Figure 35. Reagent cost projections for phosphoramidite chemistry and enzymatic synthesis. 104

Supplementary Tables 105

Supplementary Table 1. Oligonucleotides used in this study. 105
Supplementary Table 2. Conversion of "hello world!" to template sequences. 106
Supplementary Table 3. Extension lengths for perfectly synthesized strands of "hello world!". 107
Supplementary Table 4. Final concentration, in micromolar, of nucleoside triphosphates used for synthesizing H01-H12 template sequences....107
Supplementary Table 5. Statistics from simulated real-time data reconstruction by nanopore sequencing....108
Supplementary Table 6. Commercial reagent prices and estimated reaction times for our enzymatic synthesis vs. phosphoramidite chemistry. 109
Supplementary Table 7. Parameters of DNA Storage Systems 111
Supplementary Table 8. Design Specifications of DNA Storage Systems 111
Supplementary Table 9. Modulation and demodulation: Interconversion of bits to nucleotides for "Eureka!" experiment....112

Supplementary References.... 113

Supplementary Notes

1. TdT:apyrase system

1.1 TdT to apyrase ratio optimization

To obtain a ratio of TdT polymerization activity to apyrase degradation activity that would allow for net positive extension of the initiator, we assessed initiator extensions by TdT in presence of a wide range of apyrase concentrations with every dNTP substrate (**Supplementary Fig. 2A**).

Each reaction was carried out in 20µL total volume. All reaction components but the dNTP were assembled in 18µL while the dNTP was prepared in 2µL of water. The 18µL mix was composed such that upon mixing with the 2µL dNTP solution, the following initial composition would be obtained: 200µM dNTP, 1X Enzymatics Green Buffer, 0.05µM f-P5-SBS3 initiator oligo, 1U/µL TdT, and 4, 2, 1, 0.5, or 0.25 milliunits (mU) of apyrase per microliter. To initiate the reaction, the 18µL mixture was added to a tube containing the 2µL dNTP mix and mixed immediately by pipetting. The reaction was then incubated at room temperature for at least two minutes after which point it was mixed with an equal volume of Novex TBE-Urea Sample Buffer (2X) and resolved on a 15% Novex TBE-Urea gel.

The results show that the TdT:apyrase mixture behaves as expected: increasing level of apyrase activity leads to shorter extensions while decreasing amounts lead to longer extensions by TdT. These results also indicate that 0.25 to 1mU/µL of apyrase allows for some extension with all nucleotides. As the exact level of activity for each nucleotide should be tunable based on its own concentration, we used 0.25mU/µL or 1mU/µL of apyrase for all ensuing experiments.

In fact, we tested this tunability of extension reaction by altering both apyrase concentrations and nucleotide concentrations (**Supplementary Figs. S2B,C**). We carried out extension experiments with TdT:apyrase mix of varying apyrase concentrations with both dCTP (**Supplementary Fig. 2B**) and dGTP (**Supplementary Fig. 2C**), representing pyrimidines and purines, respectively. Each reaction was carried out in 20µL total volume. All reaction components but the dNTP were assembled in 16µL while the dNTP was prepared in 4µL of water. The 16µL mix was composed

such that upon mixing with the 4µL dNTP solution, the following initial composition would be obtained: 1X Enzymatics Green Buffer, 0.1µM LT2 initiator (**Supplementary Table 1)**, 1U/µL TdT, and 0.125, 0.25, 0.5, or 1mU/µL apyrase. The starting final concentration of substrate was varied at 5, 10, 20, 40, or 80µM for dCTP or at 1.25, 2.5, 5, 10, 20µM for dGTP. To initiate the reaction, the 16µL mixture was added to a tube containing the 4µL dNTP sample and mixed immediately by pipetting. After mixing, each reaction was incubated at room temperature for at least two minutes at which point it was mixed with an equal volume of Novex TBE-Urea Sample Buffer (2X) and resolved on a 15% Novex TBE-Urea gel.

The results show that extension lengths are tunable based on the dNTP and apyrase concentrations. Additionally, these results indicate that apyrase and dNTP concentrations have a linear inverse effect in the ranges tested. That is, doubling apyrase concentrations will have a similar impact on extension profiles as halving the dNTP concentration and vice versa. In other words, samples with a similar dCTP to apyrase ratio had similar extension lengths. For example, one can see that the following ratios yielded the same extension lengths: 20µM dCTP with 1U/µL apyrase, 10µM dCTP with 0.5U/µL apyrase, 5µM dCTP with 0.25U/µL apyrase, and 2.5µM dCTP with 0.125U/µL apyrase.

1.2 Optimizing the reaction conditions for TdT:apyrase

1.2.1 Divalent cations

The effect of divalent cations on performance of TdT has been extensively studied [1–3]. Commercially available preparations of TdT often come with one of two different buffer condition recommendations. One buffer system is based magnesium as divalent cation with the option of supplementing cobalt. The other system is based on cobalt as the sole divalent cation. We wanted to evaluate the performance of our TdT:apyrase system in all three conditions, namely, magnesium as the only divalent cation, magnesium supplemented with cobalt, and cobalt as the only divalent cation. For that, we carried out two experiments, comparing each of

magnesium with cobalt and cobalt-only conditions separately with magnesium-only condition (**Supplementary Fig. 3**).

In comparing magnesium only condition with magnesium with cobalt condition (**Supplementary Fig. 3A**), each reaction was carried out in 20µL total volume. All reaction components but the dNTP were assembled in 18µL while the dNTP was prepared in 2µL of water. The 18µL mix was composed such that upon mixing with the 2µL dNTP solution, the following initial composition would be obtained: 200µM dNTP, 1X Enzymatics Green Buffer, 0.05µM f-P5-SBS3 initiator oligo (**Supplementary Table 1)**, 250µM cobalt chloride (if present), 1U/µL TdT, and 4, 2, 1, 0.5, or 0.25 milliunits (mU) of apyrase per microliter. To initiate the reaction, the 18µL mixture was added to a tube containing the 2µL dNTP mix and mixed immediately by pipetting. The reaction was then incubated at room temperature for at least two minutes after which point it was mixed with an equal volume of Novex TBE-Urea Sample Buffer (2X) and resolved on a 15% Novex TBE-Urea gel.

While cobalt's presence affected different nucleotides differently, in general, it seemed to increase the size heterogeneity of the extended product. Importantly, in almost all reactions with cobalt, a substantial fraction of the initiator molecules remained unextended even with very low apyrase levels at which all initiator molecules are extended in the cobalt-free reaction equivalent. This observation perhaps hints at a transient structural or conformational state in the initiator or the enzyme, caused by presence of cobalt alongside magnesium, which prevents extension.

We further evaluated this supplemental cobalt effect by comparing reactions with various levels of added cobalt chloride (**Supplementary Fig. 3B**). In this experiment, each reaction was carried out in 20µL total volume. All reaction components but the dNTP and cobalt were assembled in 14µL while the dNTP and desired amount of cobalt were prepared in 6µL volume. The 14µL mix was prepared as a master mix for all reactions and composed such that upon mixing with the 6µL dNTP and cobalt solution, the following initial composition would be obtained: 300µM dATP, 0.05µM f-P5-SBS3 initiator oligo, 1X Enzymatics Green Buffer, 1U/µL TdT, 1mU/µL apyrase and 50, 100, 150, 200, 250, or 300µM cobalt chloride. To initiate the reaction, the 14µL

mixture was added to a tube containing the 6μL dATP and cobalt mixture and mixed immediately by pipetting. The reaction was then incubated at room temperature for at least two minutes after which point it was mixed with an equal volume of Novex TBE-Urea Sample Buffer (2X) and resolved on a 15% Novex TBE-Urea gel.

The results confirm that supplementary cobalt chloride results in a lack of extension in a fraction of initiators with our TdT:apyrase system. The fraction of unextended products increases with increasing cobalt chloride. This effect can be detrimental to our synthesis scheme as nucleoside triphosphates which were not added to initiators would persist even at high average extension lengths. Consequently, we decided not to use supplementary cobalt chloride in the reaction.

We next performed a series of experiments to compare magnesium-only conditions with cobalt-only conditions. We assessed extension lengths for each of the four nucleotides with TdT:apyrase enzymes in either the magnesium-based Green Buffer (Enzymatics) or cobalt-based TdT Buffer (Promega) (**Supplementary Fig. 3C**). Each reaction was carried out in 20μL total volume. All reaction components but the dNTP were assembled in 16μL while the dNTP was prepared in 4μL of water. The 16μL mix was composed such that upon mixing with the 4μL dNTP solution, the following initial composition would be obtained: 1X Green Buffer (Enzymatics B0120: 200 mM Tris-Acetate, 500 mM Potassium Acetate, 100 mM Magnesium Acetate , pH 7.9 @ 25°C) or 1X Promega TdT buffer (Promega M189A: 500mM cacodylate buffer (pH 6.8), 5mM CoCl2 and 0.5mM DTT), with 0.1μM LT2 initiator, 1U/μL TdT, and 1 mU/μL apyrase. The starting final concentration of dNTPs was varied at 25, 50, 100, 200, or 400μM for dCTP, dATP, and dTTP, or at 12.5, 25, 50, 100, or 200μM for dGTP. To initiate the reaction, the 16μL mixture was added to a tube containing the 4μL dNTP sample and mixed immediately by pipetting. After mixing, each reaction was then incubated at room temperature for at least two minutes at which point it was mixed with an equal volume of Novex TBE-Urea Sample Buffer (2X) and resolved on a 15% Novex TBE-Urea gel.

Side-by-side comparison of extension lengths with TdT:apyrase mix for each nucleotide makes a few patterns clear. First, extension with pyrimidines (dCTP and dTTP) is stimulated by cobalt as the divalent cation while extension with purines (dATP and dGTP) is hampered. This

observation is consistent with previous reports about TdT behavior [3,4]. Second, the distribution of extension lengths is wider in almost all samples with cobalt as the divalent cation. As this outcome is not favorable for our DNA synthesis scheme, we chose to use a magnesium-based buffer for our experiments.

1.2.2 Buffer and salt concentrations

Given previous results on the effect of various monovalent ions and buffering agents on TdT activity [1,4,5], we proceeded to customize our buffer to be similar to Green Buffer (**Supplementary Fig. 4A**).

Each reaction was carried out in 20µL total volume. All reaction components but the dNTP and buffer were assembled in 14µL while the dNTP and desired amount of buffer were prepared in 6µL volume. The 14µL mix was prepared as a master mix for all reactions and composed such that upon mixing with the 6µL dNTP and buffer solution, the following initial composition would be obtained: 300µM dATP, 0.05µM f-P5-SBS3 initiator oligo, 1U/µL TdT, 1mU/µL apyrase and 0.2, 0.3, 0.4, 0.5, 0.6, 0.7, 0.8, 1.0, 1.2, or 1.4X Green Buffer. To initiate the reaction, the 14µL mixture was added to a tube containing the 6µL dNTP mix and mixed immediately by pipetting. The reaction was then incubated at room temperature for at least two minutes after which point it was mixed with an equal volume of Novex TBE-Urea Sample Buffer (2X) and resolved on a 15% Novex TBE-Urea gel.

The highest extension length together with lowest product size distribution variability are observed with 0.6-0.7X Enzymatics Green Buffer. Based on these results we determined that 0.7X Enzymatics Green Buffer, composed of 14 mM Tris-Acetate, 35 mM Potassium Acetate, 7 mM Magnesium Acetate, pH 7.9, is a more optimal condition for using TdT:apyrase.

1.2.3 Additives

We also explored the effects of various additives that are commonly used to enhance the performance of various DNA polymerases or other DNA-binding enzymes, seeking to obtain more consistent performance from TdT between various nucleotides and more uniform extension

of the initiator (**Supplementary Fig. 4B**). This group of additives were glycerol, sucrose, and PEG 8000, betaine, and DMSO, Triton-X 100, and Tween 20.

Each reaction was composed of 0.1µM LT2, 0.7X Green Buffer, 125µM of each dNTP, 1U/µL TdT, and the desired amount of the additive. The additives were glycerol at 27% (v/v), sucrose at 20 and 40% (w/v), PEG 8000 at 5 and 10% (w/v), betaine at 0.5 and 1M, DMSO at 5, 10, 20, and 30% (v/v), Triton X-100 at 0.01, 0.1, 0.5, and 1.0% (v/v), and Tween 20 at 0.01, 0.1, 0.5, and 1.0% (v/v). The reaction were carried out at room temperature for 20 minutes and then mixed with an equal volume of Novex TBE-Urea Sample Buffer (2X) and resolved on a 10% Novex TBE-Urea gel.

The results show that glycerol, sucrose, and betaine have a detrimental effect, at least at the assayed concentrations, that Triton X-100 and Tween 20 slightly improve the results, and that PEG 8000 and DMSO can have a positive effect on extension by TdT, leading to longer extensions with a more homogeneous size distribution. Further experiments showed that no additional benefit can be obtained by including both PEG 8000 and DMSO in the reaction, neither does the lighter PEG 3350 outperform PEG 8000 (data not shown). Consequently, we chose to use 0.1% (v/v) Triton X-100 and 10% PEG 8000 (w/v) in later experiments.

To compare the performance of our TdT:apyrase mix in optimized buffer conditions with that in the standard conditions, we ran side-by-side extension experiments using dCTP in our optimized buffer conditions as well as the standard condition (**Supplementary Fig. 4C**). Each reaction was carried out in 20µL total volume. All reaction components but the dNTP were assembled in 16µL while the dNTP was prepared in 4µL of water. The 16µL mix was composed such that upon mixing with the 4µL dNTP solution, the following initial composition would be obtained: 0.1µM LT2 initiator, 1U/µL TdT, and 1 mU/µL apyrase in either 0.7X Green Buffer or 0.7X Green Buffer with 10% PEG 8000 and 0.1% Triton X-100. The starting final concentrations of dCTPs were 25, 50, 100, 200, or 400µM. To initiate the reaction, the 16µL mixture was added to a tube containing the 4µL dNTP sample and mixed immediately by pipetting. After mixing, each reaction was then incubated at room temperature for at least two minutes at which point it was

mixed with an equal volume of Novex TBE-Urea Sample Buffer (2X) and resolved on a 15% Novex TBE-Urea gel.

The results clearly indicate enhanced extension capacity by TdT:apyrase in our optimized buffer conditions. This enhancement is largely due to PEG 8000 increasing the effective concentrations of some reagents in the reaction.

1.2.4 Polymerase to initiator ratio

Consistent and reproducible extension of the initiator upon addition of various nucleotides in presence of apyrase demands that TdT be at saturating concentrations relative to the initiator. Subsaturation levels of TdT can result in a high extension variability, or extension of less than the maximum possible fraction of initiators upon the addition of dNTPs. With the final composition of the reaction having taken shape, we examined what levels of TdT would be saturating relative to the initiator concentrations we commonly use.

For that, we arranged a series of otherwise identical reactions with increasing TdT levels (**Supplementary Fig. 5**). Each reaction was carried out in 20μL total volume and assembled as two 10μL halves, with the first half containing dATP and TdT, and the second half containing apyrase and initiator. The two halves were composed such that upon mixing following initial composition would be obtained: 1x Custom Synthesis Buffer (14 mM Tris-Acetate, 35 mM Potassium Acetate, 7 mM Magnesium Acetate, 0.1% Triton X-100, 10% (w/v) PEG 8000, pH 7.9), 0.1μM LT2+3C initiator (**Supplementary Table 1)**, 50μM dATP, 1mU/μL apyrase, and 0.075, 0.15, 0.3, 0.6, 1.2, 2.4, 4.8, 9.6, or 19.3 U/μL TdT. To initiate the reaction, the halves were mixed quickly by pipetting. The reaction was then incubated at room temperature for at least two minutes after which point it was mixed with an equal volume of Novex TBE-Urea Sample Buffer (2X) and resolved on a 15% Novex TBE-Urea gel.

These results show that, with 0.1μM initiator oligo, concentrations of TdT above 0.6U/μL are saturating, and thus we can continue to use 1U/μL TdT in our reactions so long as initiator concentration does not surpass 0.1μM.

The experiments and optimizations above provide us with the following reaction conditions: 1X Custom Synthesis Buffer, 0.1µM (or less) initiator oligo, 1U/µL TdT (or more), and 1mU/µL apyrase.

1.3 Optimizing extension length for each nucleotide transition type

Thus far, we have examined the performance and optimized the concentrations of enzymes, buffers, salts, and other additives in our polymerization reaction. Next, we examine the substrates: initiator and nucleoside triphosphate (dNTP).

The effect of the incoming dNTP monomer on TdT polymerization rate has been extensively studied; it was observed that different nucleotides have different binding and kinetic parameters [2,4–7]. Consequently, a detailed study of the dNTP monomer effect in our system was warranted. Importantly, unlike most template-dependent polymerases, the nucleotide composition of the initiator at the 3' is also important [4,5]. This is because TdT operates in a distributive manner [2,8]; it does not remain bound to the oligonucleotide and is not processive. It also shows no preference in binding order to the initiator or the dNTP monomer. While structural studies of TdT have not identified specific polar interactions between the bases of the initiator and the enzyme [9], non-specific hydrophobic interactions do play a role. Specifically, Leucine 398 of TdT inserts itself between the last two bases of the initiator in the pre- and post-catalytic states of the enzyme, forming hydrophobic interactions with the aromatic rings of the bases and disrupting their stacking of the bases in the process. The importance of this interaction was underscored by the significant deterioration of enzyme activity upon mutating Leucine 398 to Alanine [10]. As this hydrophobic interaction will be different based on the last two nucleotides of the initiator, one would expect that both binding and kinetic parameters of the polymerization reaction to be affected by the identity of the last two nucleotides.

We, therefore, set out to simultaneously evaluate the effect of incoming dNTP monomer as well as the 3' end of the initiator on the performance of our TdT:apyrase reagent (**Fig. S6**). We performed extension experiments using the TdT:apyrase mixture on four different initiators, each

ending in a 3-nucleotide stretch of As, Cs, Gs, or Ts, using each of the four different dNTP monomers. The initiators were LT2+3A, LT2+3C, LT+3G, and LT2+3T. Each reaction was carried out in 20µL total volume. All reaction components but the dNTP were assembled in 18µL while the dNTP was prepared in 2µL of water. The 18µL mix was composed such that upon mixing with the 2µL dNTP solution, the following initial composition would be obtained: 1X Custom Synthesis Buffer, 0.1µM initiator oligo, 1U/µL TdT, and 0.25 mU/µL apyrase. The initial final concentration of dNTPs was varied at 2, 4, 8, 16, or 32µM for dCTP, dATP, and dTTP, or at 1, 2, 4, 8, or 16µM for dGTP. To initiate the reaction, the 18µL mixture was added to a tube containing the 2µL dNTP sample and mixed immediately by pipetting. The reaction was then incubated at room temperature for at least two minutes at which point it was mixed with an equal volume of Novex TBE-Urea Sample Buffer (2X) and resolved on a 15% Novex TBE-Urea gel.

These experiments show the importance of the terminal nucleotide identities to ensure non-zero initial extension to all initiators. The best results are obtained when the 3' nucleotides of the initiator are purines (As or Gs). Such initiators appear to be a good substrate for TdT and be extended completely in the TdT:apyrase reaction. The worst results are obtained with initiators ending in cytosine, which appear to be a poor substrate for the enzyme. This results in a less desirable outcome when initiators ending in Cs are being extended by anything but dCTP. In such circumstances, an initiator with an added monomer (A, T, or G) becomes a more favorable substrate than the unextended initiators, which end in Cs. As such, their subsequent extension in the same round far outpaces the extension of unextended initiators, resulting in a large size heterogeneity in extension which is undesirable for our encoding schemes. These results suggest that the order preference for TdT in the 3' end of the initiator is GGG > AAA >> TTT >> CCC. These results were also used to select the nucleotide concentrations for synthesizing H01-H12 that minimized the number of non-extended initiators for each of the twelve possible pairs of non-identical nucleotides (**Supplementary Table 4**).

1.4 Synthesizing a template sequence using optimized TdT:apyrase

Having optimized the reaction conditions for the TdT:apyrase reagent and determined the concentration required for non-zero polymerization for each incoming nucleotide based on the identity of the terminal nucleotide of the initiator, we evaluated the performance of our optimizations to synthesize a template sequence (**Supplementary Fig. 7**).

We synthesized template sequences using the TdT:apyrase mixture by cyclic addition of nucleoside triphosphates to the reaction. In one experiment, we synthesized the template sequence GATGTAGA (**Supplementary Fig. 7, left**) and in another, we synthesized the template sequence CGCACTCG (**Supplementary Fig. 7, right**). Each reaction was carried out in 100µL total volume and was mixed with a 2µL of dNTP at 50X the desired final concentration. The 100µL mix consisted of: 1X Custom Synthesis Buffer, 0.1µM initiator oligo, 1U/µL TdT, and 0.25 mU/µL apyrase. The initial final concentration of dNTP was 40µM for dATP, 200µM for dCTP, 20µM for dGTP, and 160µM for dTTP. To initiate the reaction, the 100µL mixture was added to a tube containing 2µL of the desired dNTP sample and mixed immediately by pipetting. After 1 minute incubation at room temperature, a 2µl sample of the mix was taken to be run on a gel. The remaining 100µL was added to another tube containing 2µL of the next nucleotide, mixed and incubated as before, following by collection of another 2µL sample for PAGE analysis. These steps were repeated for 8 cycles without washing, thereby extending the initiator with 8 different dNTPs while using the same enzymatic mix. Afterwards, each of the 2µL samples that were taken was mixed with an equal volume of Novex TBE-Urea Sample Buffer (2X) and resolved on a 15% Novex TBE-Urea gel.

1.5 Use of the nucleotide analogue, 5-Bromo-dCTP

We evaluated the use of 5-Bromo-dCTP (5Br-dCTP) as an alternative to natural dCTP in our synthesis reactions. Specifically, we evaluated the extension lengths, length distributions, and fraction of extended products when dCTP or 5Br-dCTP was added, using our TdT:apyrase system, to the initiator LT2+3C which ends in three cytosines.

Each reaction was carried out in 20µL total volume. Reaction components, not including the dNTP were assembled in 18µL while nucleoside triphosphates were prepared in 2µL of water. The 18µL mix was composed such that upon mixing with a 2µL nucleoside triphosphate solution, the following initial composition would be obtained: 1X Custom Synthesis Buffer, 0.1µM LT2+3C initiator, 1U/µL TdT, and 0.25 mU/µL apyrase. The initial final concentration of the dNTP was varied at 2, 4, 8, 16, or 32µM. To initiate the reaction, the 18µL mixture was added to a tube containing the 2µL dNTP sample and mixed immediately by pipetting. The reaction was then incubated at room temperature for at least two minutes at which point it was mixed with an equal volume of Novex TBE-Urea Sample Buffer (2X) and resolved on a 15% Novex TBE-Urea gel.

Our results show that the use of 5Br-dCTP for synthesis has advantages over the use of natural dCTP (**Supplementary Fig. 8**). In particular, extensions occurred at a lower concentration of 5Br-dCTP compared to natural dCTP, 2µM and 8µM respectively.

2. Codec for information storage with enzymatically-synthesized DNA

2.1 Encoder and decoder architecture

A modular design for encoding and decoding digital information in DNA is presented (**Fig. 5C, Supplementary Fig. 30**). While single monolithic architectures can be more efficient, modular designs allow for the optimization of encoding and decoding blocks separately. Such a distributed approach simplifies the design space considerably. Within individual blocks, error-correcting codes borrowed from traditional communication systems (e.g., Reed-Solomon, Fountain, BCH, LDPC) may be applied to handle multiple types of errors. Although these traditional codes may be utilized, several factors distinguish DNA storage systems from traditional systems such as wireless communication. Information is encoded into short template sequences of DNA, and must be reassembled by a decoder. Alignment errors (e.g., missing or inserted nucleotides) due to inaccurate DNA synthesis or sequencing are more difficult to correct compared to substitutions or erasures common in communication systems.

As part of a complete system for DNA storage (**Fig. 5C, Supplementary Fig. 30**), we present an encoding and decoding framework, together defined as a codec, for storing and extracting information from populations of diverse DNA strands. An important part of our encoding strategy is the placement of synchronization patterns which are regularly interspersed throughout data, allowing a decoder to compute accurate alignments from diverse, synthesized strands. Synchronization patterns are inserted in the modulation step of the encoding pipeline, which translates rows of bits into DNA sequences which adhere to modulation constraints (**Supplementary Figs. 30B, 14, Supplementary Note 2)**. The codec is also inclusive of core components such as Reed-Solomon or Fountain codes utilized in prior DNA storage systems [11–13]. The encoder first partitions data into ordered rows of bits, prefixing an address to each row to delineate its order for the purpose of reassembly. Error-correction is incorporated within each row of bits, or block of rows to protect against synthesis errors, missing sequences, or low sequencing coverage. After the unique modulation step, the encoder outputs a book of template sequences, which are written by enzymatic synthesis, yielding DNA strands. These strands can be stored. The stored strands are read by high-throughput DNA sequencing and transitions extracted to produce compressed strands, which are then fed into a decoder.

A crucial first step of the decoder is to harness information latent in diverse DNA strands by MAP estimation and probabilistic consensus (**Supplementary Fig. 30B, Supplementary Note 2**). We assume that diverse DNA strands can be sufficiently clustered (see **Supplementary Note 4**). The decoder is designed to function with minimal sequencing reads. Existing approaches for strand alignments [14–18], or greedy consensus by majority voting [19], are specialized for genomic data, and therefore do not compute optimal alignments for coded, synthetic DNA strands. Similarly, codes based on Viterbi-style recursions [20,21] do not adequately exploit the diversity of strands produced by enzymatic synthesis. By contrast, our multidisciplinary approach combines codes, multiple strand alignment, and probabilistic consensus [22]. Alignment by MAP estimation and consensus creates a complete or partial reconstruction of a template sequence from multiple strands. Subsequently, each reconstructed sequence is demodulated into bits. The final steps of the decoding pipeline are composed of decoding modules for error-correction codes which ensure error-free reconstruction of the retrieved data (**Supplementary Fig. 30B**).

In the following sections, each part of the encoder and decoder architecture (**Supplementary Fig. 30B**) is explained in greater detail. A list of parameters and design specifications for all experimental and simulated DNA storage systems is provided in **Supplementary Table 7 and Supplementary Table 8**.

2.2 Addressing of rows/sequences

The encoder (**Supplementary Fig. 30B**) first partitions data into ordered rows of bits. Each row of bits is eventually stored in one template sequence of DNA. In subsequent paragraphs, we will maintain this correspondence between rows of bits and template sequences of DNA. Each row is prefixed with a unique address to delineate its order in reassembly. Let Ω denote the total number of bits stored per row, including both payload data and addresses. Let $\mu < \Omega$ indicate the number of address bits. With μ bits, it is possible to address a total of 2^{μ} template sequences in which each template sequence stores $(\Omega - \mu)$ bits of payload data. The storage capacity is equal to the number of DNA sequences multiplied by the number of bits of payload data stored per template sequence. The storage capacity is maximized by maximizing the total number of DNA template sequences. The following equations specify the storage capacity, and the maximum theoretical storage capacity.

$$Storage\ Capacity = 2^{\mu}(\Omega - \mu)\ \text{bits, for } 0 < \mu < \Omega. \qquad (1)$$

$$Maximum\ Storage\ Capacity = 2^{\Omega-1}\ \text{bits, achieved when } \mu = \Omega - 1. \qquad (2)$$

The goal of an encoder and decoder architecture is to recover *both* the address and payload data correctly. If the address is irretrievable or only partially reconstructed, the order of information is lost. In this sense, it is more critical to recover the address. If the address is correct, it is possible to correct errors in the payload data using redundant information stored in other DNA sequences. However, in our analyses, both the address and payload information (a total of Ω bits per sequence) are decoded reliably with equal error protection.

2.3 Reed-Solomon and Fountain Codes (Block ECC)

We briefly describe Reed-Solomon (RS) codes and Fountain codes which may be incorporated within our encoding and decoding architecture (**Supplementary Fig. 30B**). However, these

codes were not explicitly used in our experiments or simulations. If synthesizing thousands or millions of template DNA sequences, error-correction across multiple sequences is necessary to protect against the following types of errors: 1) An absence of DNA strands produced for particular template sequences; 2) Low sequencing coverage for particular template sequences (i.e., too few reads of DNA strands even after PCR-amplification; 3) Bit sequences with detected bit errors after reconstruction from multiple strands, demodulation, and decoding; 4) Bit sequences with undetected bit errors after reconstruction from multiple strands, demodulation, and decoding.

In the first three cases above, the locations of bit errors are detected within a block of decoded bit sequences. If the address is intact for a bit sequence, the locations of bit errors in the payload are known. If the address is corrupted, the entire bit sequence is corrupted within a block. In the fourth case, the locations of bit errors are undetected within a block of decoded bit sequences. The fourth type of error is not possible to correct using most Fountain codes, which are specialized only to handle erased or missing bit sequences. Fountain codes were originally applied in communication networks for recovering missing packets at a high-level abstraction layer in the protocol stack. Additionally, while RS codes can correct undetected bit errors in the payload data, they require that the address per bit sequence is decoded correctly.

Within the context of DNA storage systems, an RS code is applied in the *vertical* direction across multiple rows/sequences of bits (**Supplementary Fig. 30B**). A total of Ω bits per row exist prior to RS encoding, and the same number of Ω bits per row exist after RS encoding (**Supplementary Fig. 30B**). Information in the horizontal direction is unaltered. However, the RS code inserts *extra rows* of redundant parity bits. Each extra row of parity bits contains its own unique address which is utilized by the RS decoder for error-correction.

In slightly more detail, consider an RS($n_{\mathrm{rs}}, k_{\mathrm{rs}}$) code, which has a minimum Hamming distance of $(n_{\mathrm{rs}} - k_{\mathrm{rs}} - 1)$. For this code, k_{rs} rows store address and payload bits, while $(n_{\mathrm{rs}} - k_{\mathrm{rs}})$ additional rows store RS parity bits. The RS code is able to correct up to E sequences of bits with known error locations within a block of sequences, and U sequences of bits containing undetected errors, where $2U + E \leq (n_{\mathrm{rs}} - k_{\mathrm{rs}})$. The undetected errors cost twice as much in

terms of added redundancy. An $\mathrm{RS}(n_{\mathrm{rs}}, k_{\mathrm{rs}})$ code operates over symbols from a Galois field such that in usual instantiations $n_{\mathrm{rs}} = 2^m - 1$, for $m \geq 1$ a positive integer. For example, setting $m = 8$, the well-known $\mathrm{RS}(255, 223)$ code is specified, which corrects up to 16 sequences with undetected errors within a block of sequences, or corrects up to 32 sequences with known error locations within a block. In summary, the RS code may be applied to a block of n_{rs} sequences of bits as a layer of protection against detected errors, or undetected errors if the address of bit sequences is recovered (**Supplementary Fig. 30B**).

2.4 Error-Correcting Codes per sequence

Traditional error-correcting codes (ECCs) such as Bose-Chaudhuri-Hocquenghem (BCH) codes, or low-density parity check (LDPC) codes rely on the pre-established synchronization of information. In many traditional engineered systems (e.g., wireless systems), synchronization is either assumed or resolved through various strategies. In the context of DNA storage, and within the encoder and decoder architecture of this paper (**Supplementary Fig. 30B**), synchronization is gained based on alignment to a scaffold by MAP estimation (**Supplementary Note 2**), and consensus of multiple DNA strands per template sequence. Thus, synchronization is assumed to hold prior to error-correction of bits in the decoding pipeline.

Synchronization itself is insufficient for correct decoding. For example, a missing nucleotide (deletion) in a compressed strand causes a synchronization error, but even if the position of the deletion is known via synchronization, the missing nucleotide must be recovered. The alignment and synchronization step of the decoder (**Supplementary Fig. 30B**) can resolve all errors perfectly by utilizing the *diversity* of synthesized strands per template sequence. Given sufficient diversity, the missing information in one strand variant may be recovered correctly from other variants. In this way, alignment and consensus algorithms can fully reconstruct template sequences. However, when considering scalable storage systems, a few errors may still occur after the alignment and consensus step of the decoder.

To correct errors explicitly, assuming synchronization, we apply BCH codes to encode and decode bits stored per template DNA sequence. LDPC codes could also provide similar error-correction capabilities. Primitive BCH codes are a standard class of BCH codes. Of this class, the

BCH($n_{\text{bch}}, k_{\text{bch}}, t_{\text{bch}}$) code is able to correct t_{bch} bit errors. The code takes k_{bch} bits of information as input, and outputs a total of n_{bch} bits, where ($n_{\text{bch}} - k_{\text{bch}}$) bits are added for redundancy. The BCH code is able to correct more errors if more redundancy is added.

The following BCH($n_{\text{bch}} = 31, k_{\text{bch}}, t_{\text{bch}}$) codes are able to correct t_{bch} errors:

BCH$(31, 26, 1)$; BCH$(31, 21, 2)$; BCH$(31, 16, 3)$;

The following longer BCH($n_{\text{bch}} = 63, k_{\text{bch}}, t_{\text{bch}}$) codes are able to correct t_{bch} errors:

BCH$(63, 57, 1)$; BCH$(63, 45, 3)$; BCH$(63, 39, 4)$;

BCH$(63, 36, 5)$; BCH$(63, 30, 6)$.

Similarly, the following set of BCH($n_{\text{bch}} = 127, k_{\text{bch}}, t_{\text{bch}}$) codes correct up to t_{bch} errors:

BCH$(127, 78, 7)$; BCH$(127, 71, 9)$; BCH$(127, 64, 10)$;

BCH$(127, 57, 11)$; BCH$(127, 50, 13)$.

In simulations for 0.5-megabyte, gigabyte and petabyte maximum storage capacities, we applied BCH$(31, 21, 2)$, BCH$(63, 36, 5)$, and BCH$(127, 57, 11)$ codes respectively. These BCH codes are applicable for DNA storage due to their short sequence length requirements, and efficient error-correcting abilities.

The table below summarizes the use of ECCs for simulated DNA storage systems analyzed in this paper. For experimental systems, no explicit error-correction of bits was necessary since alignment and consensus were sufficient. In simulations for 0.5-megabyte, gigabyte and petabyte maximum storage capacities, sequence ECCs were able to correct for partial alignments. Each sequence ECC inserts redundant bits per row of bits (**Supplementary Fig. 30B**). More precisely, Ω bits/row are encoded into B bits/row in the encoder, and B bits/row are decoded into Ω bits/row in the decoder. To clarify our notation, parameters Ω and B denote overall system parameters in the encoding and decoding pipeline (**Supplementary Fig. 30B**). Local parameters n_{bch} and k_{bch} for the BCH code directly affect overall system parameters. We briefly remark that this coding scheme establishes baseline efficiencies in simulations, towards a flexible-write strategy for DNA storage. The level of efficiency for coded systems could improve in the future.

ECC Per Sequence of Bits: Parameters

	Ω-Bits/Row	B-Bits/Row	ECC Per Sequence

Experiment	12	12	None
Experiment	16	16	None
Simulation	23	33	BCH(31, 21, 2)<br>2 bits uncoded
Simulation	36	63	BCH(63, 36, 5)
Simulation	57	128	BCH(127, 57, 11)<br>1 bit padding

2.5 Modulation and demodulation (Bits ⇔ Nucleotides)

A principal element of DNA storage is the encoder's mapping from bits to template nucleotides (modulation), as well as the decoder's mapping from nucleotides of reconstructed sequences to bits (demodulation). Thus, in this section we formalize the interconversion between sequences of bits and sequences of DNA nucleotides. The modulation block of the encoder (**Supplementary Fig. 30B**) maps B bits to K nucleotides: $b_1 b_2 b_3 ... b_B \Rightarrow o_1 o_2 o_3 ... o_K$. In the ideal case, one template nucleotide stores a maximum of 2 bits. Therefore, an upper bound for every modulation scheme is the limit: $B \leq 2K$. Similarly, in the decoder architecture (**Supplementary Fig. 30B**), a demodulation step maps K nucleotides to B bits: $o'_1 o'_2 o'_3 ... o'_K \Rightarrow b'_1 b'_2 b'_3 ... b'_B$. Note that the demodulation block operates on nucleotide sequences reconstructed from synthesized strands which could contain errors. Demodulation outputs a sequence of bits which may also contain errors. If no errors occur, the modulation and demodulation maps together constitute an identity map, and $b'_1 b'_2 b'_3 ... b'_B = b_1 b_2 b_3 ... b_B$. If errors do occur, separate decoding steps must provide error-correction of bit sequences (**Supplementary Fig. 30B**).

For enzymatic synthesis, $B = 2K$ is not achievable for several reasons. The controlled process of synthesis adds each nucleotide one by one. According to a specific concentration of nucleoside triphosphates, each nucleotide is added correctly in a strand^{C}, or an error such as a missing nucleotide (deletion) in a strand^{C} may occur. A current design constraint pertaining to enzymatic synthesis is to specify DNA sequences with non-identical consecutive nucleotides (e.g., without AA, TT, CC, GG transitions). Information is stored in non-identical transitions of nucleotides. Further work to account for polymerization extension lengths could remove such a constraint.

2.5.1 Nucleotide transition matrix

Constraints reflecting valid and invalid transitions between nucleotides may be expressed via a transition matrix Γ (**Supplementary Fig. 14**). An upper bound for the maximum amount of bits stored per nucleotide is $log_2 \lambda_{\max}(\Gamma)$, where $\lambda_{\max}(\Gamma)$ is the maximum eigenvalue of Γ. For enzymatic synthesis in this paper, self transitions were forbidden, leading to an upper bound of $B \leq (log_2 3)\, K$ (**Supplementary Fig. 14A**). In alternative modulation designs, minimizing the use of certain transition types, such as CA or CG, could improve synthesis accuracy but reduce the amount of information stored per template sequence (**Supplementary Fig. 14B**).

2.5.2 Synchronization nucleotides

An important aspect of the modulation step of the encoder (**Supplementary Fig. 30B**) is the insertion of synchronization nucleotides at regular intervals within each sequence. Crucially, embedded synchronization patterns provide resilience against alignment errors. The error-resilience is boosted significantly during the alignment and consensus step of the decoder (prior to the demodulation step in the pipeline). Synchronization nucleotides are also utilized in the demodulation step of the decoder (**Supplementary Fig. 30B**). As a tradeoff, the inclusion of synchronization nucleotides reduces the total space allocated for address and payload information.

2.5.3 Modulation and demodulation (Experimental)

An explicit modulation scheme for an experimental DNA storage system with parameters $(K, B) = (16, 16)$ is provided (**Supplementary Fig. 15, Supplementary Table 9**). This scheme maps $B = 16$ bits to $K = 16$ nucleotides per template sequence, while adhering to the constraint that the template sequence must consist of nucleotides with no self-transitions. Most importantly, synchronization nucleotides are embedded within each sequence. As part of the modulation, $B = 16$ bits are first converted to an intermediate form of information, which is a mixture of bits with values $\{0,1\}$ and trits with values $\{0,1,2\}$ (**Supplementary Fig. 15B, Supplementary Table 9**). The intermediate form of information is then converted to nucleotides using specified tables (**Supplementary Table 9**). According to the placement of information, each template nucleotide either stores 1 bit or 1 trit, or is selected for synchronization (**Supplementary Fig. 15A**). If synchronization nucleotides were not required, it would be possible to store up to 1.5 bits per

template nucleotide, close to the upper bound of $log_2(3)$ bits per nucleotide, by converting all input bits directly into trits. The modulation scheme for specific template sequences E1-E4 synthesized in the "Eureka!" experiment is provided in a visual diagram (**Figs. 4A**, **Supplementary Fig. 15B**).

The demodulation step of the decoding pipeline attempts to reverse the steps of modulation. With the assumption that synchronization nucleotides are provided by a prior step in the decoding pipeline, demodulation converts a sequence of nucleotides into a mixture of bits and trits, and subsequently extracts a sequence of bits according to tables of conversion (**Supplementary Fig. 15B, Supplementary Table 9**). If errors exist within the sequence of nucleotides, the demodulation step may also output a sequence of bits containing errors. Synchronization nucleotides (**Supplementary Fig. 15**) ensure that errors are localized within a sequence to some degree, limiting a propagation of errors.

2.5.4 Modulation and demodulation (Simulation)

A modulation scheme is provided (**Supplementary Fig. 15A**) for simulated DNA storage systems with parameters $(K, B) = (38, 33)$, $(K, B) = (74, 63)$, and $(K, B) = (152, 128)$. The modulation scheme for simulations is nearly identical to the modulation scheme used in the "Eureka!" experiment. It includes a similar synchronization pattern. A sequence of bits is converted to a mixture of bits and trits, and then to a sequence of nucleotides. We note that the intermediate mixture of bits and trits is designed to facilitate the placement of information between synchronization nucleotides, while also ensuring that no self-transitions are possible. The demodulation step consists of reciprocal conversions to map a sequence of nucleotides to a sequence of bits (**Supplementary Table 9**).

2.5.5 Summary of modulation and demodulation

The following table specifies the conversion of B bits per sequence to K nucleotides per template sequence for all DNA storage systems analyzed in this paper. The conversion utilizes an intermediate form of information which consists of a mixture of bits and trits. The demodulation step of the decoder reverses the steps of modulation.

Modulation and Demodulation: Design Parameters

	B-Bits/row	(Bits, Trits)	Number of sync nucleotides	K nucleotides per sequence
Experiment	12	(0, 8)	0	8
Experiment	16	(4, 8)	4	16
Simulation	33	(12, 14)	12	38
Simulation	63	(24, 26)	24	74
Simulation	128	(50, 52)	50	152

2.6 Efficiency rates for DNA storage with a digital codec

2.6.1 Efficiency rates for experimental and simulated systems

The end-to-end efficiency rate of storage may be computed for all experimental and simulated systems. Specifically, starting with Ω bits of data and addresses stored per sequence, an ECC per sequence results in B bits per sequence (**Supplementary Note 2**). Then B bits per sequence are converted and modulated into K nucleotides per sequence, including synchronization nucleotides (**Supplementary Note 2**). The following table lists these efficiencies for information storage in template DNA sequences.

Efficiency Rates for DNA Storage: Digital Codec Parameters

	Ω bits per sequence	B bits per sequence	K nucleotides per sequence
Experiment	12	12	8
Experiment	16	16	16
Simulation	23	33	38
Simulation	36	63	74
Simulation	57	128	152

2.6.2 Higher efficiency rates with increased synthesis accuracy

For our theoretical DNA storage systems, we show by simulations that efficiency rates may be increased given increased synthesis accuracy. In particular, with increased synthesis accuracy, Ω bits of data and addresses stored per sequence may be increased up to B bits per sequence.

To derive trade-offs between efficiency rates and synthesis accuracy, we modeled DNA storage as an input-output subsystem involving only a sequence of B bits (**Supplementary Fig. 30B**). Based on this abstraction, the input to our DNA storage system can be represented by a sequence of B bits prior to modulation (**Supplementary Note 2**). Similarly, the output can be represented by a sequence of B bits, obtained after demodulation. The output bit sequence may contain errors. We generated random input sequences of B bits, and obtained output sequences of B bits by simulating a subsystem within our encoding and decoding pipeline (**Supplementary Fig. 30B**). The probability of bit error, denoted by $p_{\text{bit-error}}$, was estimated by averaging over all input-output bit sequences. Assuming independent and identically-distributed symmetric bit errors, the capacity was derived to be $B(1 - h_2(p_{\text{bit-error}}))$ bits. In this capacity formula for a point-to-point bit-flip memoryless channel, $h_2(\cdot)$ denotes the binary entropy function [23]. The capacity in bits up to a maximum of B bits per template sequence was plotted for different levels of synthesis accuracy (**Supplementary Fig. 27**).

Based on the above analyses, we found that for a synthesis accuracy in which strands[C] contained ~15% missing nucleotides, a template sequence of 38 nucleotides could store 10 more bits of data and addresses, an increase from 23 to 33 bits (**Supplementary Fig. 27A**). Similarly, 27 and 70 more bits of data and addresses could be stored per template sequence of 74 and 152 nucleotides, respectively at the same level of synthesis accuracy (**Supplementary Figs. 27C, 27E**). We also tested our codec by repeating the above analyses with a combination of deletions, substitutions, and insertion errors (**Supplementary Figs. 27B, 27D, 27F**).

2.7 Alignment of DNA strands by scaffolding and consensus

Enzymatic synthesis produces populations of diverse strand variants from each DNA template sequence. The presence of diversity in DNA strands enables a larger set of strategies for synthesis, storage, and sequencing. Encoding DNA template sequences with synchronization

patterns (i.e., scaffolding) is one way to harness information from diversely synthesized strands^{C}. The term *scaffolding* is used to denote specially designed synchronization patterns in DNA sequences. This section describes algorithms for the alignment of diverse DNA strands^{C} by scaffolding and consensus.

Alignment by scaffolding and consensus is the first step of the decoding pipeline (**Fig. 5C, Supplementary Fig. 30B**). To provide a concrete framework, consider a template sequence consisting of K nucleotides that is represented by an ordered sequence of random variables $O_1O_2O_3 \dots O_K$. One particular realization of the template is denoted by $o_1o_2o_3 \dots o_K$. A decoder must decide which realization is most likely given diverse synthesized strands^{C} produced from the original sequence. The mathematical model which we adopt to model the production of DNA strands^{C} is the Markov model (**Supplementary Fig. 16A**). We will denote the ith synthesized strand^{C} by the following vector of random variables:

$$\mathbf{V}_i \triangleq \left(V_{(i,1)}, V_{(i,2)}, V_{(i,3)}, \dots, V_{(i,L_i)}\right). \tag{3}$$

The ith synthesized strand^{C} is comprised of a random number of nucleotides, and its random length is represented by random variable L_i. One particular realization of the ith synthesized strand^{C} is denoted by the following vector:

$$\mathbf{v}_i \triangleq \left(v_{(i,1)}, v_{(i,2)}, v_{(i,3)}, \dots, v_{(i,l_i)}\right). \tag{4}$$

The length l_i is a realization of the random variable L_i. Given a set $\{\mathbf{v}_i\}$ of synthesized strands^{C}, a decoder must reconstruct which original template sequence was intended for storage. This estimation is computed based on the probabilistic framework of the Markov model (**Supplementary Fig. 16A**). Such a framework is common to and adapted from the framework of synchronization codes [20].

2.7.1 Optimal alignment of diverse strands

Our method for aligning diverse strands is based on *maximum a posteriori* (MAP) estimation of each nucleotide. The optimal decoding for the k-th input nucleotide, given the set $\{\mathbf{v}_i\}$ of all synthesized output strands[C], is given by the following optimization.

$$\widehat{o_k} = \arg\ max_{o\in\{A,T,C,G\}}\ \mathbb{P}(\{\mathbf{V}_i = \mathbf{v}_i\} \mid O_k = o). \qquad (5)$$

The notation $\{\mathbf{V}_i = \mathbf{v}_i\}$ indicates a set of events occurring simultaneously. Realizations of random variables are denoted by lower-case symbols in the above formula. Associated probabilities are computed based on a Markov chain model which characterizes how synthesized DNA strands (outputs) are produced from an input sequence (**Supplementary Fig. 16A**). Decoding is aided by prior knowledge of the scaffold present in the input template sequence (**Figs. 3B, 4A, Supplementary Note 2**). The optimal alignment is computed efficiently via dynamic programming recursions if the number of strands is a small constant, and if the length of DNA sequences is short. While sequence lengths are short in DNA storage systems, the number of synthesized strands[C] per sequence may be large. Therefore, it is critical to design approximations to the above exact optimization. For future algorithmic designs, it is noted that a superior alignment may be estimated for all input nucleotides $O_1O_2O_3\ldots O_K$ jointly. However, individual probability estimates computed per nucleotide allow for the direct application of consensus rules after alignment.

2.7.2 Alignment by consensus

The optimal alignment per nucleotide given a set of synthesized strands[C] is computationally intractable for a large number of strands. Consensus-based approaches offer advantages in terms of computational efficiency. Assuming that each nucleotide in the input template DNA sequence is equally likely to have been written, and assuming that each output strand[C] is produced independently and according to identical error statistics of the Markov chain model (**Supplementary Fig. 16A**), the following product rule may be applied for probabilistic consensus [22]:

$$\widehat{o_k} = \arg max_{o\in\{A,T,C,G\}} \prod_i \mathbb{P}(\mathbf{V}_i = \mathbf{v}_i \mid O_k = o). \tag{6}$$

The above product rule may be derived from Bayes' theorem directly, and is related to a simple Bayes classifier. Consensus optimization is computed efficiently via dynamic programming recursions, and remains tractable even for an increasing number of strand variants. Its computational complexity scales linearly in the number of strands^{C}. The key difference between the above consensus product rule and the optimal solution of alignment is that the inner probability only involves a single strand^{C}, as opposed to all strands^{C} jointly. As the number of strands^{C} increases, the inner probability may be computed for each strand^{C} separately and efficiently, after which a product consensus rule is applied.

It is computationally feasible to reconstruct an optimal alignment by scaffolding for a small group of strands^{C}, and then apply consensus over disjoint groups. For our "Eureka!" experiment, in which template sequences each contained 16 nucleotides, we first aligned strands optimally in two disjoint groups, each containing 5 strands^{C} each. We then applied product-wise consensus over the two groups. For our simulated DNA storage systems, we applied optimal alignment to groups containing 2 strands^{C} each, followed by product-wise consensus over 5 groups. In both experiments and simulations, a total of 10 filtered strands^{C} were utilized for decoding. We note that for aligning multiple strands, there are several ways to combine pairwise or groupwise alignments. Optimal alignment remains computationally intractable. However, several low-complexity relaxations are possible. Advancements to the field of bioinformatics are anticipated to continually improve the quality of alignments.

2.7.3 Markov chain model

The Markov chain model (**Supplementary Fig. 16A**) provides a probabilistic framework for computing alignments and forming consensus. To simplify calculations, it is possible to "unwrap" the model. As a first step, we assume $p_{\mathrm{bur}} = 0$, and focus exclusively on deletion, insertion, and substitution events. We limit the number of insertions per sequence position to two. In experiments, the number of insertions at a given sequence position was rarely beyond

two. Other related errors such as replication error, defined as a repetition of a short motif pattern, could be modeled. We first define the event of nucleotide synthesis or "transmission" using terminology of a storage channel. The synthesis of a nucleotide results in either a correct write or a write error. The probability of a nucleotide synthesis (a write or write error) is defined as follows: $p_{\text{syn}} \triangleq 1 - p_{\text{ins}} - p_{\text{del}}$. Then, the following table of probabilities indicates six events possible in an "unwrapped" and simplified Markov model (**Supplementary Fig. 16A**).

"Unwrapped" Markov Chain Model: Probability of Events

**Probability**	**Description of Event**
$p_1 \triangleq p_{\text{del}}$	Deletion (Missing Nucleotide)
$p_2 \triangleq p_{\text{syn}}$	Synthesis Error (Write Error)
$p_3 \triangleq p_{\text{ins}}\, p_{\text{del}}$	Insertion and Deletion
$p_4 \triangleq p_{\text{ins}}\, p_{\text{syn}}$	Insertion and Synthesis
$p_5 \triangleq \frac{(p_{\text{ins}})^2}{1 - p_{\text{ins}}}\, p_{\text{del}}$	Two Insertions and Deletion
$p_6 \triangleq \frac{(p_{\text{ins}})^2}{1 - p_{\text{ins}}}\, p_{\text{syn}}$	Two Insertions and Synthesis

Each event occurs with a specified probability. Probabilities p_5 and p_6 are modified slightly to ensure that the total sum of probabilities in this simplified model is:

$$p_1 + p_2 + p_3 + p_4 + p_5 + p_6 = 1. \tag{7}$$

2.7.4 Forward-Backward recursions

In the following section, we describe how to compute the probability $\mathbb{P}(\mathbf{V}_i = \mathbf{v}_i \mid O_k = o)$ efficiently using α/β forward-backward recursions (**Supplementary Figs. 16, 17**) [20].

For the ith synthesized strand, define the event $Q_{i,s,t}$ to represent that t nucleotides were correctly added after s synthesis steps. Using the notation $[a{:}\,b]$ to represent all indices between a and b including endpoints, we define the following probabilities:

$$\alpha_{(i,s,t)} \triangleq \mathbb{P}\big(\mathbf{V}_{(i,[1:\,t])} = \mathbf{v}_{(i,[1:\,t])}, Q_{(i,s,t)}\big); \tag{8}$$

$$\beta_{(i,s,t)} \triangleq \mathbb{P}\big(\mathbf{V}_{(i,[t+1:\,l_i])} = \mathbf{v}_{(i,[t+1:\,l_i])}, Q_{(i,K,l_i)} \big| Q_{(i,s,t)}\big)\,. \tag{9}$$

The α/β probabilities may be computed via forward-backward recursions. Denote a uniform probability over the DNA alphabet for nucleotides by $p_u = 1/4$. To quantify a substitution error in the calculations, we define the following function for inputs $x, y \in \{A, T, C, G\}$ (**Supplementary Fig. 16B**):

$$\phi(x, y) \triangleq 1 - p_{\text{sub}}, \text{if } x = y; \tag{10}$$

$$\phi(x, y) \triangleq \left(\frac{1}{3}\right) p_{\text{sub}}, \text{if } x \neq y. \tag{11}$$

Dynamic programming is designed to utilize pre-existing computations in a recursive manner. To compute $\alpha_{(i,s,t)}$, a two-dimensional table of probabilities is populated in the "forward" direction. To compute $\beta_{(i,s,t)}$, a two-dimensional table of probabilities is populated in the "backward" direction. The following table summarizes the recursive computations required. The sum of the probabilities in each column of the table yields $\alpha_{(i,s,t)}$ and $\beta_{(i,s,t)}$ respectively. A basic example of computing α/β forward-backward probabilities is provided as a visual diagram assuming that $p_{\text{ins}} = 0$ (**Supplementary Fig. 17**).

Forward-Backward Recursions: Calculation of Probabilities

**Forward Probability:** $\alpha_{(i,s,t)}$	**Backward Probability:** $\beta_{(i,s,t)}$
$p_1 \alpha_{(i,s-1,t)}$	$p_1 \beta_{(i,s+1,t)}$
$p_2 \alpha_{(i,s-1,t-1)} \sum_o \mathbb{P}(O_s = o)\, \phi(v_{(i,t)}, o)$	$p_2 \beta_{(i,s+1,t+1)} \sum_o \mathbb{P}(O_{s+1} = o)\, \phi(v_{(i,t+1)}, o)$
$p_3 p_u \alpha_{(i,s-1,t-1)}$	$p_3 p_u \beta_{(i,s+1,t+1)}$
$p_4 p_u \alpha_{(i,s-1,t-2)} \sum_o \mathbb{P}(O_s = o)\, \phi(v_{(i,t)}, o)$	$p_4 p_u \beta_{(i,s+1,t+2)} \sum_o \mathbb{P}(O_{s+1} = o)\, \phi(v_{(i,t+2)}, o)$
$p_5 {p_u}^2 \alpha_{(i,s-1,t-2)}$	$p_5 {p_u}^2 \beta_{(i,s+1,t+2)}$
$p_6 {p_u}^2 \alpha_{(i,s-1,t-3)} \sum_o \mathbb{P}(O_s = o)\, \phi(v_{(i,t)}, o)$	$p_6 {p_u}^2 \beta_{(i,s+1,t+3)} \sum_o \mathbb{P}(O_{s+1} = o)\, \phi(v_{(i,t+3)}, o)$

2.7.5 Decoding based on alignment of multiple DNA strands

Once the forward-backward probabilities have been computed, it is straightforward to compute $\mathbb{P}(\mathbf{V}_i = \mathbf{v}_i \mid O_k = o)$. Here, we simplify our presentation by assuming that $p_{\text{ins}} = 0$, and only consider cases for deletions and substitutions. Using the α/β probabilities,

$$\mathbb{P}(\mathbf{V}_i = \mathbf{v}_i \mid O_k = o) = \sum_{t=0}^{l_i} p_1\, \alpha_{(i,k-1,t)}\, \beta_{(i,k,t)} + \sum_{t=0}^{l_i} p_2\, \alpha_{(i,k-1,t-1)}\, \beta_{(i,k,t)}\, \mathbb{P}(O_k = o)\phi\big(v_{(i,t)}, o\big). \tag{12}$$

A basic example of MAP estimation by scaffolding is provided (**Supplementary Figs. 16, 17**). Decoding by alignment is possible because of the synchronization pattern embedded as a scaffold in the template sequence. The probability $\mathbb{P}(O_k = o)$ is either exactly one at the k-th input position if the synchronization nucleotide is correctly placed, otherwise it is exactly zero. The synchronization nucleotides provide strong cues for the correct placement of other nucleotides. In addition to the above probability for computing $\mathbb{P}(\mathbf{V}_i = \mathbf{v}_i \mid O_k = o)$, it is also possible to compute optimal pairwise and groupwise alignments. For example, for the ith synthesized strand and j^{th} synthesized strand considered jointly together, the α/β probabilities include $\alpha_{(i,s,t)}$ and $\beta_{(i,s,t)}$ as well as $\alpha_{(j,s,t)}$ and $\beta_{(j,s,t)}$. The following probability for optimal pairwise alignment may be computed via these α/β probabilities: $\mathbb{P}\big(\mathbf{V}_i = \mathbf{v}_i, \mathbf{V}_j = \mathbf{v}_j \mid O_k = o\big)$. In a similar manner, groupwise alignment from three strands may be computed: $\mathbb{P}\big(\mathbf{V}_i = \mathbf{v}_i, \mathbf{V}_j = \mathbf{v}_j, \mathbf{V}_q = \mathbf{v}_q \mid O_k = o\big)$.

2.7.6 Computational complexity of optimal vs. consensus alignment

With a slight change of notation, consider that the average length of all synthesized strands[C] is given by L. To compute the α/β probabilities for each synthesized strand[C] requires approximately $O(LK)$ time complexity. Similarly, to compute the optimal alignment from just one synthesized strand[C], approximately $O(LK)$ time complexity is necessary. For optimal pairwise alignment, approximately $O(L^2K)$ time complexity is necessary. The complexity increases at least exponentially in L with the exponent equal to the number of synthesized strands[C]. By contrast, alignment by consensus incurs a computational complexity which scales

linearly in L with the number of synthesized strands[C]. Therefore, it is critical to utilize approximations such as fast consensus methods to align multiple strands[C].

2.7.7 Alternative alignment algorithms

The use of dynamic programming is one solution for computing alignments of synthesized strands[C]. Another algorithm for alignment, termed majority voting alignment, consists of greedy consensus [19]. We found that such an algorithm was not sufficient to correct a large number of errors such as missing nucleotides, given only 10 filtered strands[C] (**Supplementary Fig. 29**). However, majority voting alignment may be combined with codes such as repetition coding to correct a larger number of errors. A full analysis of a coded form of majority voting alignment is an interesting direction to explore for future algorithmic designs.

2.7.8 Considerations of increased diversity for consensus

Alignment by consensus is not always beneficial. To be precise, we consider the following mathematical problem for consensus— deciding a single bit of information by forming a consensus from multiple independent estimates. We denote R estimates by independent Bernoulli random variables $\{Z_r\}_{r=1..R}$, which are one with probability η, indicating an error, and zero with probability $(1-\eta)$, indicating a correct estimate. If each estimate is correct more than half the time, i.e., $\eta < 1/2$, then the following proposition provides an upper bound for the probability of a majority error formed by majority consensus using R estimates. For notational purposes, a binary divergence function is defined for parameters $a, q \in [0, 1]$.

$$D_{\text{div}}(a\|q) \triangleq \ a\, ln\frac{a}{q} + (1-a)\, ln\frac{1-a}{1-q}. \tag{13}$$

$$\textbf{Proposition}: Probability\ of\ Majority\ Error \leq exp\left(-R\, D_{\text{div}}\left(\frac{1}{2} \parallel \eta\right)\right). \tag{14}$$

Proof of Proposition. Chernoff's bound states that for R independent and identically-distributed Bernoulli random variables $\{Z_r\}_{r=1..R}$, where each random variable is one with probability η,

$$\mathbb{P}\left(\sum_{r=1}^{R} Z_r \geq \frac{R}{2}\right) \leq exp\left(-R\, D_{\text{div}}\left(\frac{1}{2} \parallel \eta\right)\right). \quad (15)$$

This above probability corresponds to the probability of majority error since the sum of Bernoulli random variables indicates that the consensus estimate is incorrect. If the sum exceeds more than half of the votes, a majority error occurs.

Interpretation. The probability of majority error decreases exponentially with the number of estimates R, as long as each estimate is correct more than half the time. However, if each individual estimate is not reliable, this exponential effect is not guaranteed. Consensus improves decoding accuracy as long as each estimate is reasonable, reinforcing information as opposed to contributing noise.

3. Diversity analyses of enzymatic synthesis

Enzymatic synthesis of a template sequence produces raw strands (strands^{R}) with variable extension length per nucleotide. From each raw strand^{R}, transitions can be extracted to form compressed strands (strands^{C}). Each strand^{C} may be of variable length. For subsequent analyses in this section, we model the distribution of strand^{C} lengths, and compute the number of diverse strand^{C} variants of each length. We also provide edit distances between synthesized strand^{C} variants and the original template sequence, along with a detailed error analyses.

3.1 Distribution of lengths for compressed DNA strands

Synthesis errors resulting in missing nucleotides (deletions), or insertions directly affect the length of a strand^{C}, unlike conventional errors such as substituted (mismatched) nucleotides. We constructed a mathematical model for nucleotide errors occurring in synthesized strands^{C}. Our model is a Markov model (**Supplementary Fig. 16A**) with a state space indicating different types of nucleotide errors such as missing nucleotides (deletions), substituted nucleotides, and insertions. It is assumed that each strand^{C} variant is synthesized independently and according to identical error statistics, as specified in the Markov model (e.g., $p_{\text{del}}, p_{\text{sub}}, p_{\text{ins}}$). The error process results in several unique synthesized strands^{C}. These strands^{C} can be aligned to reconstruct the original sequence. While reconstruction is possible through alignment and probabilistic consensus, often the exact determination of error events in strands^{C} is ambiguous.

For example, a random insertion followed by a deletion of an intended nucleotide is indistinguishable from a substitution error (**Supplementary Fig. 16A**).

Despite inherent ambiguities in the error process, it is possible to derive the length distribution of strands^{C} based on the Markov model (**Supplementary Fig. 16A**). Consider a template DNA sequence $o_1 o_2 o_3 ... o_K$ comprised of K nucleotides. A correct write in the k-th position results in one correct nucleotide added. However, missing nucleotides and insertions affect the total length of strands^{C} produced (**Supplementary Fig. 16A**). Let T denote a discrete random variable representing the number of nucleotides added in the k-th position. The read-length distribution of strands^{C} is derived by specifying the statistics of random variable T. The probability mass function of T is denoted by $P_T(t)$. The probability generating function is a formal power series defined as follows,

$$G_T(\omega) \triangleq \sum_{t=0}^{\infty} \omega^t P_T(t). \tag{16}$$

The following proposition expresses the generating function in closed form, from which the moments of T may be derived.

$$\textbf{Proposition}: G_T(\omega) = \frac{p_{\text{del}}}{1 - p_{\text{ins}}\omega} + \frac{\omega(1 - p_{\text{del}} - p_{\text{ins}})(1 - p_{\text{bur}})}{(1 - p_{\text{bur}}\omega)(1 - p_{\text{ins}}\omega)}. \tag{17}$$

Proof of Proposition. Consider a random variable U which represents the number of nucleotides written starting from either the WRITE state or the WRITE ERROR state (**Supplementary Fig. 16A**). Then U is a geometric random variable with probability mass function given by,

$$P_U(0) = 0, \text{and } P_U(u) = (p_{\text{bur}})^{u-1}(1 - p_{\text{bur}}) \text{ for } u \geq 1. \tag{18}$$

The generating function of geometric random variable U is given by,

$$G_U(\omega) \triangleq \sum_{u=0}^{\infty} \omega^u P_U(u) = \frac{\omega(1 - p_{\text{bur}})}{1 - p_{\text{bur}}\omega}. \tag{19}$$

Based on the Markov model, the probability mass function of T is defined recursively.

$$P_T(0) = p_{\text{del}}, \text{and } P_T(t) = p_{\text{ins}} P_T(t-1) + (1 - p_{\text{del}} - p_{\text{ins}}) P_U(t) \text{ for } t \geq 1. \quad (20)$$

The generating function of T is derived starting from its power series representation.

$$G_T(\omega) \triangleq \sum_{t=0}^{\infty} \omega^t P_T(t) = p_{\text{del}} + p_{\text{ins}} \omega G_T(\omega) + (1 - p_{\text{ins}} - p_{\text{ins}}) G_U(\omega). \quad (21)$$

Solving for $G_T(\omega)$ in the above equation establishes the proposition. Based on the generating function, the moments of T may be computed. For example, the mean $E[T] = G'_T(\omega = 1)$ depends on the first derivative of the generative function, and the variance $VAR[T] = G''_T(\omega = 1) + G'_T(\omega = 1) - (G'_T(\omega = 1))^2$ depends on the second derivative of the generating function.

3.1.1 Exact length distribution (Theory)

The probability mass function for random variable T describes the statistics for writing one nucleotide of a template sequence to create a strand^C. There exist K nucleotides in the template sequence $o_1 o_2 o_3 ... o_K$. The length of a synthesized strand^C is also a random variable, which we denote here by L. The length L has a probability mass function $P_L(l)$. Assuming each write is independent of previous and future writes, the generating function for length L is given by,

$$G_L(\omega) = (G_T(\omega))^K. \quad (22)$$

3.1.2 Binomial distribution (Special case)

As a special case, assume that only deletions occur in the Markov model (**Supplementary Fig. 16A**) so that $p_{\text{ins}} = p_{\text{sub}} = p_{\text{bur}} = 0$. Then, the generating function, mean, and variance of the length L are given as follows.

$$G_L(\omega) = (p_{\text{del}} + \omega(1 - p_{\text{del}}))^K. \quad (23)$$

$$E[L] = K(1 - p_{\text{del}}). \quad (24)$$

$$VAR[L] = K p_{\text{del}} (1 - p_{\text{del}}). \quad (25)$$

As expected, such a generating function is recognized as corresponding to the well-known binomial distribution. More precisely, the binomial distribution of the length is given by,

$$P_L(l) = \left(\frac{K!}{l!\,(K-l)!}\right)(p_{\mathrm{del}})^{K-l}(1-p_{\mathrm{del}})^{l} \text{ for lengths in the range } 0 \le l \le K. \quad (26)$$

3.1.3 Experimental distribution of length

The length distribution was observed empirically in data for all synthesized strands^{C} produced by enzymatic synthesis for template sequences E1-E4, each containing $K = 16$ nucleotides (**Supplementary Fig. 20A**). More than $100{,}000$ raw strands^{R} were sequenced for each of E1-E4 and post-processed *in silico* to obtain run-length compressed strands^{C}. From data histograms (**Supplementary Figs. 21-25**), it was verified that mostly deletions (missing nucleotides) occur in compressed strands^{C}. Strands^{C} were further aligned to their respective template sequences E1-E4 using the Needleman–Wunsch algorithm [14], verifying the presence of missing nucleotides (**Supplementary Fig. 19A**).

Assuming primarily deletions in all strands^{C}, the empirical length distribution may be compared to a fitted binomial distribution. To fit a modified binomial distribution, we define two probability mass functions as follows.

$$P_L(l) = \left(\frac{K!}{l!\,(K-l)!}\right)(p_{\mathrm{del}})^{K-l}(1-p_{\mathrm{del}})^{l}, \text{for read lengths in the range } 0 \le l \le K. \quad (27)$$

$$Q_L(l) = \frac{1}{5}, \text{for read lengths in the range } 0 \le l \le 4. \quad (28)$$

The distribution $Q_L(l)$models the non-negligible uniform probability that very short strands^{C} are produced. We fitted a mixture probability distribution for strands^{C} lengths (**Supplementary Fig. 20A**) of the following form: $0.2Q_L(l) + 0.8P_L(l)$. For the set of all synthesized strands^{C}, the fitted binomial parameter $p_{\mathrm{del}} = 0.59$ for the E1 template, $p_{\mathrm{del}} = 0.55$ for the E2 template, $p_{\mathrm{del}} = 0.65$ for the E3 template, and $p_{\mathrm{del}} = 0.56$ for the E4 template. Size selection processes, performed *in silico* or *in vitro*, to keep only longer synthesized strand^{C} variants decrease the effective number of missing nucleotides to be resolved. We further purified strands^{C} *in silico* which eliminated very short strands^{C}, resulting in a binomial distribution of the form: $P_L(l)$, in

which we determined that the average probability of deletion was $p_{\text{del}} = 0.28$ (**Supplementary Fig. 19B, 20B**). Thus, size-selection of strand^{C} variants led to a reduction in the effective probability of missing nucleotides.

3.2 Diversity of synthesized strands

Enzymatic synthesis not only produces strands^{C} of different lengths, but also diverse strands^{C}. Each strand^{C} may contain errors such as missing nucleotides in different positions relative to its corresponding template sequence. In this section, we compare the theoretical diversity with the experimentally observed diversity produced by enzymatic synthesis.

3.2.1 Mathematical diversity

To simplify analysis, we consider the case of only missing nucleotides occurring in strand^{C} variants. A *loose upper bound* for the number of diverse variants of length l derived from a template sequence of length Knucleotides is given by,

$$Diversity[l, o_1 o_2 o_3 \dots o_K] \le \left(\frac{K!}{l!\,(K-l)!}\right) \text{ for } 0 \le l \le K. \quad (29)$$

This upper bound is equivalent to the total number of strands^{C} of length l obtained after $(K - l)$ deletion errors, and is independent of the template sequence itself.

An accurate count of diversity must include only distinct strand^{C} variants. Thus, diversity depends on the specific template sequence $o_1 o_2 o_3 \dots o_K$ being synthesized. For each template sequence E1-E4 and also E0, we computed the number of subsequences possible for each length l. For the E0 template, a total of 36909 subsequences exist of all lengths of which 18233 do not include self-transitions. For the E1 template, a total of 15863 subsequences exist of all lengths of which 1799 do not contain self-transitions. For the E2 template, a total of 29910 subsequences exist of all lengths of which 10795 do not contain self-transitions. For the E3 template, a total of 24960 subsequences exist of all lengths of which 6469 do not contain self-transitions. Finally, for the E4 template, a total of 23679 subsequences exist of all lengths of which 6487 do not contain self-transitions. We note that the E1 template (CATATCACATCTCACT) does not contain a 'G' nucleotide, which is why the number of distinct subsequences with no self-transitions is much

less in comparison. These computations determine the theoretical maximum number of possible strand^C variants produced when synthesizing each template sequence (E0-E4).

3.2.2 Empirical diversity of DNA strands

We observed the empirical diversity of all strand^C variants based on reads from more than 100,000 raw strands^R synthesized from templates E0-E4 (**Supplementary Fig. 20**). The number of unique strand^C variants of each length was compiled for all strands^C as well as purified strands^C. As a summary, the total diversity count of strands^C of all lengths for each template sequence E0-E4 is provided in the following table.

Total Empirical Diversity of Strands^C for E0-E4
(Strands^C with 16 nucleotides or less)

	E0	E1	E2	E3	E4
Unique strands^C with reads ≥ 3	2911	1163	1812	1792	2122
Unique strands^C with reads ≥ 10	1120	468	803	855	982
Unique purified (size selected) strands^C with reads ≥ 1	442	458	410	460	442
Unique purified (size selected) strands^C with reads ≥ 2	156	162	144	164	173

From the first two rows of the above table, we observed that the total empirical diversity counts of all strands^C produced from E0-E4 were ~2-6 fold less than the theoretical limit calculated. However, as shown in our error analyses of edit distances between strands^C and template sequences (**Supplementary Fig. 20A**), as well as more detailed error analyses (**Supplementary Fig. 19A**), not all strands^C have adequate lengths. The last two rows of the above table indicate diversity counts for purified (size-selected) strands^C which contain fewer missing nucleotides and errors (**Supplementary Figs. 19B, 20B**). Even after purification, a sufficient number of diverse strands exist that may be harnessed for sequence reconstruction.

4. Factors impacting scalable DNA storage systems

4.1 Four main factors affecting scalability

The scalability of DNA information storage systems is dependent on four main factors. We rank these factors in order of importance, although they are closely interconnected. For our analysis, we recall that the storage capacity is given by $2^{\mu}(\Omega - \mu)$ bits where μ is the number of address bits per DNA sequence, and Ω is the total number of address and data bits stored per DNA sequence (**Supplementary Note 2**).

4.1.1 Cost of synthesis

While megabytes of data have been stored in DNA, using approximately 1-10 million synthesized DNA sequences, the current cost of synthesis based on phosphoramidite chemistry is ~$3500/megabyte [11]. Accordingly, storing gigabytes of data in DNA would require an exorbitant amount of money, above $1,000,000. Our enzymatic DNA synthesis projects to decrease reagent costs by several orders of magnitude as reactions are miniaturized (**Supplementary Note 6**). Such a reduction in synthesis costs will facilitate affordable storage towards the goal of large-scale storage of data in DNA.

4.1.2 Number of template sequences that can by synthesized

Even with low-cost synthesis, a significant challenge for large-scale storage is the massive number of DNA sequences that must be synthesized. As an example, consider a storage system with DNA sequences containing 200 nucleotides. In such a system, the maximum efficiency rate of 2 bits per nucleotide is possible. In this ideal case, 400 bits ($\Omega = 400$) may be stored per DNA sequence. If $2^{\mu} = 2^{20} \approx 1$ million sequences are synthesized, then parameters $(\mu, \Omega) = (20, 400)$, and the storage capacity is 47.5 megabytes. If $2^{\mu} = 2^{30} \approx 1$ billion template sequences are synthesized, then parameters $(\mu, \Omega) = (30, 400)$, and the storage capacity is 46.25 gigabytes. Thus, a 1000-fold increase in the number of DNA sequences synthesized yields a proportional increase in storage capacity. However, synthesizing a large number of DNA sequences in massively parallel synthesis reactions remains an engineering challenge.

4.1.3 Number of nucleotides per template sequence

Increasing the number of nucleotides per template sequence will increase storage capacity. Consider two storage systems, one with sequences of 200 nucleotides, and the other with sequences of 400 nucleotides. Further, assume a theoretically maximum efficiency rate of 2 bits stored per nucleotide. For the first system, setting parameters $(\mu, \Omega) = (20, 400)$, the maximum storage capacity is 47.5 megabytes. For the second system, setting parameters $(\mu, \Omega) = (20, 800)$, the maximum storage capacity is 97.5 megabytes. Thus, increasing the number of nucleotides per sequence from 200 to 400 results in a 2-fold increase, a linear scaling, of storage capacity. As enzymatic synthesis accuracy improves, through process engineering or advances in biochemistry, the number of nucleotides per DNA sequence can be increased to achieve larger storage capacities.

4.1.4 Efficiency rate of storage

Increasing the efficiency rate of storage will increase the storage capacity. Consider two storage systems, both with sequences containing 200 nucleotides. One system achieves a theoretically maximum efficiency rate of 2 bits per nucleotide, while the other achieves an efficiency rate of 0.5 bits per nucleotide. For the first system, the storage capacity is 47.5 megabytes (setting $(\mu, \Omega) = (20, 400)$). For the second system, which synthesizes the same number of sequences, the storage capacity is 10 megabytes (setting parameters $(\mu, \Omega) = (20, 100)$). Thus, reducing efficiency rate by 4-fold leads to a nearly linear reduction in storage capacity. Efficiency rates of storage can be increased with improvements to enzymatic synthesis accuracy, which will reduce the amount of redundancy overhead required for error-correction.

Based on these analyses, we consider the two immediate challenges for large-scale storage in DNA to be the cost of synthesis and the massive parallelization of affordable synthesis reactions.

4.2 Soup of DNA: DNA storage modeled as a permutation channel

Consider the storage capacity of a DNA storage system consisting of $M = 2^{\mu}$ template DNA sequences. Assuming that each sequence contains K nucleotides, the following proposition expresses an upper bound for the total number of bits possible for storage. The result is obtained by modeling DNA storage as a permutation channel.

Proposition: *Define* $C_{\text{cap}}[M, K]$ *as the storage capacity.*

$$C_{\text{cap}}[M, K] \leq log_2 \left(\frac{(M + 4^K - 1)!}{(4^K - 1)!\, M!} \right) bits. \tag{30}$$

Proof of Proposition. We first present a related mathematical problem. Consider a set $\{x_i\}$ for $i \in \{1, 2, \ldots, J\}$ where $x_i \geq 0$ are integers, and consider the following equation:

$$\sum_{i=1}^{J} x_i = \Lambda. \tag{31}$$

The variables J and Λ are also integers. It is known that the equation has exactly $\frac{(\Lambda+J-1)!}{(J-1)!\Lambda!}$ integral solutions. To analyze information storage in DNA as a permutation channel, it is noted that DNA strands lose their relative order when synthesized and mixed in solution. Information is only preserved and conveyed in the form of the type of nucleotide sequence. For a sequence of length K nucleotides, there exist 4^K unique sequence types possible. Since there exist M sequences, the storage capacity upper bound is computed by setting $J = 4^K$ and $\Lambda = M$, indicating the number of different patterns possible in DNA using M sequences for storing data. Taking the $log_2(\cdot)$ function over the total number of distinguishable patterns yields total bits stored.

Special Cases. The following special cases further illustrate the upper bound.

$$C_{\text{cap}}[M, 1] \leq log_2 \frac{(M + 3)!}{3!\, M!} = O(log_2 M). \tag{32}$$

$$C_{\text{cap}}[1, K] \leq 2K. \tag{33}$$

$$C_{\text{cap}}[M, log_2 M] \leq log_2 \frac{(M^2 + M - 1)!}{M!\,(M^2 - 1)!} = O(M\, log_2 M). \tag{34}$$

The first inequality states that the number of nucleotides per sequence must increase, and not be held constant, to store enough bits. The third inequality states that the number of nucleotides per sequence must increase at least by $O(log_2 M)$, in order to increase storage capacity adequately. In terms of bits, the requirement for address space is also $O(log_2 M)$ bits per sequence. If M grows at the very least proportional to K, then at least $O(log_2 K)$ bits per sequence must be

allocated for addresses. Thus, $O(log_2 K)$ bits per sequence can be taken as a trivial minimum lower bound for storage requirements (**Fig. 5A**). In general, we note that the number of sequences M must grow much faster than sequence length K, but cannot in practice grow at an exponential scale if synthesized using current technologies.

4.3 Clustering of DNA strands

Although not extensively analyzed in this manuscript, an important part of a codec for DNA storage is a module for clustering reads of DNA strands^{C} produced by synthesis (**Supplementary Fig. 30A**). Ideally, all DNA strands^{C} produced from the same template sequence should be clustered together. A clustering module is assumed in our detailed codec architecture (**Supplementary Fig. 30B**).

4.3.1 Clustering DNA strands based on relative edit distances

In our experiments with template sequences of lengths $K = 8$ and $K = 16$, the cluster for synthesized strands^{C} is uniquely identified by the first 3 correctly-synthesized nucleotides. In a properly designed codec accommodating template sequences of increasing length K, the relative edit distance between strands^{C} produced from different template sequences should increase. To see this, consider that there exist 4^K distinct DNA template sequences of length K. However, far less than an exponential number of template sequences can be synthesized in practice. If a sequence ECC is utilized prior to mapping from bit sequences to DNA template sequences, the ECC automatically ensures that different bit sequences have a minimum Hamming distance relative to each other. For example, a BCH code with t_{bch} error-correcting ability maintains a minimum Hamming distance of $(2t_{\mathrm{bch}} + 1)$ between bit sequences. These bit sequences are modulated into DNA template sequences, resulting in a corresponding separation in terms of Levenshtein edit distance. We refer to prior established work for clustering billions of reads of DNA strands based on relative edit distances [13]. While computing exact edit distances between all DNA strands^{C} can be computationally expensive, efficient distributed clustering algorithms have been derived recently based on approximate edit distances [24].

4.3.2 Alternative methods for clustering DNA strands

Clustering of reads may be achieved directly by allocating space in each template sequence specifically to help distinguish and classify resulting synthesized strands^C. For example, if an address is stored per template sequence and highly protected with extra redundancy, it may be decoded separately in each strand^C. In such a design, explicit clustering of strands^C is not necessary, since each strand^C is uniquely identified by its address [12]. The disadvantage of this approach is the reduction in the efficiency rate of storage to ensure correct decoding of the address. Other methods for clustering could involve markers or detectable patterns embedded in the template sequence, similar to synchronization patterns.

5. 2D array-format synthesis

We sought to translate our bead-based process to create an array-based enzymatic DNA synthesis platform. Our prototype is comprised of two main parts: a Mantis liquid handler, which has a single robotic arm that can be programmed to dispense one of six reagents at a time, and custom jigs, which were either laser cut (Epilog Legend 36EXT) or machined (gift from Formulatrix) to hold the glass slide acting as a solid support substrate for the DNA (**Supplementary Fig. 31A**).

5.1 Initiator immobilization and surface preparation

We covalently attached a 5’ amine-modified initiator oligo (5Am12-fSBS3-ctgag) and a 3’ amine-modified blocking oligo (10T-3Am) (**Supplementary Table 1**) onto an aldehydesilane-coated microscope slide (Schott Nexterion Slide AL). The blocking oligo was included to prevent unwanted interactions, such as adsorption, between the initiator or enzymes to the surface. To do this, we created an oligo mixture containing 2µM 5Am12-fSBS3-ctgag and 8uM 10T-3AM in 3X SSC (1X SSC is 150mM NaCl and 15mM sodium citrate) and 1.5M Betaine. The oligo mixture was dispensed as 0.1µL droplets onto the slide using a Mantis liquid handler (**Supplementary Fig. 31B, C**). Following the dispense, the slide was incubated at room temperature for 30 minutes in a parafilm-sealed Petri dish with Kimwipes saturated with 4X SSC. Then, the slide was transferred to a 100°C hotplate and dried for 30 minutes.

Our synthesis procedure depends on precise and specific localization of nucleoside triphosphates and enzymatic mixes to initiator spots, which we denote as features. Once these droplets are dispensed, however, they are prone to spread unevenly and uncontrollably across the glass surface and may contaminate neighboring features. To constrain the droplet, we sought to create virtual "wells" for each feature by increasing the hydrophobicity in the areas between features. Dispensed droplets should then stay localized on each feature. We first dispensed 0.3μL droplets containing 3X SCC and 1.5M Betaine on top of the features using a Mantis liquid handler and then dried the slide for 30 minutes on a 100°C hotplate. This creates an increased hydrophilic area surrounding each feature. To do this, the slide is dipped in Sigmacote (Sigma), which produces a neutral hydrophobic film over the areas of the glass which do not contain features, dried under a fume hood for 5 minutes, then dried for 5 minutes on a 100°C hotplate. Afterwards, the slide is washed twice with 0.2% SDS and three times with distilled water (Invitrogen UltraPure). We then stringently washed the slides by placing it in a boiling solution of 0.2X SSC for 15 minutes, then in room temperature distilled water (Invitrogen UltraPure). Lastly, to reduce Schiff bases and unreacted aldehydes, the slide was incubated for 10 minutes in a sodium borohydride reducing solution. The solution was prepared by dissolving 0.12g of $NaBH_4$ (Sigma) in 30mL phosphate buffered saline (PBS, Invitrogen), then adding 10mL of 100% ethanol. Afterwards, the slide was washed once with 0.2% SDS and three times with distilled water (Invitrogen UltraPure). The prepared slide is then kept in an ice-cold ethanol bath until use.

5.2 Synthesis, processing, and sequencing

We synthesized three replicates of the following three template sequences, each with 13 nucleotides: S01: 'ACTGATCGTAGCA'; S02: 'CTGATCACGTAGC', and S03: 'TAGCTGACGTCAT'. In total, we performed synthesis on nine total features.

Each synthesis cycle was composed of the following six steps: (i) the slide is placed on a custom jig for the Mantis liquid handler; (ii) a 0.5μL dispense of enzymatic reaction mix, comprised of 1x Custom Synthesis Buffer (14 mM Tris-Acetate, 35 mM Potassium Acetate pH 7.9, 7 mM Magnesium Acetate, 0.1% Triton X-100, 10% (w/v) PEG 8000) with 1U/μL TdT (Enzymatics) and 0.25mU/μL apyrase (NEB); (iii) a 0.1μL dispense of a nucleoside triphosphate at the

following 6X concentrations in 10% PEG 8000 + 0.05% Triton X-100: 60 µM dATP, 75µM Br-dCTP, 18µM dGTP, and 90µM dTTP; (iv) 30 second static incubation at room temperature; (v) four-step washing: once with 0.5X SSC + 0.01% Tween-20, once with 0.5X SSC and two times in distilled water (Invitrogen UltraPure); (vi) the slide placed back in the jig for the next cycle. For each synthesis cycle, the Mantis liquid handler performs four dispense cycles, described in (iii), one per nucleoside triphosphate. In each dispense cycle, a specific nucleoside triphosphate is deposited to all features for synthesis. Washes were performed by manually by transferring the slide between each of the defined solutions.

Following the last synthesis cycle, all strands from all features were ligated to a universal adapter. A thin, silicone-gasketed chamber (Grace Bio-Labs SecureSeal Hybridization Chamber) was adhered to the slide and a ligation mixture containing a universal adapter was flooded into the chamber. The ligation mixture is composed of 2.5µM 5App-rSBS9-dd adapter, 1X T4 DNA Ligase Buffer (NEB), 25% PEG 8000 (Sigma) and 1 unit of T4 RNA Ligase per µL (Enzymatics). Following ligation for 1 hour at room temperature, the chamber was removed and the slide washed once with 0.1% SDS and three times with distilled water (Invitrogen UltraPure).

We then released the synthesized strands from the slide surface by cleaving the uracils located on the 5' end of the initiators with USER enzymes. The cleavage reaction mixture was composed of 0.18 units of UDG (Enzymatics) per µL, 0.18 units of Endonuclease VIII (Enzymatics) per µL and 0.5µM ttSBS9 in USER TE-T buffer (40mM Tris-HCl pH 8.0, 1mM EDTA, 0.01% Tween-20). The cleavage mixture was dispensed as 2µL droplets with the Mantis liquid handler onto each of the features. The slide was then incubated for 1 hour at 37°C in a sealed chamber with a Kimwipe saturated with 0.1X SSC. Droplets containing the cleaved DNA strands were transferred by multichannel pipette, to a 96-well PCR plate where each well contained 5mM Tris-HCl pH 8.0 + 0.01% Tween-20.

Next, we generated a sequencing library for each feature. Using cycle-limited real-time PCR, 5µL of each feature was first amplified with the primers tSBS3 and ttSBS9, then with NEBNext Dual Indexing Primers for 15 cycles. Barcoded strands were then combined and sequenced single end using Illumina MiSeq v3 150.

5.3 Analyses and future improvements

Sequences from demultiplexed reads were first trimmed with cutadapt 1.9.1 [25], with an error tolerance up to 10%, to remove the 5' initiator oligo sequence (5Am12-fSBS3-ctgag) and the 3' universal oligo sequence (5App-rSBS9-dd adapter, **Supplementary Table 1**). Only reads containing both sequences for trimming were retained for further analysis.

Analyses of synthesis errors and diversity were performed as described above. We found perfectly synthesized strands, which had raw lengths ~50 bases, for each of the three tested template sequences across all three replicates (**Supplementary Fig. 32**). We then analyzed all synthesized strands and an *in silico* purified set with raw lengths between 39-52 bases, assuming an extension length of 3 to 4 bases per template nucleotide. The dominant mode of synthesis error was missing strand^{C} nucleotides which was reduced to observed experimental rates by size selection (**Supplementary Fig. 33**). Furthermore, we found that synthesized strands^{C} were diverse (**Supplementary Fig. 34**), indicating that our codec could be used to encode and retrieve data.

Subsequent iterations of this array-based synthesis platform will include both hardware and "wetware" improvements. In terms of hardware, a printhead containing multiple dispensers will improve parallelization. In addition, washing must be automated to make each cycle rapid and robust. For "wetware", further surface chemistry optimizations will mitigate potential issues with initiator and protein adsorption to the glass surface. Furthermore, process engineering of reaction conditions, such as heating, may improve mixing and denaturation of the DNA. Together, these improvements will increase synthesis speed, throughput, and accuracy.

6. Cost projections and cycle time estimation

6.1 Synthesizing DNA with an inkjet microarray printer

In order to compare reagent costs of enzymatic synthesis to that of chemical synthesis, we estimated the reagent costs for each, assuming that both processes can be implemented on the same device. For the device, we considered an inkjet microarray printer conceptually similar to that manufactured by Agilent Technologie s [26–28]. In such a microarray printer, each DNA sequence to be synthesized occupies a physical spot, also denoted as a feature, on a planar surface. Multiple sequences are arranged as a 2D array to allow spatial addressing (x and y Cartesian coordinates). All DNA sequences are synthesized in parallel per cycle, that is, all features receive their first nucleotide during the first cycle, they then all receive their second nucleotide during the next cycle, and so on. Each cycle consists of a series of reactions (**Supplementary Fig. 1**). Reagents for each reaction may be dispensed directly to each feature by non-contact inkjet dispense or to all features by first sealing the array surface to form a flow cell and then flushing the reagent through. The reagent to be dispensed by inkjet is denoted as droplet whereas the reagent to be flushed is denoted as flowcell.

6.2 Reagent costs

We obtained the volumetric cost of each flowcell or droplet reagent. **Supplementary Table 6** lists the reaction steps of standard phosphoramidite chemistry and enzymatic biochemistry presented in this study. Reactions are tagged by type (droplet *or* flowcell) and retail price per milliliter as of September 2017. Total droplet volumes (V_d) and flowcell volumes (V_f) of each reagent per cycle are also detailed.

We note that the enzymes may be used over multiple cycles since TdT:apyrase does not get inactivated after a synthesis reaction. We have used TdT:apyrase for at least ten consecutive cycles of extension (**Supplementary Fig. 7**) with no observable deterioration in performance. As such, it is possible for enzyme costs per cycle (**Supplementary Table 6**) to be readily reduced by 10-fold. Furthermore, we obtained a quote for bulk pricing of TdT, reducing its price to 71% of the listed price. Factoring in both price reductions, $\$_{de}$ can be reduced ~14-fold, from 61.3 USD per milliliter to 4.38 USD per milliliter.

The total cost of reagents for a cycle of each synthesis process can be computed as follows:

$$\text{Cycle\_cost}_{\text{enz}} = (\$_{fe} \times V_{\text{f}}) + (n \times \$_{de} \times V_{\text{d}}) \quad (35)$$

$$\text{Cycle\_cost}_{\text{chem}} = (\$_{fc} \times V_{\text{f}}) + (n \times \$_{dc} \times V_{\text{d}}) \quad (36)$$

where $\$_{fe}$ represents the cost of flowcell reagents in enzymatic synthesis, V_{f} is the flowcell volume in milliliters, n is the total number of features, $\$_{de}$ is a constant representing the cost of droplet reagents in enzymatic synthesis, V_{d} is the droplet volume in cubic centimeters, $\$_{\text{fc}}$ represents the cost of flowcell reagents in chemical synthesis, and $\$_{\text{dc}}$ represents the cost of droplet reagents in enzymatic synthesis.

Flowcell volume (V_f), can be expressed as in relation to height ($c_1 = 0.1$cm, assuming a constant flowcell height [27] and flowcell area (A):

$$V_{\text{f}} = c_1 \times A \quad (37)$$

Furthermore, droplet volume (V_d), assuming the droplet forms a half sphere on the surface, can be expressed as a function of its feature size diameter (*d*) and the constant $c_2 = \pi \div 12$:

$$V_{\text{d}} = c_2 \times d^3 \quad (38)$$

Flowcell area (A) can be expressed as a function of number of features (*n*) and density (*D*) of features:

$$\text{A} = \text{n} \div \text{D} \quad (39)$$

6.2.1 Reagent cost per cycle

Based on equations 37, 38, and 39, equations 35 and 36 can be reformulated as:

$$\text{Cycle\_cost}_{\text{enz}} = (\$_{fe} \times c_1 \times n \div D) + (n \times \$_{de} \times c_2 \times d^3) \quad (40)$$

$$\text{Cycle\_cost}_{\text{chem}} = (\$_{fc} \times c_1 \times n \div D) + (n \times \$_{dc} \times c_2 \times d^3) \quad (41)$$

We then utilized the number of features and feature density from the Agilent SurePrint G3 system as a physical basis for projecting reagent costs for synthesis. For this system, the maximum number of features is approximately 1 million ($n = 1{,}000{,}000$) with a density of ~71,000 spots per square centimeter (D = 71,000), obtained by estimating a surface area of 14cm^2 ($A = 14$) out of a microscope slide with a total surface area of 18.75cm^2 [29]. Furthermore

the feature size can be approximated to be 15-38 microns ($d = 0.0015\ to\ 0.0034$), based on a dispense volume of 1-10 picoliters [30] and equation 38.

With these set number of features and density, we projected the reagent cost per cycle for both enzymatic and phosphoramidite as a function of miniaturizing feature sizes, (**Supplementary Fig. 35A**). With smaller feature sizes, we found that the reagent cost per cycle for both processes drops to approximately the price of the flowcell reagent, indicating that the droplet reagent cost for all 1 million features are negligible. For enzymatic, this floor occurs when feature sizes are below 1-5 microns, depending on the enzyme droplet price ($\$_{de}$) considered whereas for phosphoramidite, the floor occurs for feature sizes below ~34 microns. For all feature sizes less than 1 micron, the enzymatic cost per cycle will be >1,000-fold cheaper than phosphoramidite cost per cycle. Considering current feature sizes of ~15-34 microns, we found that the reagent cost per cycle for enzymatic could already be cheaper than phosphoramidite. For example, with 15 micron features, phosphoramidite reagent cost per cycle is 0.626 USD whereas the enzymatic reagent cost per cycle is 0.055 USD (assuming$\$_{de}$= 61.3) or 0.0044 USD (assuming $\$_{de}$= 4.38), a ~11-fold and ~140-fold drop in cost respectively.

6.2.2 Reagent cost per megabyte

We next sought to project the reagent cost for synthesizing sufficient quantity of DNA to encode a megabyte of data. The previous analysis of reagent cost per cycle indicated that as feature size is miniaturized, the flowcell reagent becomes the dominant cost rather than the droplet reagent (**Supplementary Fig. 35A**). Therefore, a cost-effective strategy would be to increase the number of synthesized features, n, for a given surface area (increasing the feature density, D, as a result) per cycle and to minimize the total number of cycles, thereby limiting flowcell reagent cost. For this approach, we assume that features are maximally packed, end-to-end, in a given surface area. The flowcell area (A) can be alternatively expressed as a function of the number of features (n) and feature size diameter (d):

$$A = n \times d^2 \tag{42}$$

With equation 42, the cost per cycle equations 40 and 41 can be reformulated as:

$$\text{Cycle\_cost}_{\text{enz}} = A \times (\$_{fe} \times c_1 + \$_{de} \times c_2 \times d) \tag{43}$$

$$\text{Cycle\_cost}_{\text{chem}} = A \times (\$_{fc} \times c_1 + \$_{dc} \times c_2 \times d) \tag{44}$$

To store a megabyte of data in DNA, the number of cycles and number of features must be determined. Since the number of cycles should be minimized to limit flowcell reagent cost, template sequences should be as short as possible, which results in data being spread across a large number of features. As a result, most of the nucleotides for each template sequence should be allocated for addressing. Assuming an average efficiency rate of storage of 1 bit per template nucleotide, 2^{20} (1,048,576) sequences must be synthesized and each sequence must contain 28 template nucleotides (20 for addressing and 8 for data) to store 1 megabyte of data. The cost for a template sequence of 28 nucleotides requires 28 cycles worth of reagents. As the feature size (d) is decreased, however, the number of features (n) increases for a given area (A), and can be derived from equation 42 as:

$$n = A \div d^2 \quad (45)$$

With equations 43, 44, 45, we computed the reagent cost per megabyte assuming a maximum number of features packed into a 14 cm^2 area ($A = 14$), 28 cycles, and that the efficiency rate of storage is 1 bit per template nucleotide which requires 2^{20} features ($n = 1{,}048{,}576$) for storing each megabyte (**Supplementary Fig. 35B**). Similar to our previous cost per cycle analyses, these calculations show that reagent costs per megabyte are cheaper than phosphoramidite for current feature sizes of 15-34 microns. These projections show that the reagent cost per megabyte could, in theory, be reduced by a maximum of ~11 orders of magnitude for enzymatic compared to a maximum of ~8 orders of magnitude for phosphoramidite, if the feature size was the diameter of double-stranded DNA ($d = 2.7e\text{-}7$). The reagent cost per megabyte can be equivalent to that of magnetic tape (2E-5 USD per megabyte, derived from 2E-2 USD per gigabyte [31]) if feature sizes (d) are ~40nm for phosphoramidite or ~350-800nm ($\$_{de}$= 61.3 or 4.38) for enzymatic. This would correspond to ~7 orders of magnitude cost drop from the calculated reagent price of ~18 USD per megabyte when synthesized on a system with feature number and density parameters similar to the Agilent SurePrint G3.

6.2.3 Practical considerations

These models project theoretical costs and will be altered depending on practical implementations. The three most important factors to consider are as follows:

1. **Efficiency rate of storage:** For ease, we set the average efficiency rate of storage for both enzymatic and phosphoramidite to be equivalent, storing an average of 1 bit per

template nucleotide. The rate for each approach may be different depending on factors such as synthesis accuracy and the required addition of error-correction codes per template sequence to ensure accurate information recovery. Altering the efficiency rate of storage for each processes will change costs linearly, and the resulting difference between enzymatic and phosphoramidite approaches would likely be within an order of magnitude. Improvements to enzymatic synthesis will increase the efficiency rate of storage to be competitive to that of phosphoramidite synthesis. Such improvements will also influence the number of diversely synthesized needed for template reconstruction and inform the minimum required feature size.

2. **Feature density:** For our reagent cost per megabyte projections, features are maximally packed with no spacing between. Practically, features are likely to be separated by a gap, usually a fraction of the feature size, to accommodate for potential positioning errors when droplets are dispensed. The number of features will then decrease inversely proportional to the square of the gap size (equation 45 to be modified accordingly). As this parameter is the same for calculating reagent costs for both phosphoramidite and enzymatic synthesis, altering the number of features may change absolute costs for each approach but relative comparisons between approaches will remain unchanged.
3. **Feature size:** Reaching the projected costs depends on overcoming significant engineering challenges associated with miniaturizing feature sizes. Current inkjet printheads dispense 1-10 picoliter droplets, resulting in feature sizes of 15-38 microns (equation 4 and [30]). To reach the projected cost per megabyte equivalent to magnetic tape, phosphoramidite features must be ~40nm which requires dispensing a 0.016 attoliter droplet, whereas enzymatic features must be ~350-800nm which requires dispensing a droplet of 11-134 attoliters. While it is now possible to dispense a droplet of hundreds of attolitres [32], no sub-attoliter dispensers are available to our knowledge. To achieve this cost, significant technology development and engineering will be required. Development of alternative systems that consume equivalent reagent quantities, perhaps requiring modifications to the enzymatic process, are warranted.

6.3 Equipment costs

We assume an equivalent equipment cost between enzymatic and phosphoramidite DNA synthesis. Currently, our reactions occur under ambient conditions without a need for stringent control of temperature or oxygenation. Thus, we reasoned that our reagents could be used directly in a machine designed for phosphoramidite chemistry such as the Agilent SurePrint G3.

Equipment amortization is another important, but often neglected, cost consideration. Capital equipment costs are likely to increase significantly as DNA synthesis is scaled to achieve target costs. To miniaturize feature sizes, specialty dispensers will be required. As the number of these features increases, the time required for a dispenser to find the correct feature to receive a droplet reagent, the dispenser seek time, becomes an important consideration. Even if multiple dispensers are combined into a large printhead to reduce seek times, positioning systems likely with nanometer-scale resolution will be required, which may be expensive or prone to breakdown. While these are all important factors, we lack sufficient information to estimate relevant parameters. Accordingly, we assumed for ease that all seek times could be instantaneous and thus equipment amortization for enzymatic and phosphoramidite would be primarily dependent on their respective cycle time. Our conservative estimate of enzymatic cycle time is ~4-fold shorter than phosphoramidite chemistry (**Supplementary Table 6**), which could result in a shortened amortization schedule, further reducing total synthesis costs.

Supplementary Figures

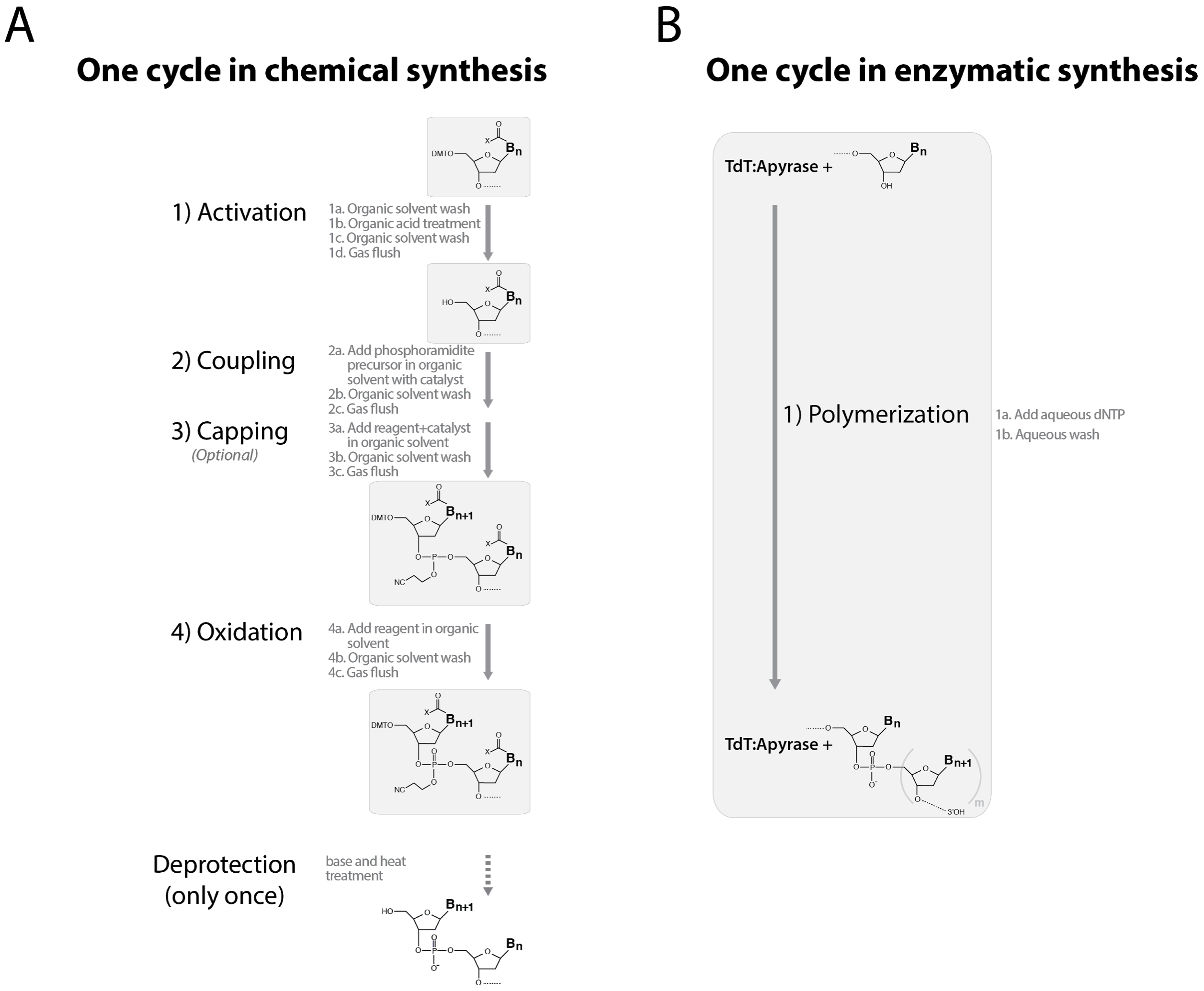

Supplementary Figure 1. A single cycle of phosphoramidite chemistry versus this enzymatic synthesis strategy.

The number of steps required for a single cycle of synthesis using phosphoramidite chemistry and using this enzymatic DNA synthesis strategy. For enzymatic synthesis, a wash step is needed to clean up reaction byproducts or for downstream processing, but it is not required at every cycle.

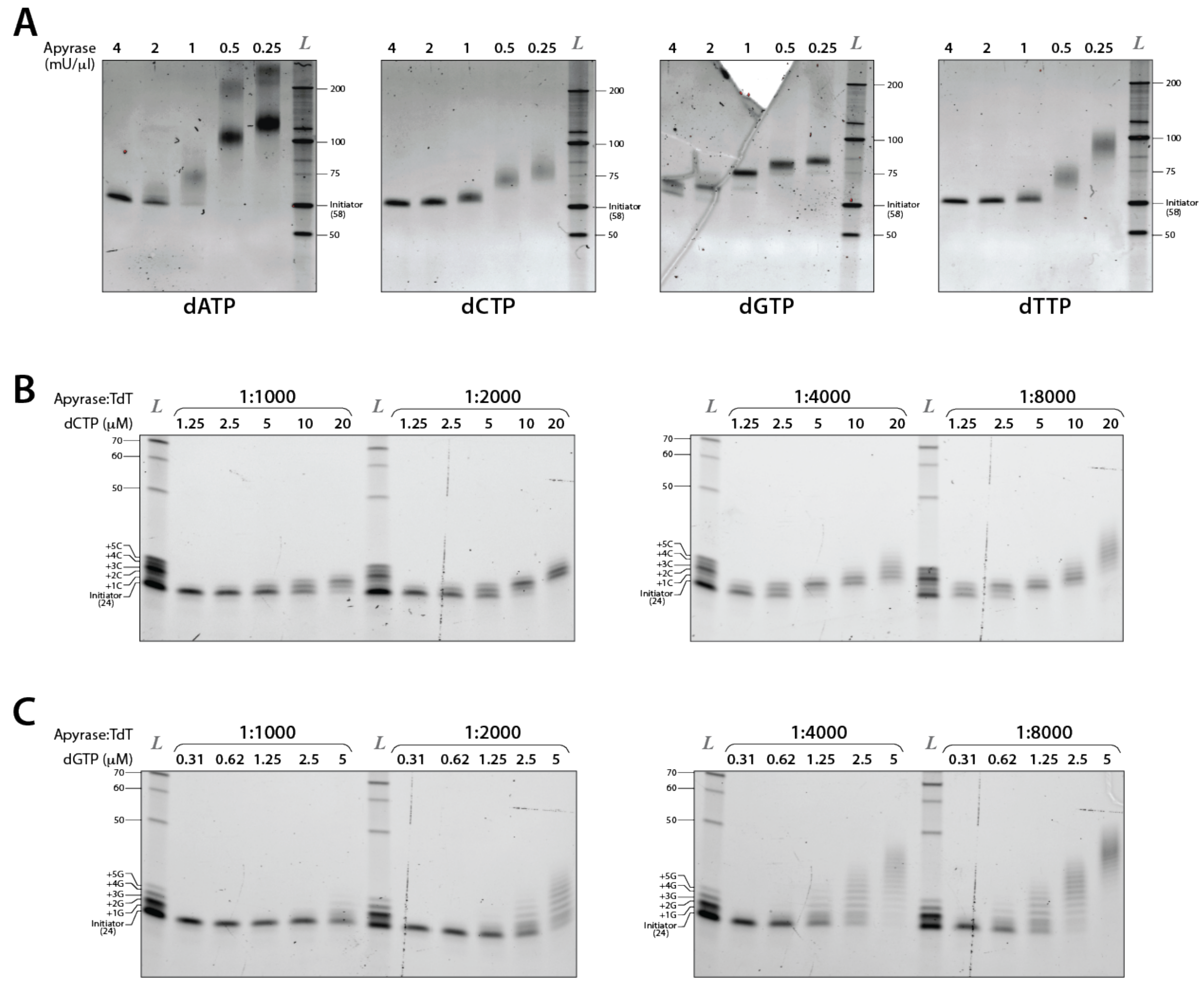

Supplementary Figure 2. Optimizing and tuning of TdT:apyrase ratio.

(A) Initiator extension with dATP, dCTP, dGTP or dTTP by four different TdT:apyrase ratios. TdT concentration is constant at 1U/µL, apyrase concentration varies and is marked above each lane. mU is milliunits. Gels are 15% TBE-urea. "*L*" is ssDNA size marker. See **Supplementary Note** for reaction details. (B,C) Extension of an initiator with various concentration of dCTP (B) and dGTP (C) with 1:1000, 1:2000, 1:4000, or 1:8000 apyrase:TdT ratio. Apyrase:TdT ratio, as well as dNTP concentrations are marked above each lane. Gels are 15% TBE-urea. "*L*" is ssDNA size marker and includes the unextended initiator, as well as initiator synthesized with 1, 2, 3, 4, or 5 additional Cytosines (B) or Guanines (C). See **Supplementary Note 1** for reaction details.

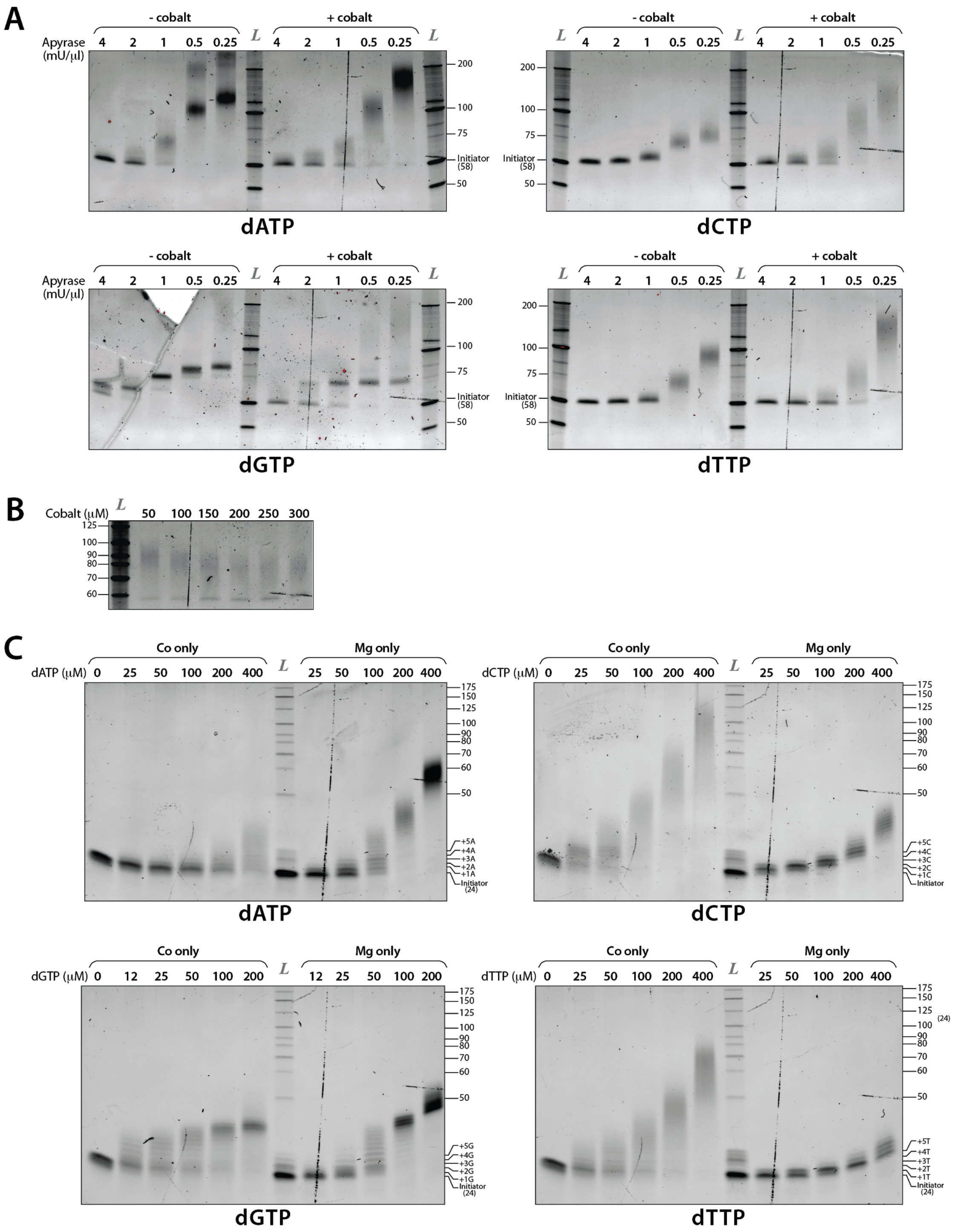

Supplementary Figure 3. Effect of cobalt on TdT:apyrase performance.

(A) Initiator extension with each dNTP with various ratios of TdT:apyrase in the presence of magnesium and absence or presence (250μM) of supplemental cobalt. TdT concentration is constant at 1U/μL with varying apyrase concentration marked above each lane. Gels are 15% TBE-urea. "*L*" is ssDNA size marker. (B) Initiator extension with 300μM dATP in presence of

Magnesium and increasing amounts of supplemental cobalt. Cobalt concentrations are marked above each lane. Gel is 15% TBE-urea. "*L*" is ssDNA size marker. (C) Initiator extension with each dNTP by TdT:apyrase in magnesium-only or cobalt-only reactions. dNTP concentration is marked above each lane. Gel is 15% TBE-urea. "*L*" is ssDNA size marker and includes the unextended initiator, as well as initiator synthesized with 1, 2, 3, 4, or 5 additional nucleotides of the corresponding base, that is Cytosines for the gel with cytosine extension. See **Supplementary Note 1** for reaction details.

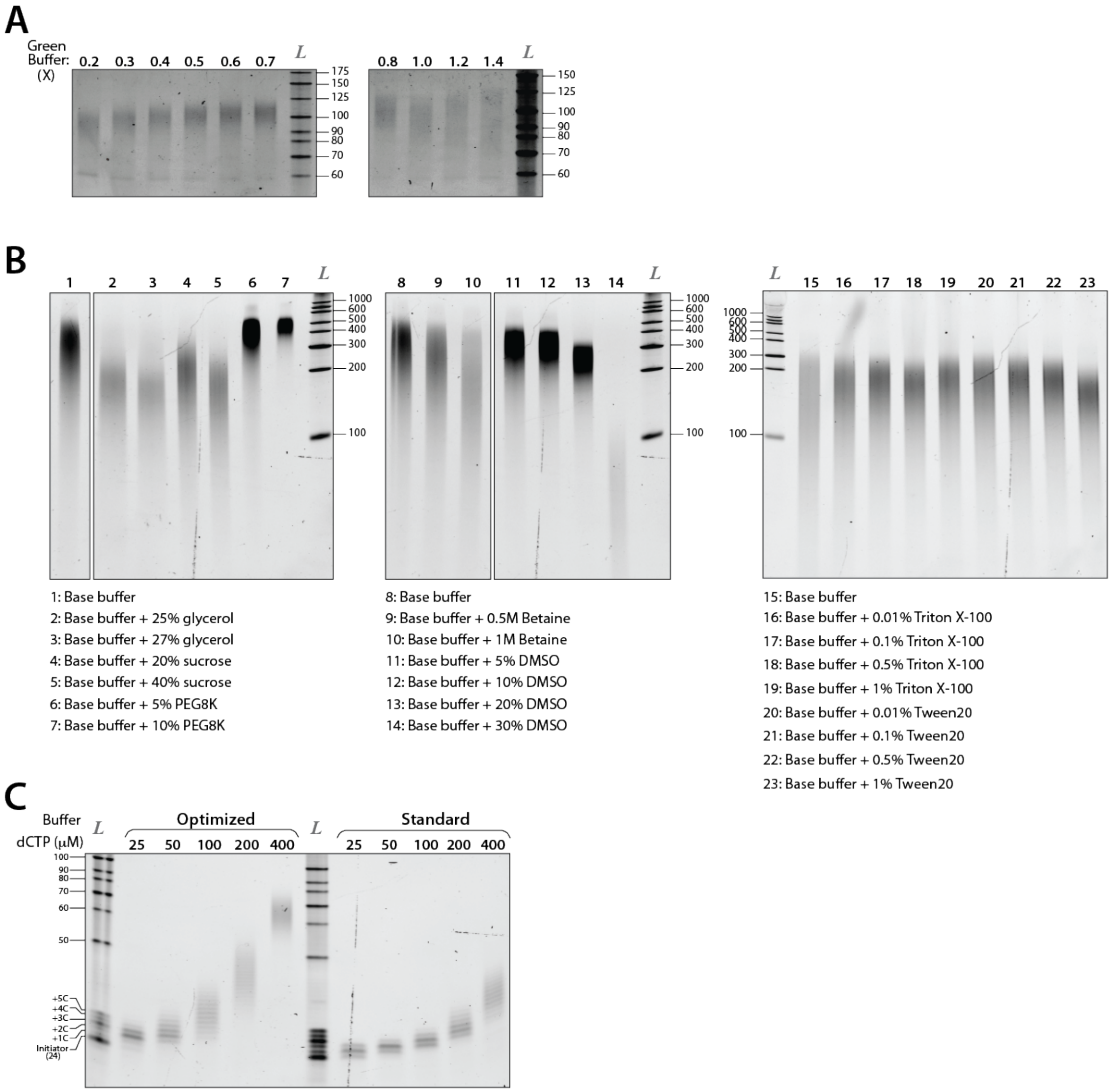

Supplementary Figure 4. Buffer and additives optimization for TdT:apyrase.

(A) Initiator extension with dATP by TdT:apyrase with increasing concentration of Enzymatics Green Buffer. Final buffer concentration is marked above each lane. Gels are 15% TBE-urea. "*L*" is ssDNA size marker. (B) Initiator extension with a 500μM mixture of all dNTPs by TdT:apyrase in presence of various additives in different concentrations. Each lane is labelled with a number, the additive and its concentration in that lane are listed below the gels. Gels are 10% TBE-urea. "*L*" is an RNA size marker. (C) Initiator extension with various dCTP concentration by TdT:apyrase in our optimized buffer and the standard buffer. Gels are 15% TBE-urea. "*L*" is ssDNA size marker and includes the unextended initiator, as well as initiator

synthesized with 1, 2, 3, 4, or 5 additional Cytosines. See **Supplementary Note 1** for reaction details.

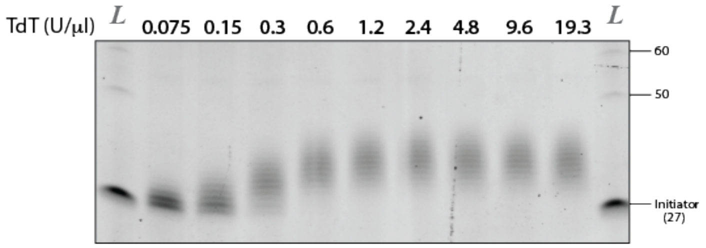

Supplementary Figure 5. Optimizing TdT to initiator ratio.

Initiator extension with dATP by TdT:apyrase with increasing concentration of TdT. Values above each lane mark the concentration of TdT at units per µl. Apyrase concentration is constant at 1mU/µL. Gel is 15% TBE-urea. "*L*" is ssDNA size marker and includes the unextended initiator which is 27 bases long. See **Supplementary Note 1** for reaction details.

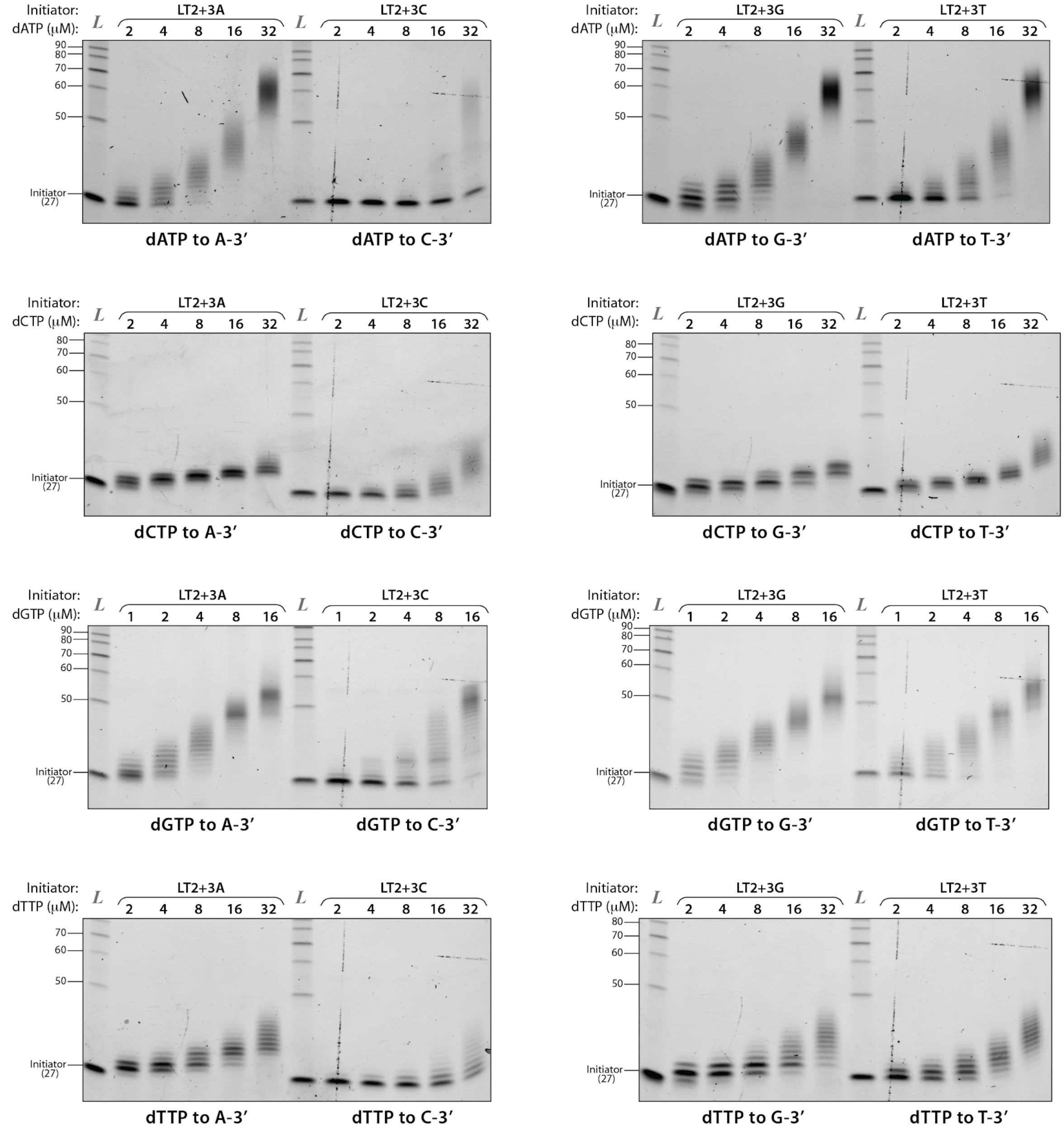

Supplementary Figure 6. Optimal nucleotide concentrations based on 3’ nucleotide to be extended.

TdT:apyrase performance and nucleotide concentration optimization for all sixteen possible combinations of 3’ base of the initiator and the incoming nucleoside triphosphate (4 by 4). Each combination is evaluated on five lanes. The concentration of the relevant nucleotide is shown in

μM on top of each lane. Gels are 15% TBE-urea. "*L*" is ssDNA size marker and includes the unextended initiator which is 27 bases long. See **Supplementary Note 1** for reaction details.

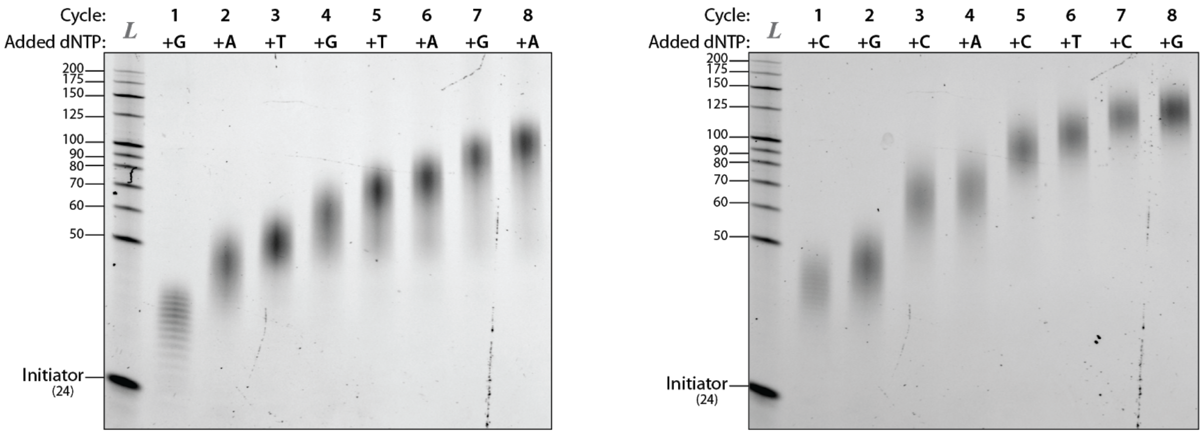

Supplementary Figure 7. TdT:apyrase activity over multiple cycles.

Multiple consecutive rounds of extension using the TdT:apyrase reagent. Eight of ten synthesis cycles are shown. Synthesis of two different template sequences are shown. The nucleoside triphosphate added in each cycle is indicated on top of each lane. The gel on the left is identical to that shown in **Fig. 1B**. All samples that are shown on each gel were aliquots of the same reaction that were samples after the addition of each nucleoside triphosphate. Gels are 15% TBE-urea. "*L*" is ssDNA size marker and includes the unextended initiator which is 24 bases long. See **Supplementary Note 1** for reaction details.

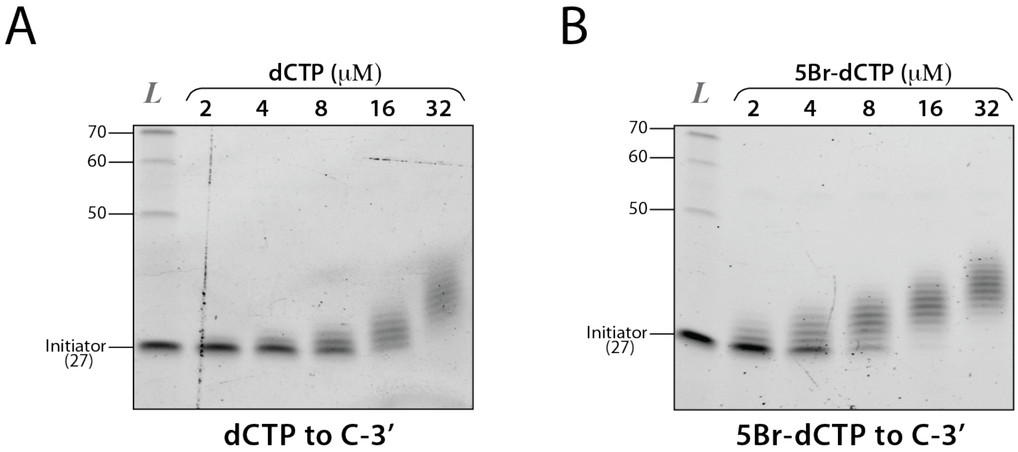

Supplementary Figure 8. Evaluation of 5-Bromo-dCTP as a substitute for natural dCTP.

Evaluation of 5-Bromo-dCTP and natural dCTP for TdT:apyrase. "*L*" is ssDNA size marker and includes the initiator oligonucleotide which is 27 bases long and ends in three cytosines. Extension lengths were evaluated over indicated concentration of (A) natural dCTP and (B) 5-Bromo-dCTP (5Br-dCTP). See **Supplementary Note 1** for reaction details.

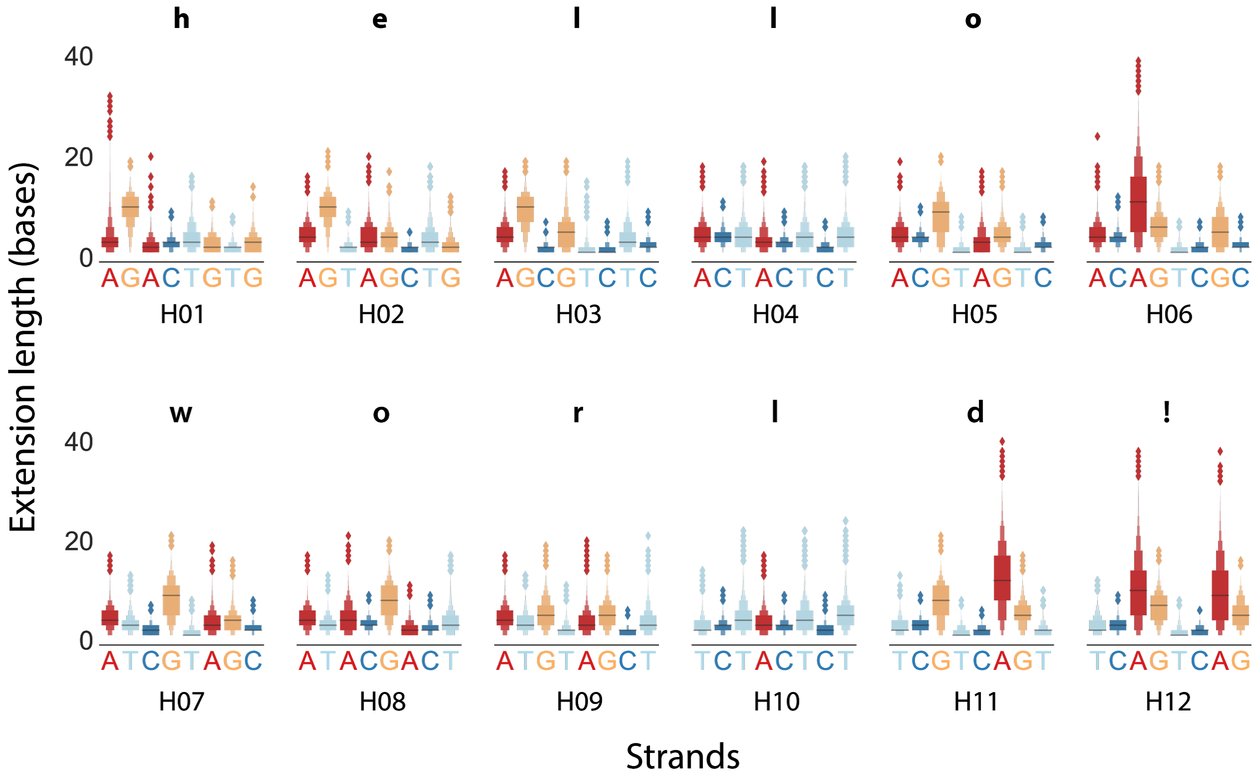

Supplementary Figure 9. Extension lengths for perfect strands of H01-H12.

Extension lengths for each nucleotide from perfect strands are displayed as a letter-value plot for each template sequence. Source data are provided as a Source Data file.

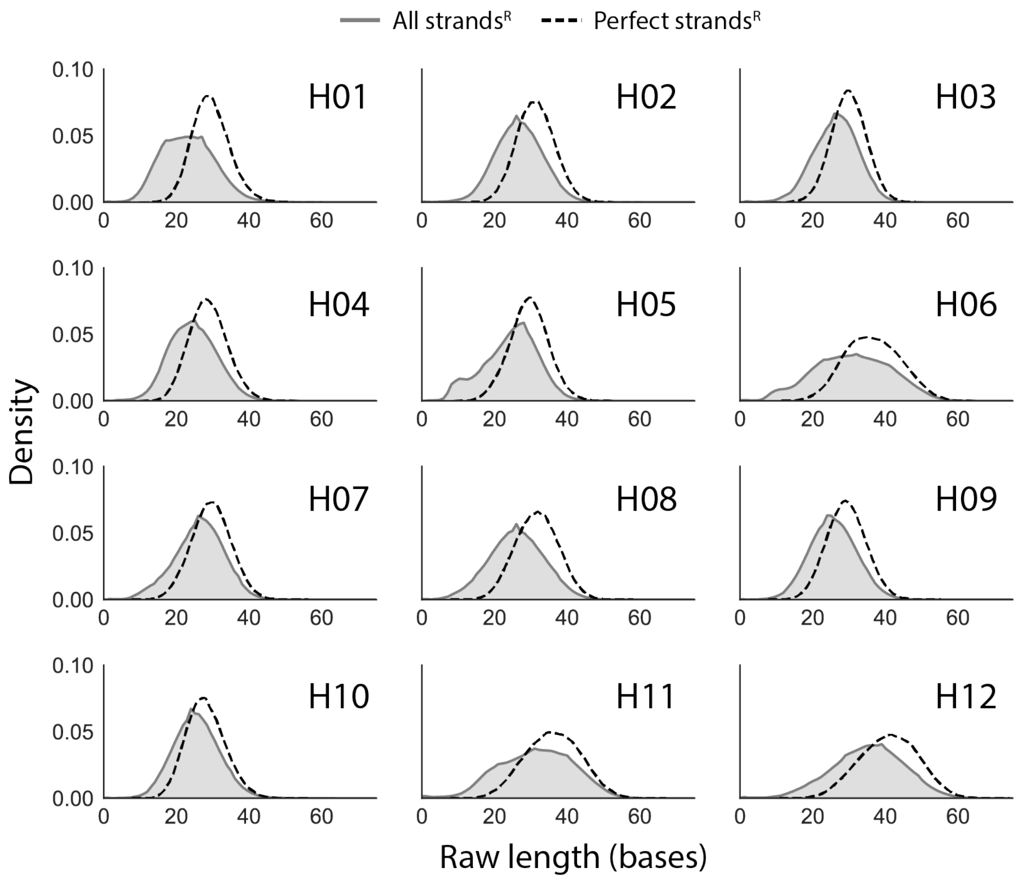

Supplementary Figure 10. Raw lengths for all and perfect strands of H01-H12.

All synthesized strands^R of H01-H12 were sequenced with Illumina. (A) Length distribution for the set of all (gray shading) and perfectly (dashed line) synthesized and sequenced raw strands are shown. Distributions are derived via kernel density estimation. The number of all strands to perfect strands for each template sequence are as follows: H01 {all: 396649, perfect: 42337}; H02 {all: 429497, perfect: 62243}; H03 {all: 607501, perfect: 89302}; H04 {all: 902014, perfect: 200154}; H05 {all: 763647, perfect: 115345}; H06 {all: 632786, perfect: 126849}; H07 {all: 630388, perfect: 169767}; H08 {all: 705227, perfect: 113567}; H09 {all: 1006930, perfect: 207182}; H10 {all: 1174726, perfect: 406172}; H11 {all: 540587, perfect: 105730}; H12 {all: 510056, perfect: 68233}. Source data are provided as a Source Data file.

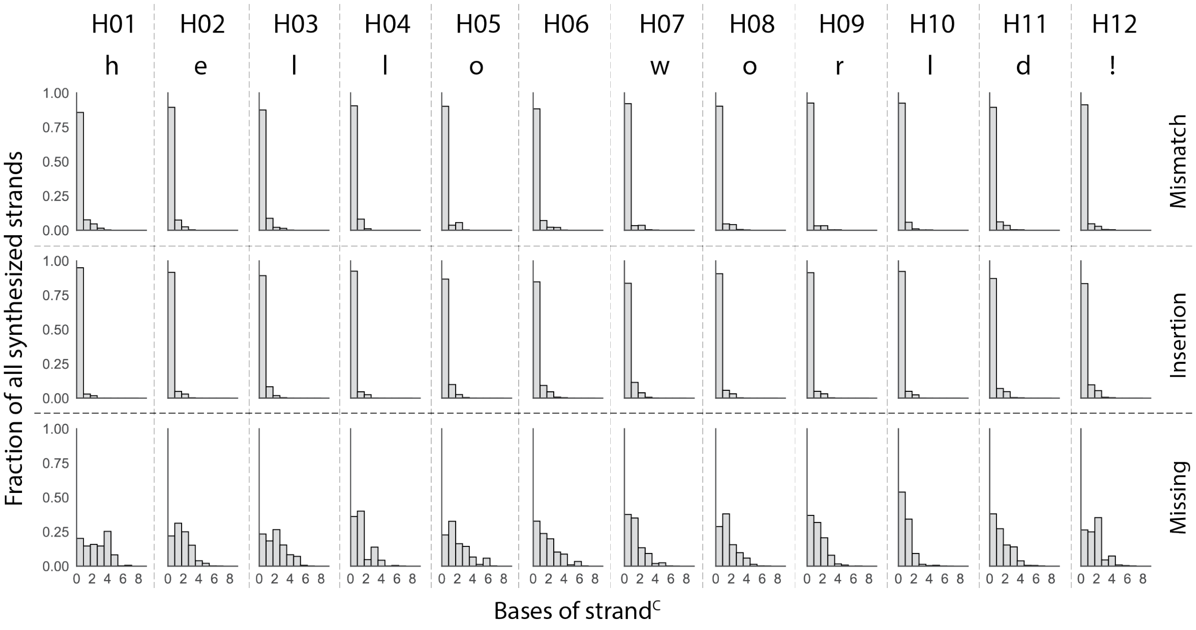

Supplementary Figure 11. Synthesis error analysis for all strands of H01-H12.

All synthesized strands^R were sequenced with Illumina and transitions of non-identical nucleotides were extracted to form strands^C. Each of these strands^C is aligned, by Needleman-Wunsch, to its respective template sequence. For each alignment, the number of mismatches, insertions, and missing nucleotides are tabulated.

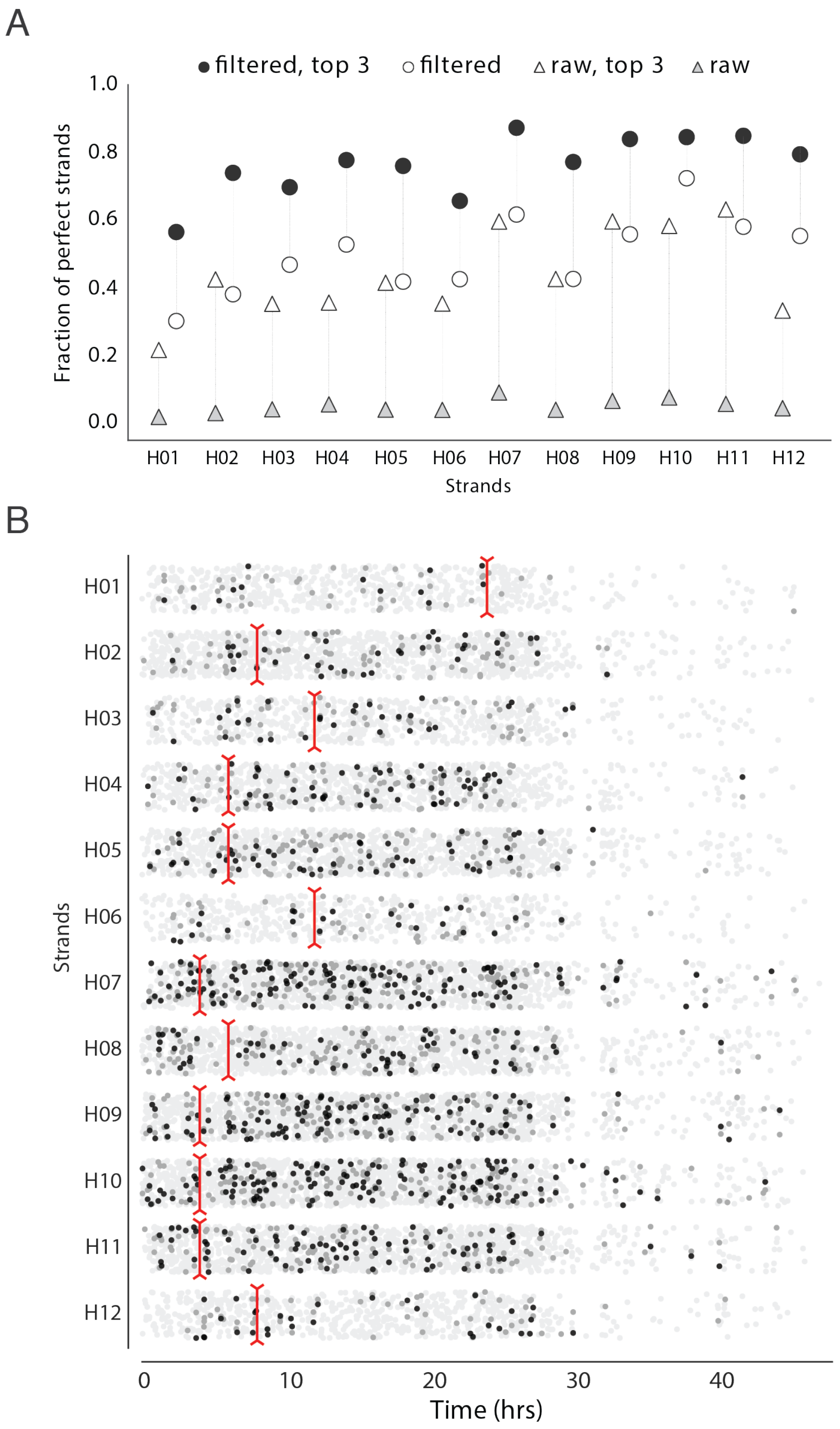

Supplementary Figure 12. Nanopore sequencing and decoding of H01-H12.

Nanopore sequencing (Oxford) of synthesized raw strands. For each raw strand, the sequence of non-identical nucleotides are extracted to form compressed strands (strands^C). Fraction of perfect strands^C are plotted out of the set of all strands^C (filled triangles) or out of the set of the top 3

most abundant strands^{C} (open triangles). Strands^{C} can be filtered based on the design of the template sequence (**Methods**). Perfect strands^{C} appear at a higher fraction out of the set of strands passing this filter (open circles) or out of the set of the top 3 most abundant filtered strands^{C} (filled circles). (B) Sequencing stream of raw strands from a nanopore array (Oxford). Reads which pass the data retrieval filter - expected number of strand^{C} nucleotides with a terminal ‘C’ (filled black), those with only the expected number of strand^{C} nucleotides (filled dark gray), and remainder of reads (light gray) are plotted according to their time stamp. For each strand, the time corresponding to correct data retrieval with a 99.9% probability from the collected sequences up to that point is marked (vertical red line).

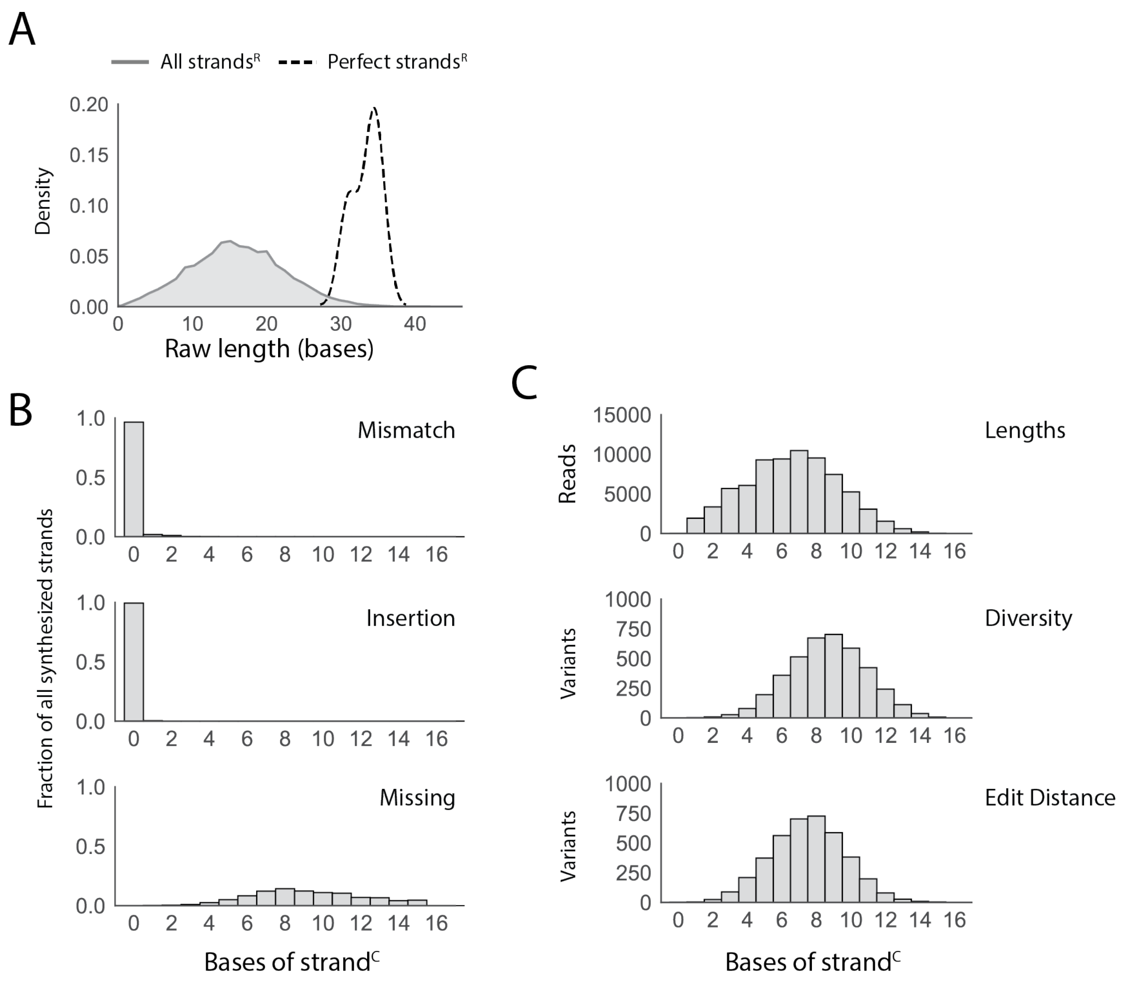

Supplementary Figure 13. Synthesis error analyses and diversity of all synthesized strands of E0.

All synthesized strands^{R} of E0 were sequenced with Illumina and transitions of non-identical nucleotides were extracted to form strands^{C}. (A) Length distribution for the set of all (gray shading) and perfectly (dashed line) synthesized and sequenced raw strands are shown. Distributions are derived via kernel density estimation. The number of all strands to perfect strands for the template sequence is as follows: E0 {all: 77536, perfect: 3}. For each raw strand, a sequence of non-identical nucleotides were extracted to form strand^{C}, which is then aligned, by Needleman-Wunsch, to its respective template sequence. (B) For each alignment, the number of mismatches, insertions, and missing nucleotides from strand^{C} are tabulated. (C) Number of sequencing reads at each length (number of nucleotides of strand^{C}) is tabulated. Diversity is evaluated as the number of unique variants at each strand^{C} length and the Levenshtein edit

distance is computed between each strand^C and the E0 template sequence. Strands^C were filtered for read counts of at least 2 to remove aberrantly synthesized or sequenced variants.

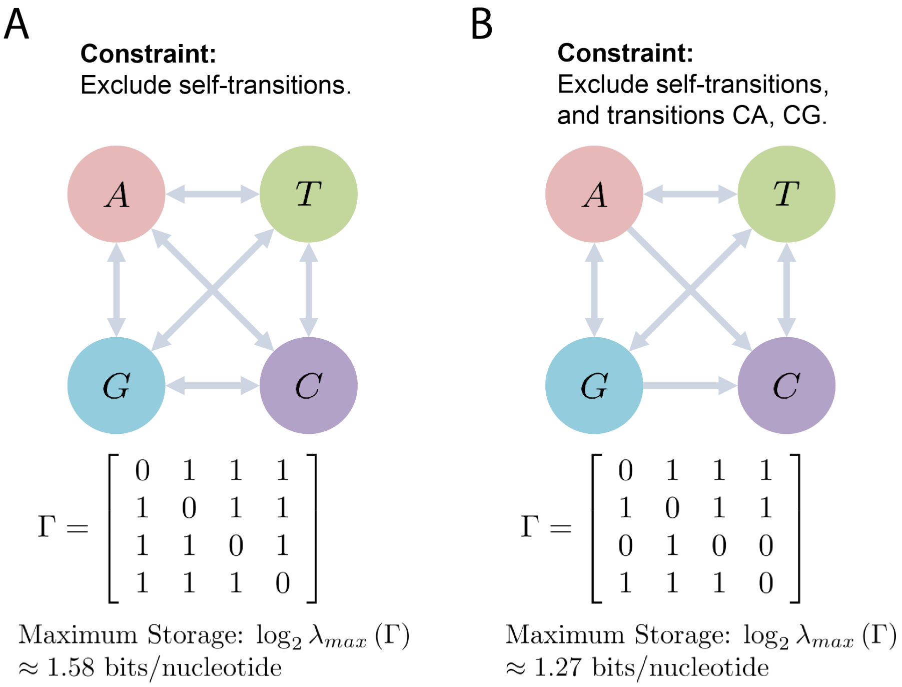

Supplementary Figure 14. Constraints for valid transitions between nucleotides.
As physical processes, both chemical synthesis and enzymatic synthesis have constraints for valid transitions between nucleotides. A transition matrix with no self-transitions (A) and a transition matrix excluding specific transitions (B) are depicted. Based on whether certain transitions are permitted, there exists a fundamental limit for the maximum number of bits per nucleotide that is possible to store. This limit is equal to $log_2\, \lambda_{\max}(\Gamma)$ where Γ is the transition matrix indicating valid transitions to write. The notation $\lambda_{\max}(\Gamma)$ indicates the maximum eigenvalue of Γ.

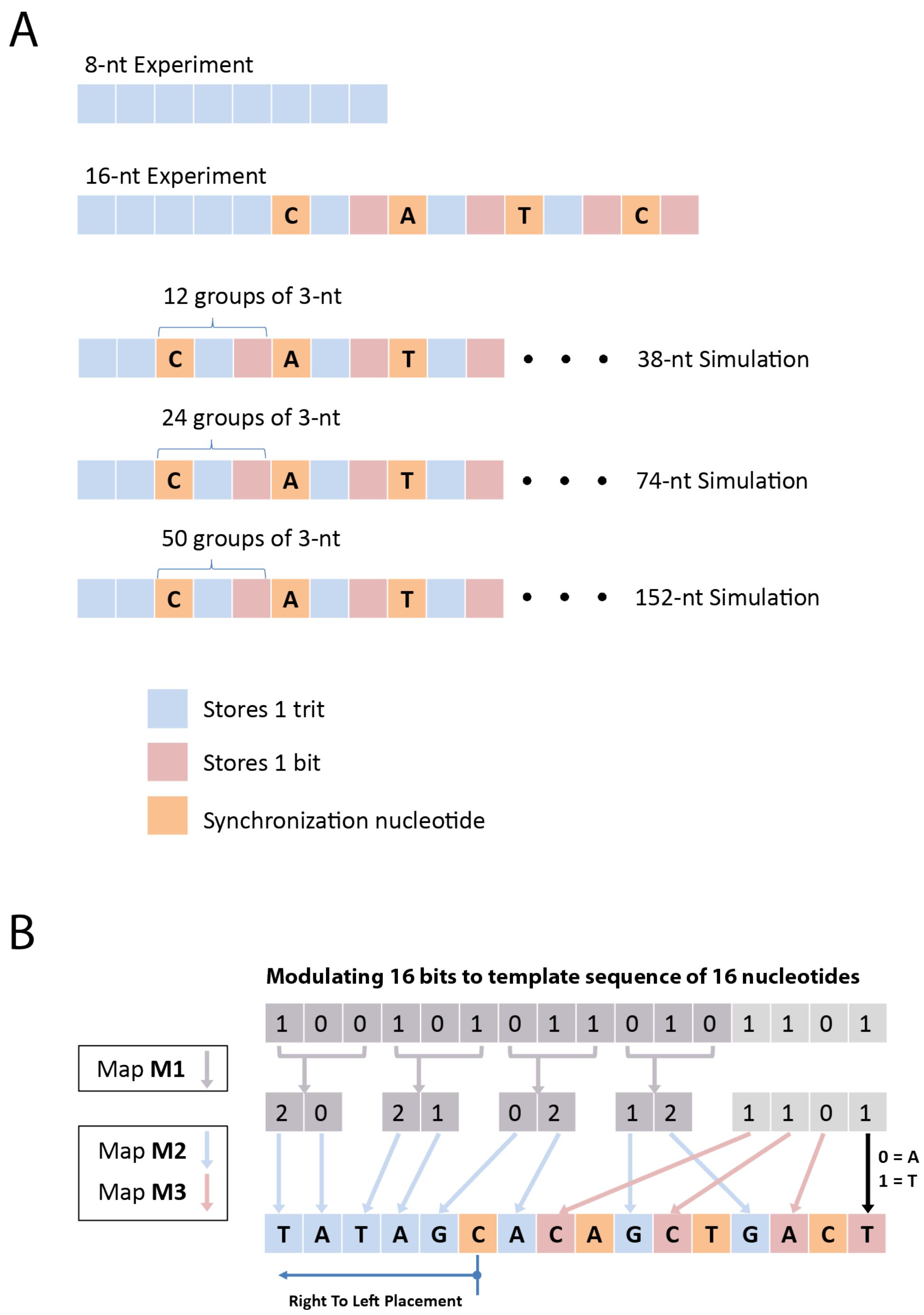

Supplementary Figure 15. Placement and modulation of information into template sequences.

(A) The placement of information within a template sequence for both experimental and simulated storage systems. For experimental systems, template sequences contained 8 or 16 nucleotides each. For simulated systems, template sequences contained 38, 74, or 152

nucleotides each. Each nucleotide in a template sequence either stores 1 trit (blue), 1 bit (red), or is allocated for synchronization (orange). (B) A modulation scheme to map 16 bits to a sequence of 16 nucleotides. As an intermediate step, 16 bits are converted to a mixture of 8-trits and 4-bits using map M1 (**Supplementary Table 9**). Subsequently, given the prior placement of synchronization nucleotides, 8-trits are converted to nucleotides (light blue arrows) using map M2 (**Supplementary Table 9**). For the first 5 trits, the placement of nucleotides begins with the first synchronization nucleotide ‘C’, and occurs from right-to-left order. This initial ad-hoc placement ensures non-identical transitions between nucleotides, compared to a left-to-right order starting from an initiator nucleotide which may conflict when transitioning to a synchronization nucleotide. The remaining 4-bits are converted to nucleotides (red arrows) via map M3 (**Supplementary Table 9**) with the exception of the final bit which is converted with an ad-hoc mapping (black arrow). Demodulation from nucleotides to bits reverses these steps using maps M1, M2, and M3 (**Supplementary Table 9**). Demodulation assumes the existence of correctly placed synchronization nucleotides.

A

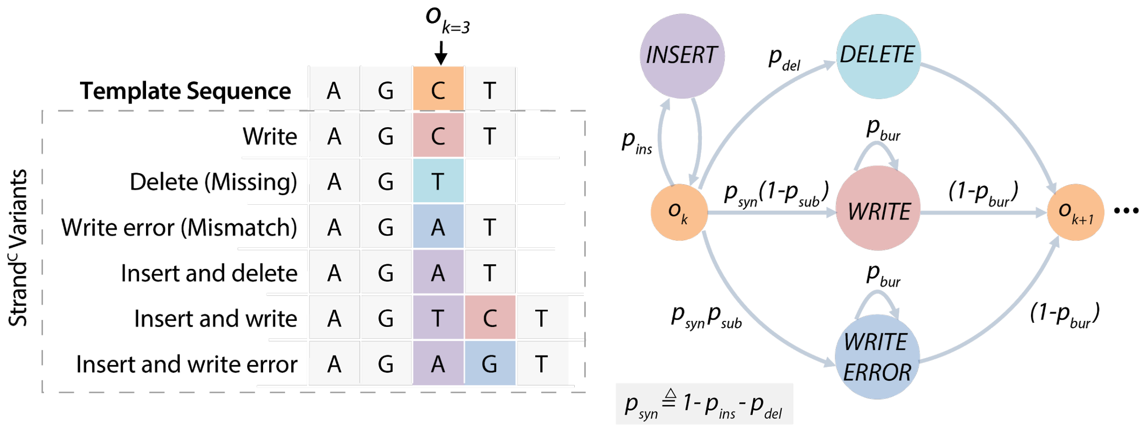

B

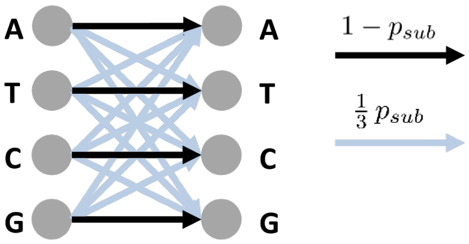

$$x, y \in \{A, T, C, G\}$$

$$\phi(x,y) \triangleq \left\{ \begin{array}{ll} 1 - p_{sub}, & \text{if } x = y \\ \frac{1}{3}\, p_{sub}, & \text{if } x \neq y \end{array} \right\}$$

Supplementary Figure 16. Markov model for the production of DNA strands.

(A) A Markov model provides a statistical framework for the production of DNA strands^{C} created from a desired template sequence. At the k-th state denoted by o_k, the Markov model specifies the process for writing the k-th nucleotide in the template sequence. An example is provided for the template sequence (AGCT). The process for writing the third nucleotide at position $k = 3$ could lead to several strand^{C} variants, each with a different probability of occurrence. The Markov model contains states which include a deletion error (missing strand^{C} nucleotide) with probability p_{del}, and an insertion error with probability p_{ins}. The probability of synthesis $p_{\text{syn}} \triangleq 1 - p_{\text{ins}} - p_{\text{del}}$ denotes the probability for the event of either a correct write, or

a write error (mismatch or substituted strand^{C} nucleotide). (B) In the event of synthesis of a strand^{C} nucleotide, either a correct write occurs with probability $(1 - p_{\mathrm{sub}})$, or a write error (mismatch or substituted strand^{C} nucleotide) occurs with total probability p_{sub}. A specific substitution error occurs with probability $(p_{\mathrm{sub}}/3)$. The function $\phi(x, y)$ mathematically represents the probability for substitutions of different strand^{C} nucleotides.

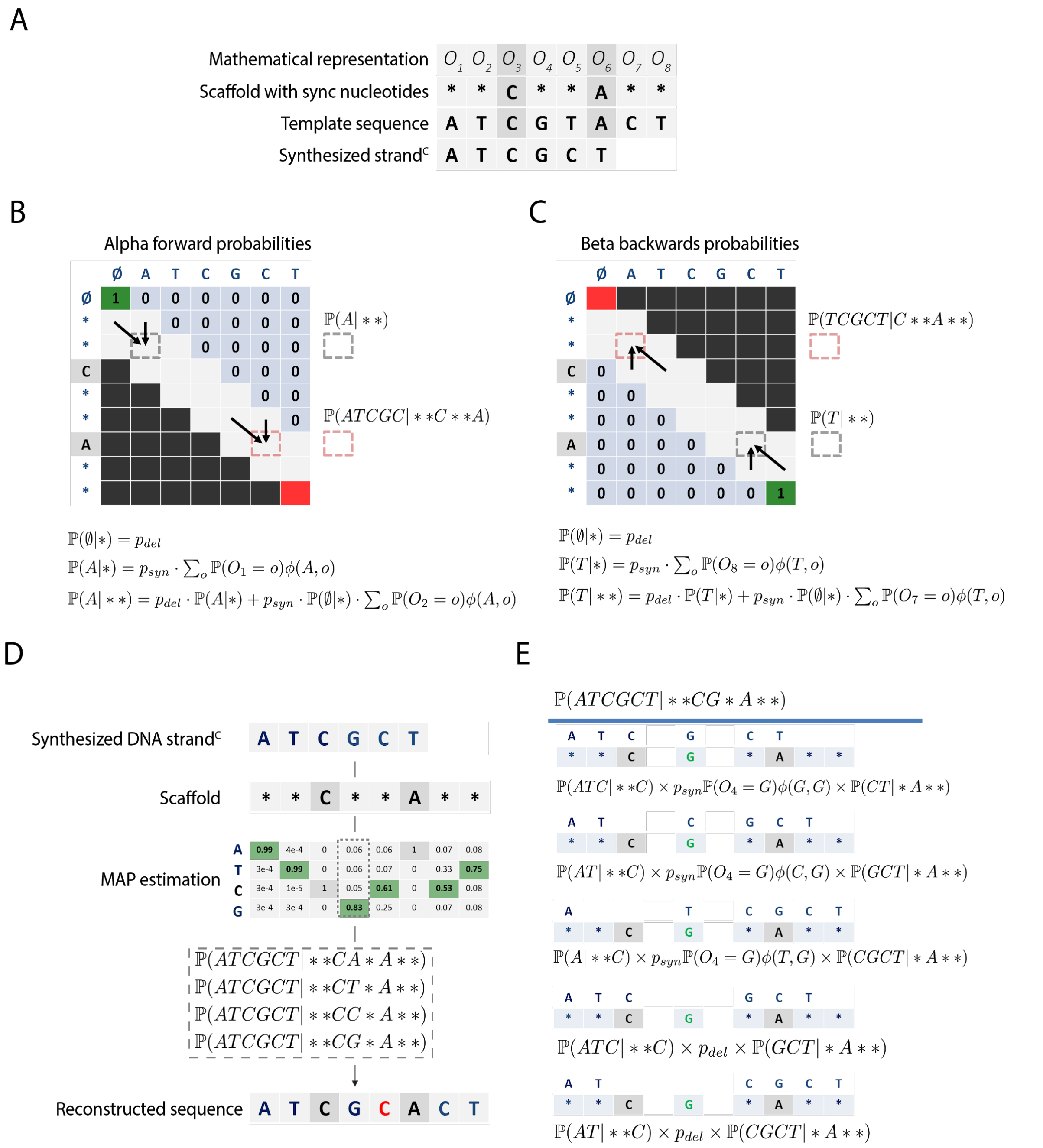

Supplementary Figure 17. Reconstruction of a template sequence by MAP estimation.

A template sequence may be successfully reconstructed from multiple DNA strands^{C}. Even based on a single received strand^{C}, MAP estimation achieves the correct localization of errors given a scaffold sequence containing synchronization nucleotides. An example of a template DNA sequence, associated scaffold sequence, and mathematical representation is given in (A).

The sequence is synthesized, resulting in one DNA strand^{C} containing two missing nucleotides. The goal of MAP estimation is to reconstruct the template DNA sequence given only the received strand^{C} and scaffold. An alpha table and beta table are computed in (B) and (C) respectively, given only strand^{C} and scaffold. The entries of the alpha and beta tables represent alpha forward probabilities and beta backward probabilities, and are computed incrementally and efficiently based on dynamic programming recursions (**Supplementary Note 2**). These alpha and beta probabilities are necessary for the MAP estimation of each nucleotide in the template sequence as illustrated in (D) and (E). Specifically, an example of decoding the fourth nucleotide O_4 of the template sequence is provided in (D). This decoding involves determining the following probabilities: $\mathbb{P}(ATCGCT \mid ** CA * A **)$, $\mathbb{P}(ATCGCT \mid ** CT * A **)$, $\mathbb{P}(ATCGCT \mid ** CC * A **)$, and $\mathbb{P}(ATCGCT \mid ** CG * A **)$ each representing the fact that either an A, T, C, or G is possible for the fourth nucleotide respectively. The decomposition of the probability $\mathbb{P}(ATCGCT \mid ** CG * A **)$ into different cases is given in (E). The result of MAP estimation applied for all nucleotides reveals that a nearly correct reconstruction of the template sequence is possible even with one received DNA strand^{C}, and that errors may be localized to their proper positions within the sequence.

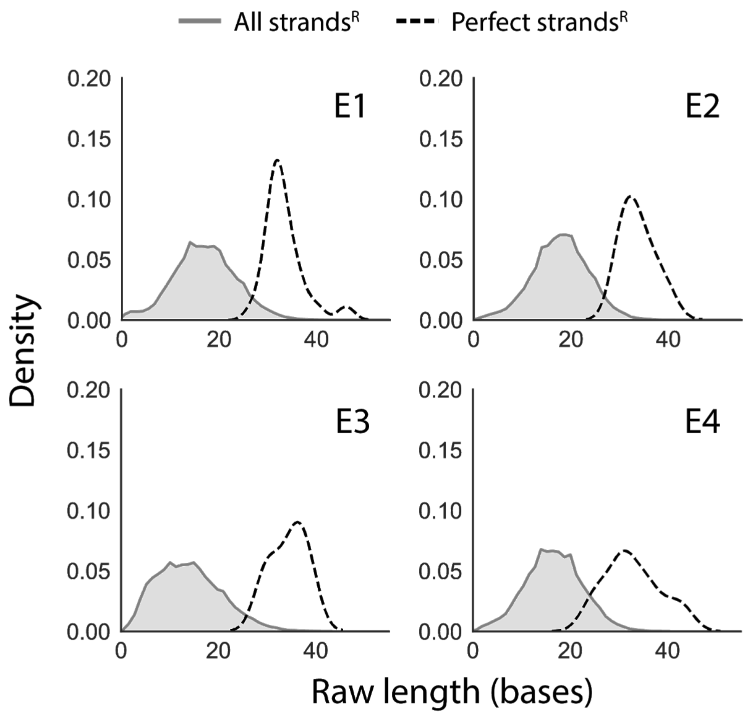

Supplementary Figure 18. Raw lengths for all and perfect strands for E1-E4.

All synthesized strands of E1-E4 were sequenced with Illumina. Length distribution for the set of all (gray with shading) and perfectly (dashed line) synthesized and sequenced raw strands are shown. Distributions are derived via kernel density estimation. The number of all strands to perfect strands for each template sequence are as follows: E1 {all: 113322, perfect: 21}; E2 {all: 104517, perfect: 9}; E3 {all: 102545, perfect: 3}; E4 {all: 141072, perfect: 19}.

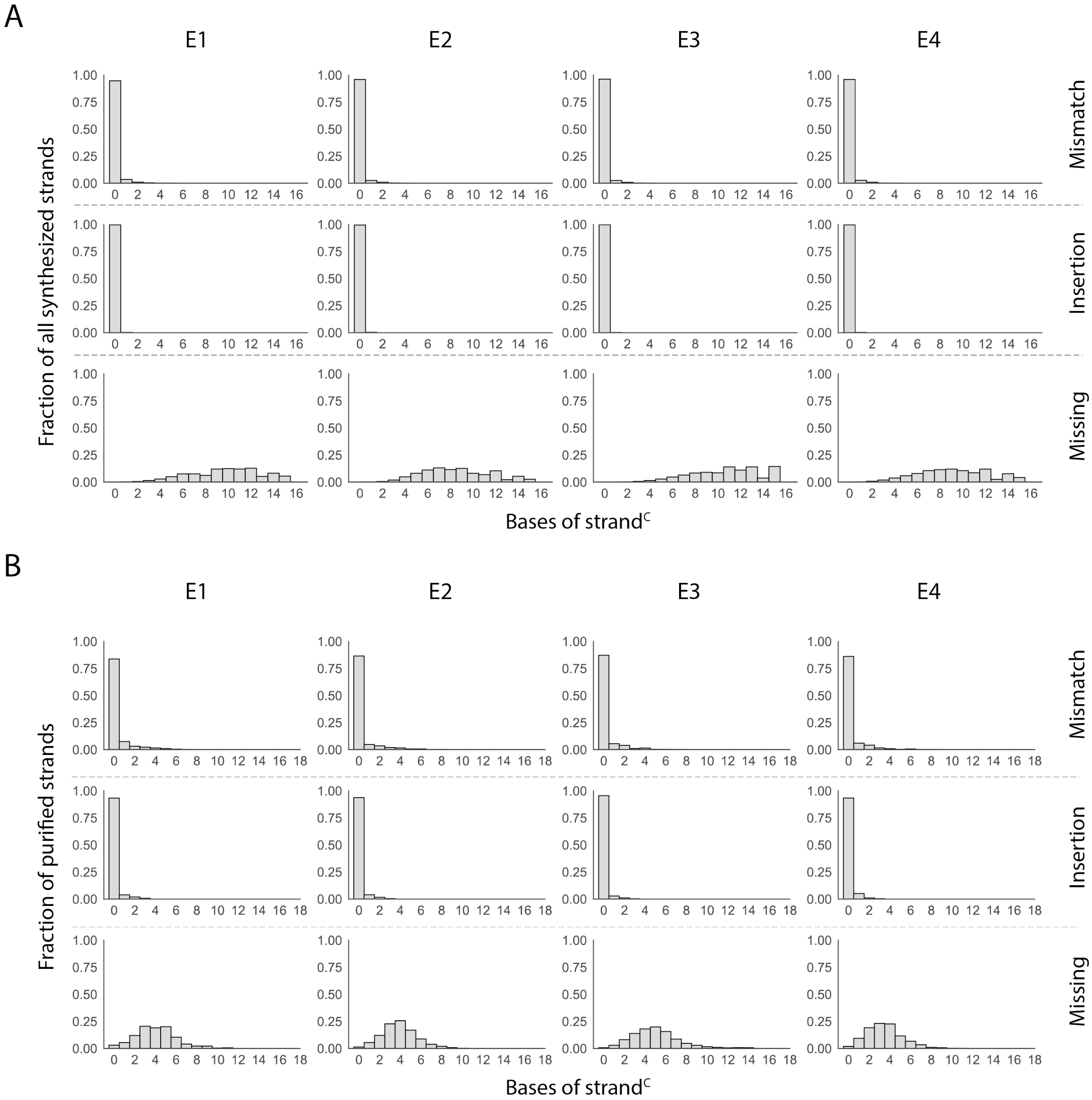

Supplementary Figure 19. Synthesis error analysis for all strands and purified strands of E1-E4.

All synthesized strands^R were sequenced with Illumina and transitions extracted to form strands^C. Each of these strands^C is aligned, by Needleman-Wunsch, to its respective template sequence. For each alignment, the fraction of strands with the indicated number of mismatches, insertions, and missing nucleotides are tabulated. The set of all strands are evaluated in (A) and the set of purified strands obtained by filtering the length of the corresponding strands^R between

32-48 bases, assuming an extension length of 3 to 4 bases per template nucleotide are evaluated in (B).

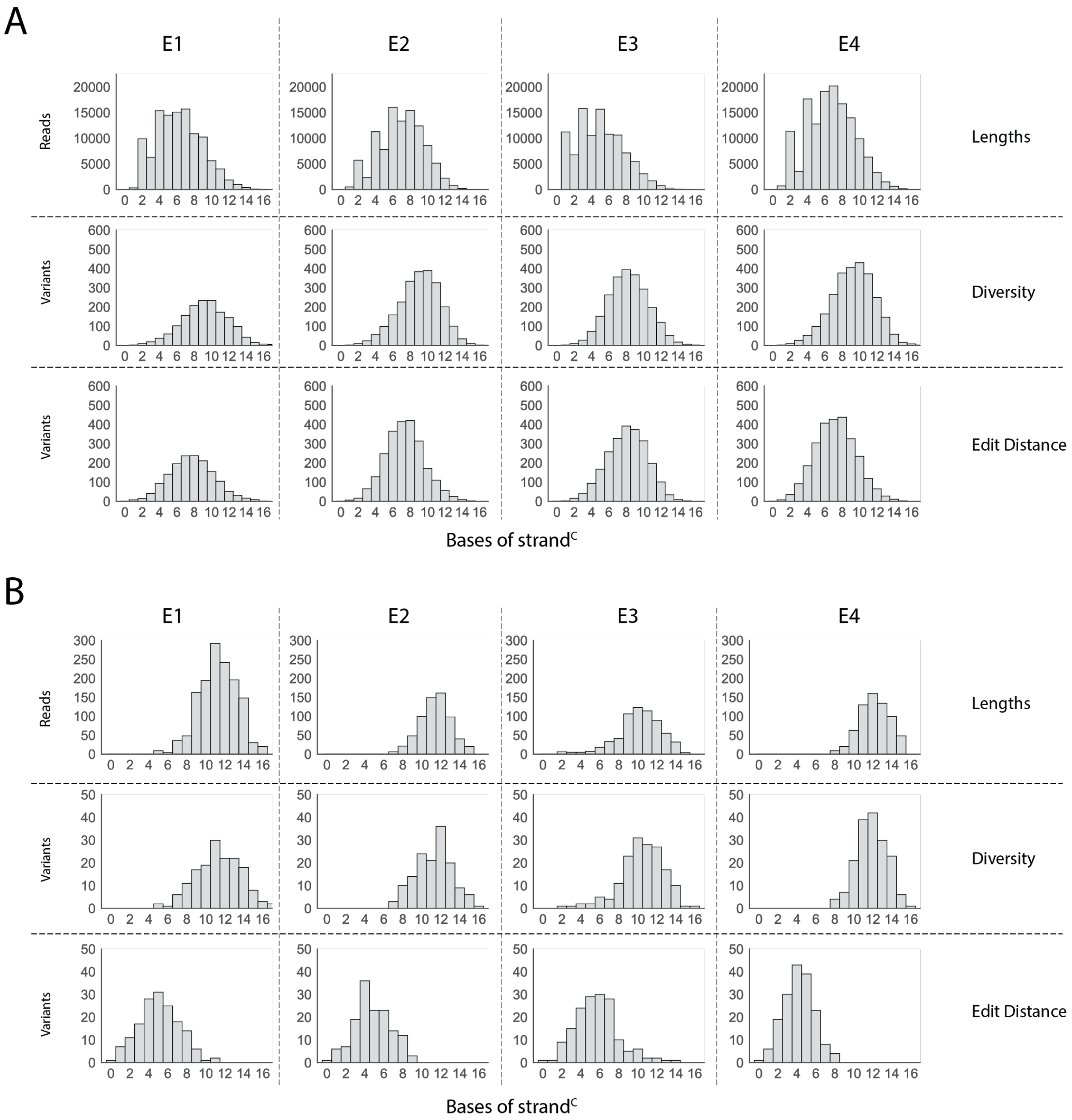

Supplementary Figure 20. Lengths, diversity, and edit distance for all and purified strands for E1-E4.

All synthesized strands^{R} of E1-E4 were sequenced with Illumina and transitions extracted to form strands^{C}. Strands^{C} were filtered for read counts of at least 2 to remove aberrantly synthesized or sequenced variants. The number of sequencing reads at each length (number of strand^{C} nucleotides) is tabulated. Diversity is evaluated as the number of unique variants at each

length and the Levenshtein edit distance is computed according to its respective template sequence. These measurements are presented for all synthesized strands^{C} (A) or a set of purified strands^{C} obtained by filtering the length of the corresponding strands^{R} between 32-48 bases, assuming an extension length of 3 to 4 bases per template nucleotide are evaluated in (B).

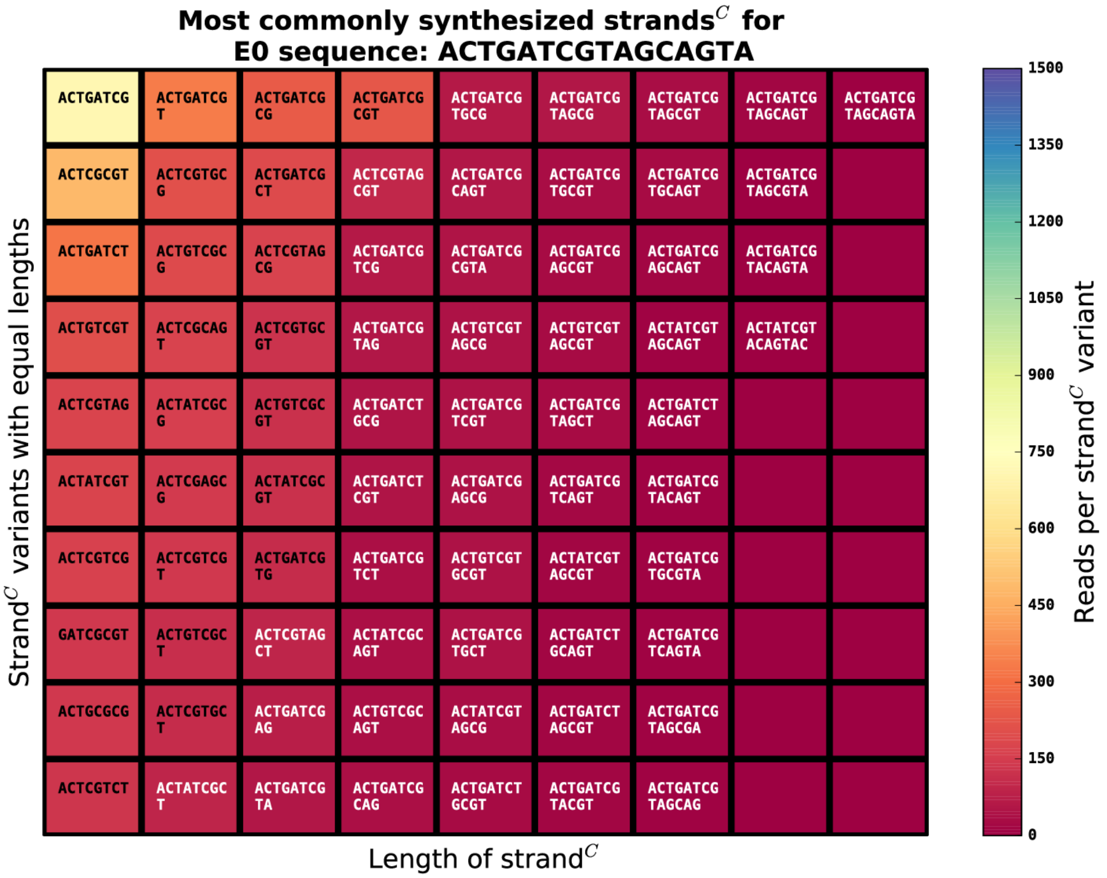

Supplementary Figure 21. Diversity of compressed synthesized strands for E0.

Strands^{C} obtained for template sequence E0. Different strand variants are ranked in the vertical axis in order of the number of reads per variant. The strands are arranged on the horizontal axis in order of increasing length. In comparison to the E0 template sequence, most diverse compressed strands are missing nucleotides, although some strands may have insertions or mismatches (substitutions).

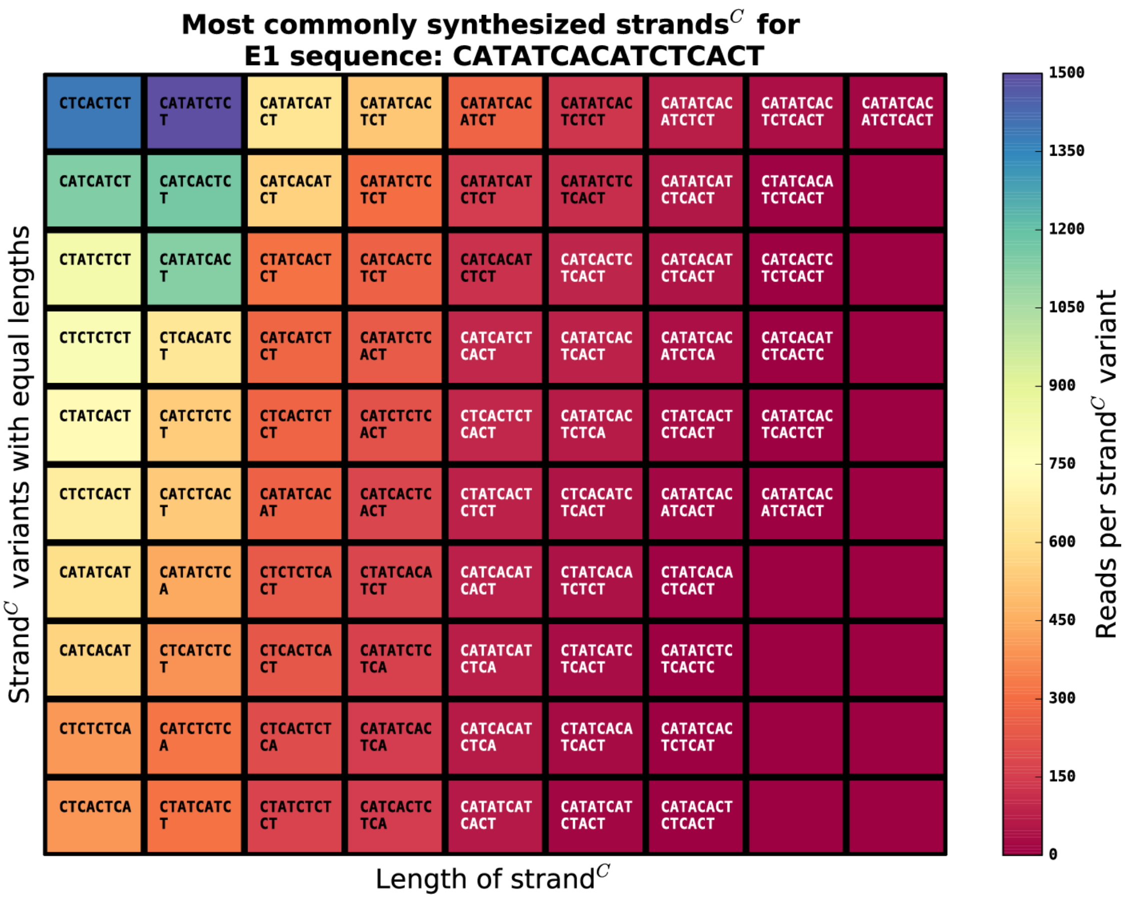

Supplementary Figure 22. Diversity of compressed synthesized strands for E1.

Strands^C obtained for sequence E1. Different strand variants are ranked in the vertical axis in order of the number of reads per variant. The strands are arranged on the horizontal axis in order of increasing length. In comparison to the E1 template sequence, most diverse compressed strands are missing nucleotides, although some strands may have insertions or mismatches (substitutions).

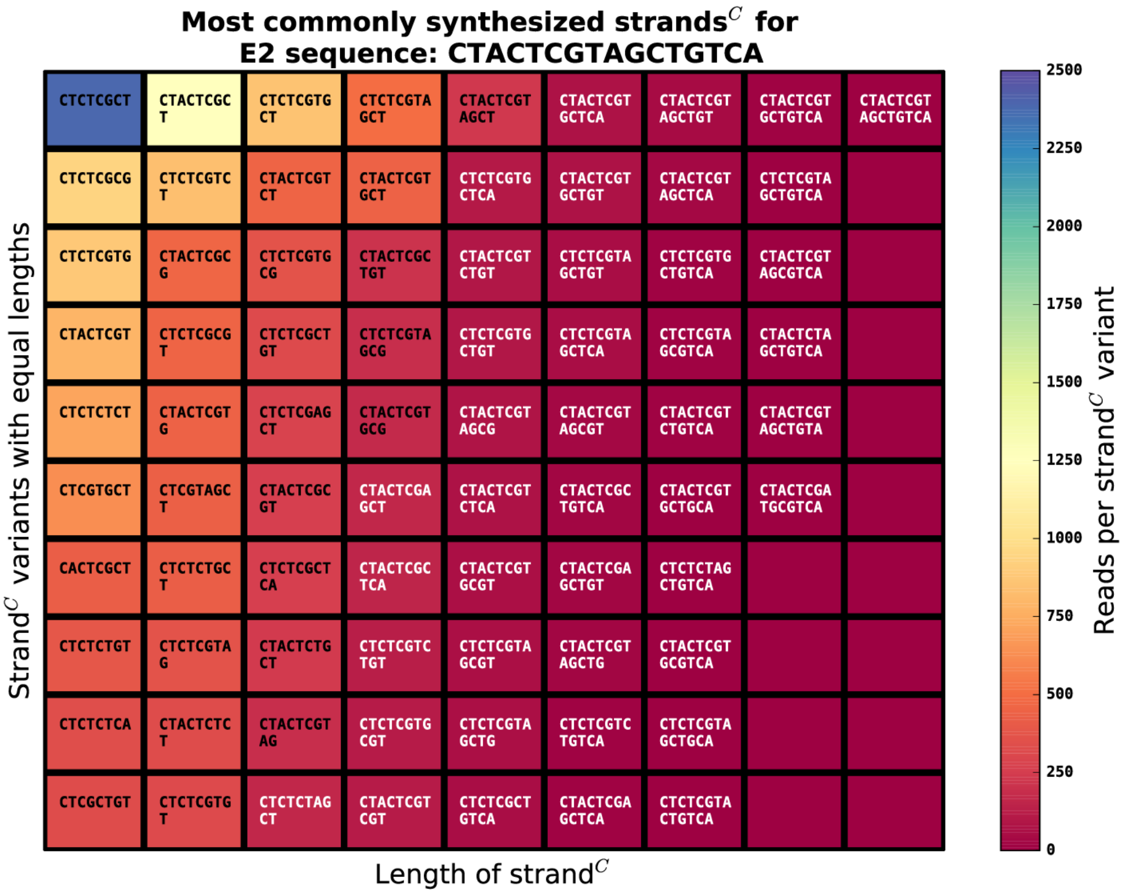

Supplementary Figure 23. Diversity of compressed synthesized strands for E2.

Strands^C obtained for sequence E2. Different strand variants are ranked in the vertical axis in order of the number of reads per variant. The strands are arranged on the horizontal axis in order of increasing length. In comparison to the E2 template sequence, most diverse compressed strands are missing nucleotides, although some strands may have insertions or mismatches (substitutions).

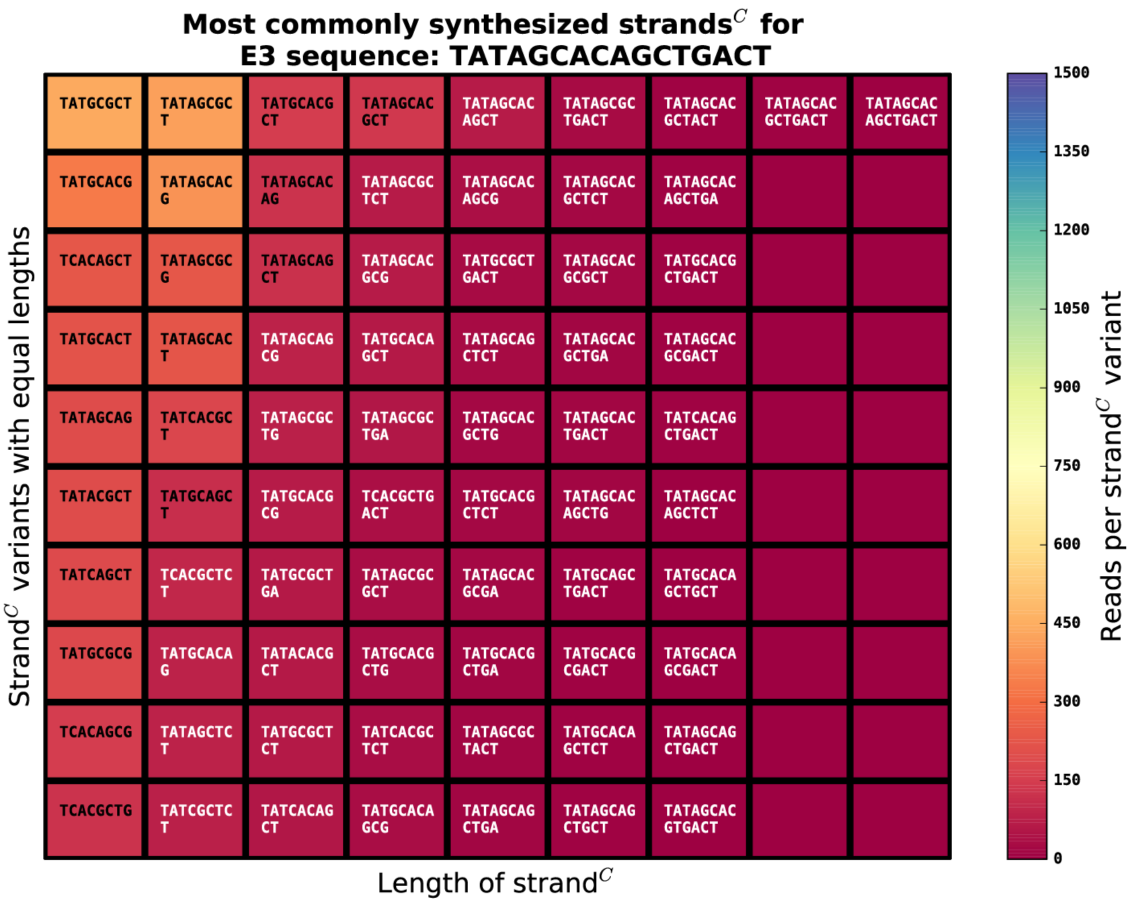

Supplementary Figure 24. Diversity of compressed synthesized strands for E3. Strands^C obtained for sequence E3. Different strand variants are ranked in the vertical axis in order of the number of reads per variant. The strands are arranged on the horizontal axis in order of increasing length. In comparison to the E3 template sequence, most diverse compressed strands are missing nucleotides, although some strands may have insertions or mismatches (substitutions).

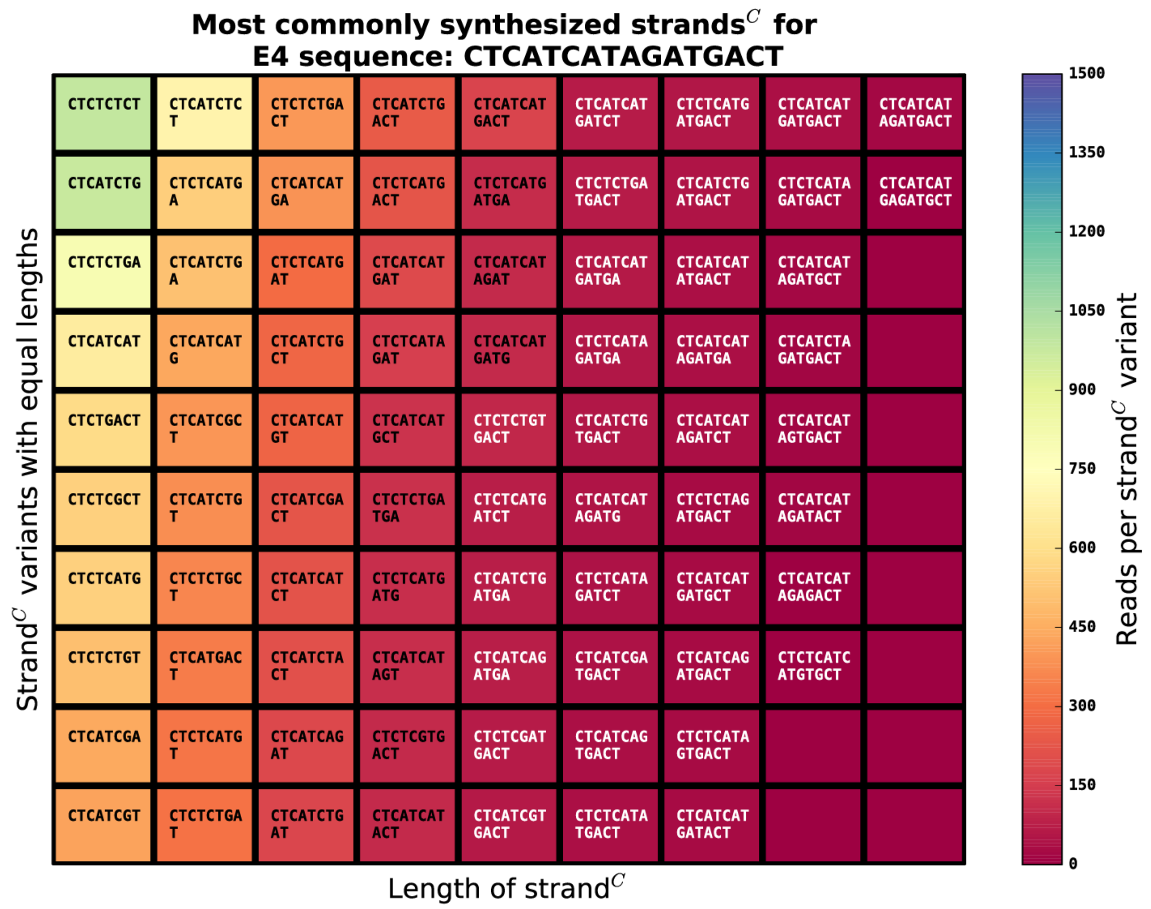

Supplementary Figure 25. Diversity of compressed synthesized strands for E4.

Strands^C obtained for sequence E4. Different strand variants are ranked in the vertical axis in order of the number of reads per variant. The strands are arranged on the horizontal axis in order of increasing length. In comparison to the E4 template sequence, most diverse compressed strands are missing nucleotides, although some strands may have insertions or mismatches (substitutions).

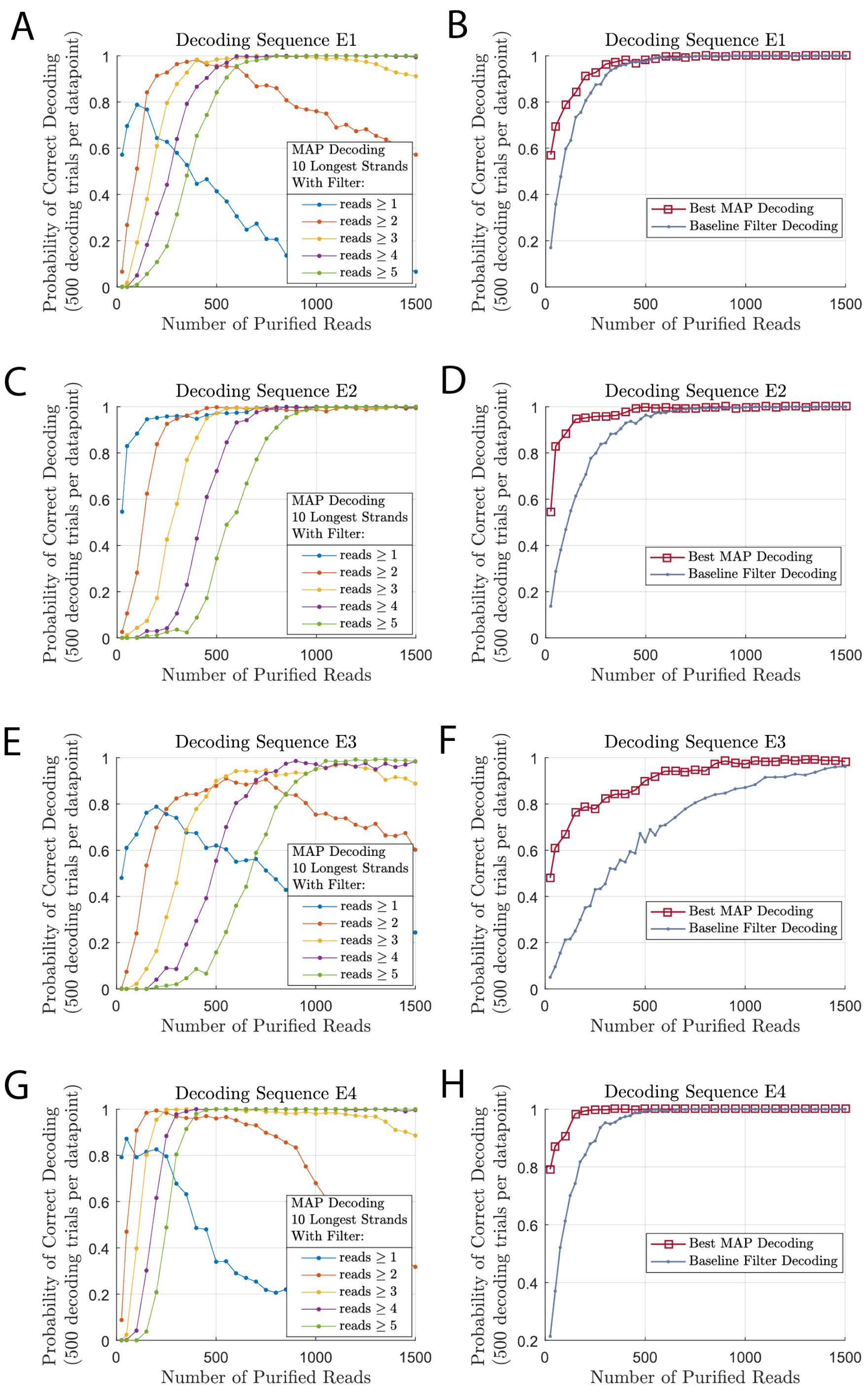

Supplementary Figure 26. Decoding curves for E1-E4 template sequences for “Eureka!”.

Results for the successful reconstruction of sequences E1-E4 from the *in silico* size-selected set of DNA strands[C]. All decoding curves illustrate the probability of correct decoding of a sequence vs. the number of purified reads of synthesized DNA strands[C]. For each datapoint on a curve, the

probability of correct decoding is based on 500 decoding trials, each of which involves sampling a set of purified DNA strands according to the target number of total reads. In each decoding trial, the sampled set of DNA strands is filtered further based on the number of reads per strand (between 1 and 5 reads per strand). The 10 strands with the longest length are selected for reconstruction via MAP decoding and consensus. Decoding curves are presented for sequences E1-E4 in (A), (C), (E), and (G) respectively when applying the different filters based on reads per strand. The best decoding results from the filters are compiled for each datapoint to produce the “Best MAP Decoding” curve in (B), (D), (F), and (H). This curve is compared to the two-step baseline filter, used for H01-H12, decoding which outputs the longest DNA strand which also has the highest number of reads amongst other strands of equal length. Taken together, these results show that decoding accuracy improves substantially when applying MAP decoding and consensus with 10 filtered strands compared to baseline decoding with one filtered strand.

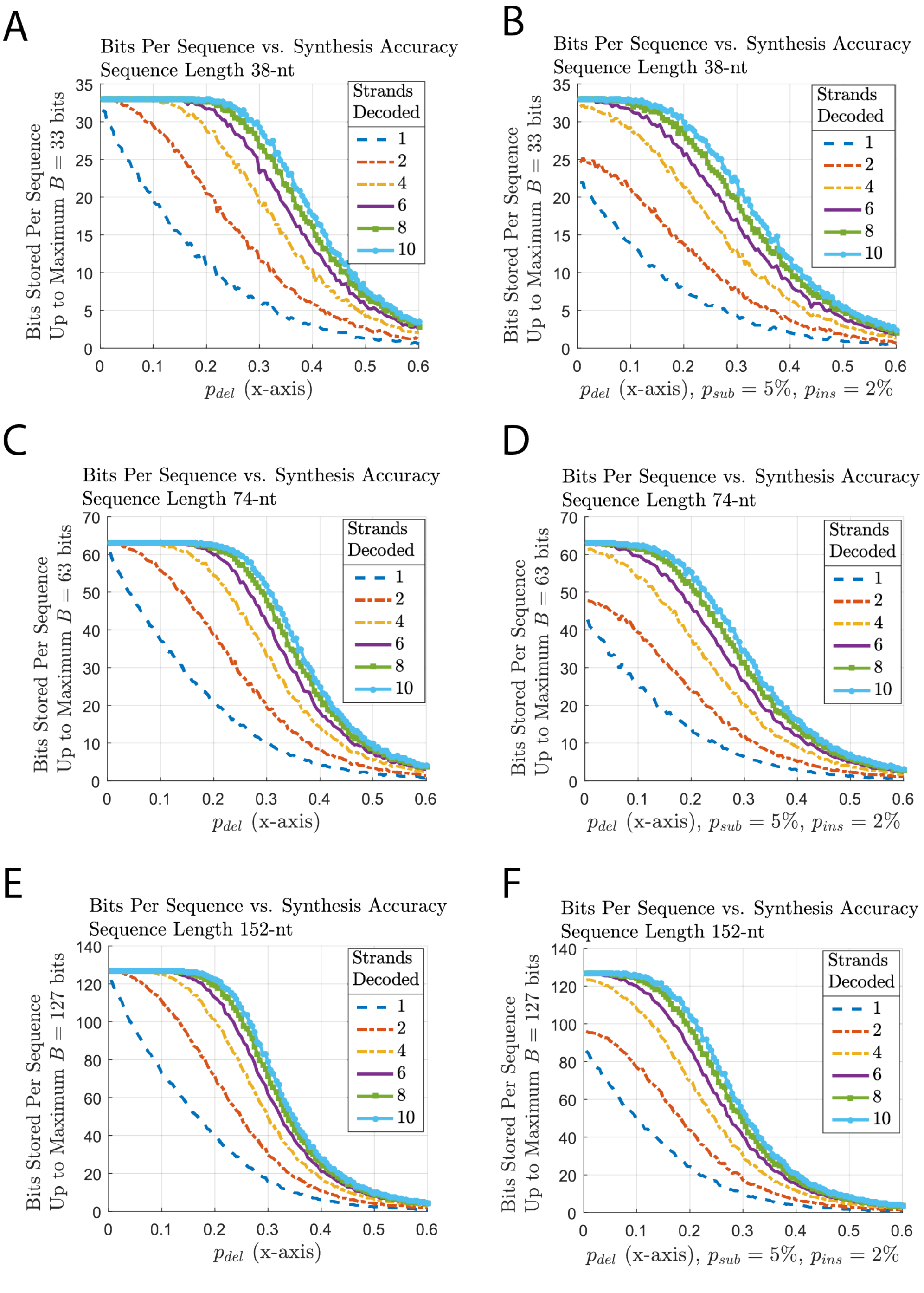

Supplementary Figure 27. Estimated capacity in bits per template sequence with increased synthesis accuracy for simulated DNA storage systems.

Tradeoffs between estimated capacity (bits stored per sequence) vs. synthesis accuracy. For template sequences with 38 nucleotides, (A) estimated capacity vs. synthesis accuracy measured in terms of the probability of deletions only (missing nucleotides) or (B) including additional 5%

substitution and 2% insertion errors. For template sequences with 74 nucleotides, (C) estimated capacity vs. synthesis accuracy measured in terms of the probability of deletions only (missing nucleotides) or (D) including additional 5% substitution and 2% insertion errors. For sequences with 152 nucleotides, (E) estimated capacity vs. synthesis accuracy measured in terms of the probability of deletions only (missing nucleotides) or (F) including additional 5% substitution and 2% insertion errors. The estimated capacity decreases smoothly as synthesis accuracy decreases. The tradeoffs are non-linear. If more compressed strand variants are utilized for decoding, the estimated capacity increases.

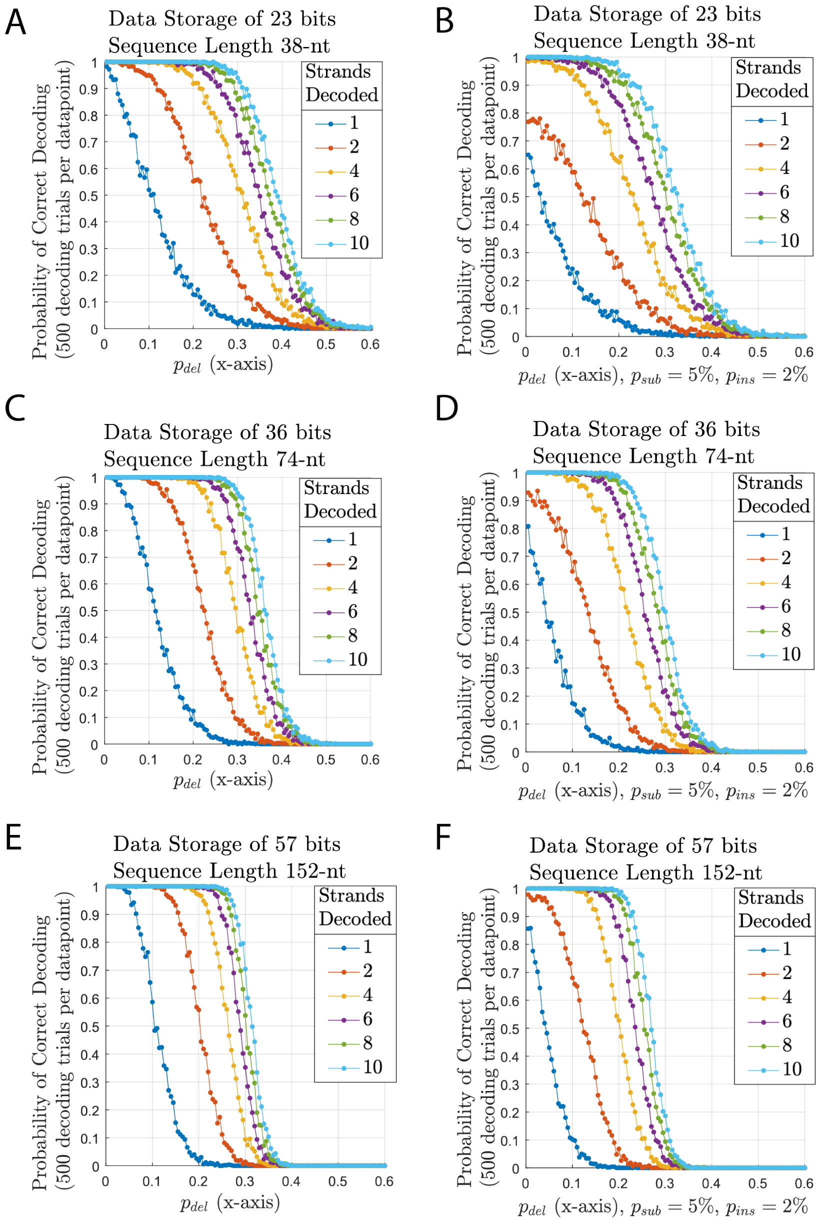

Supplementary Figure 28. Waterfall decoding curves for simulated DNA storage systems.

Simulation results for successfully decoding and retrieving information from multiple DNA strands synthesized per sequence. Decoding results are visualized as “waterfall curves”, representing the probability of correct retrieval for varying levels of errors tolerated per strand. The boundary of error-tolerance for all displayed systems is between 25-30% per strand[C], including missing nucleotides (deletions), mismatches (substitutions), and insertion errors. This

error tolerance is obtained for decoding with up to 10 diverse strands^{C} per sequence. (A) Decoding 23 bits of information stored in template sequences of 38 nucleotides, based on multiple strands^{C} containing only missing nucleotides and (B) with the inclusion of mismatches (substitutions) and insertion errors. (C) Decoding 36 bits of information stored in template sequences of 74 nucleotides, based on multiple strands^{C} containing only missing nucleotides and (D) with the inclusion of mismatches (substitutions) and insertion errors. (E) Decoding 57 bits of information stored in template sequences of 152 nucleotides, based on multiple strands^{C} containing only missing nucleotides and (F) with the inclusion of mismatches (substitutions) and insertion errors.

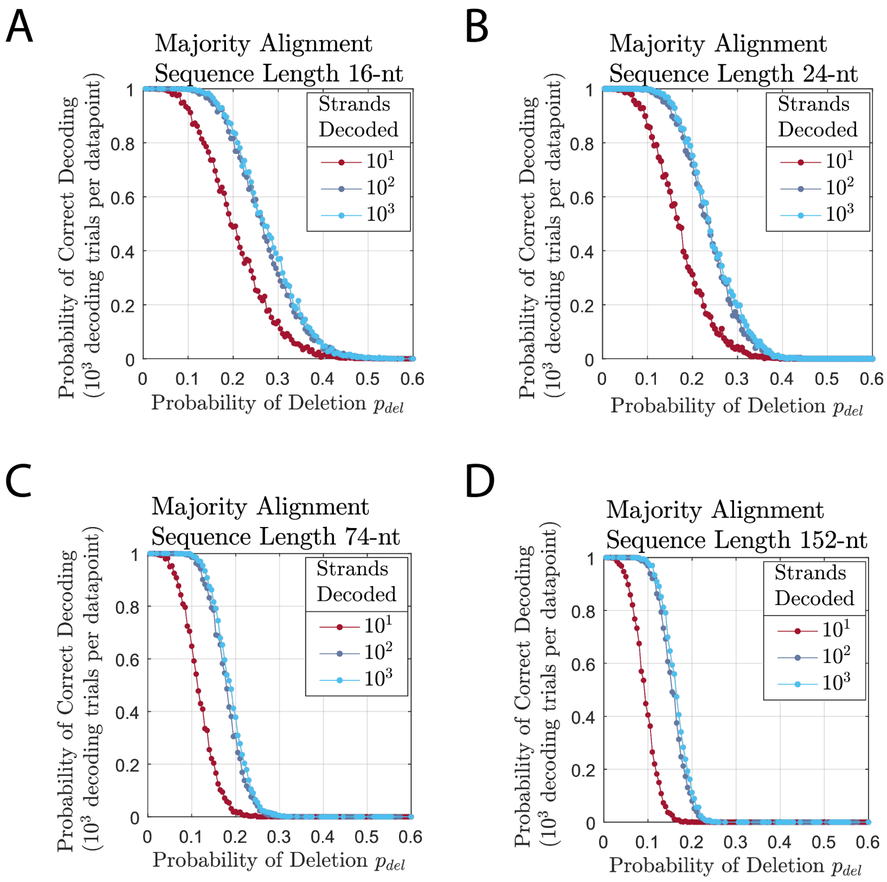

Supplementary Figure 29. Majority alignment of DNA strands per sequence.

Simulation results for decoding sequences using the majority alignment algorithm. Template sequences have (A) 16, (B) 24, (C) 74, and (D) 152 nucleotides respectively. Each template sequence is randomly created per decoding trial. A total of 1000 decoding trials were simulated per datapoint. The production of DNA strands from a template sequence is simulated according to a Markov model with probability of deletion p_{del} per nucleotide. Sequences are decoded from either 10, 100, or 1000 diverse strands[C]. Majority alignment achieves an increase in decoding accuracy given more strands[C]. However, the decoding accuracy reaches a theoretical limit. The error-tolerance saturates at approximately $p_{\text{del}} = 12\%$.

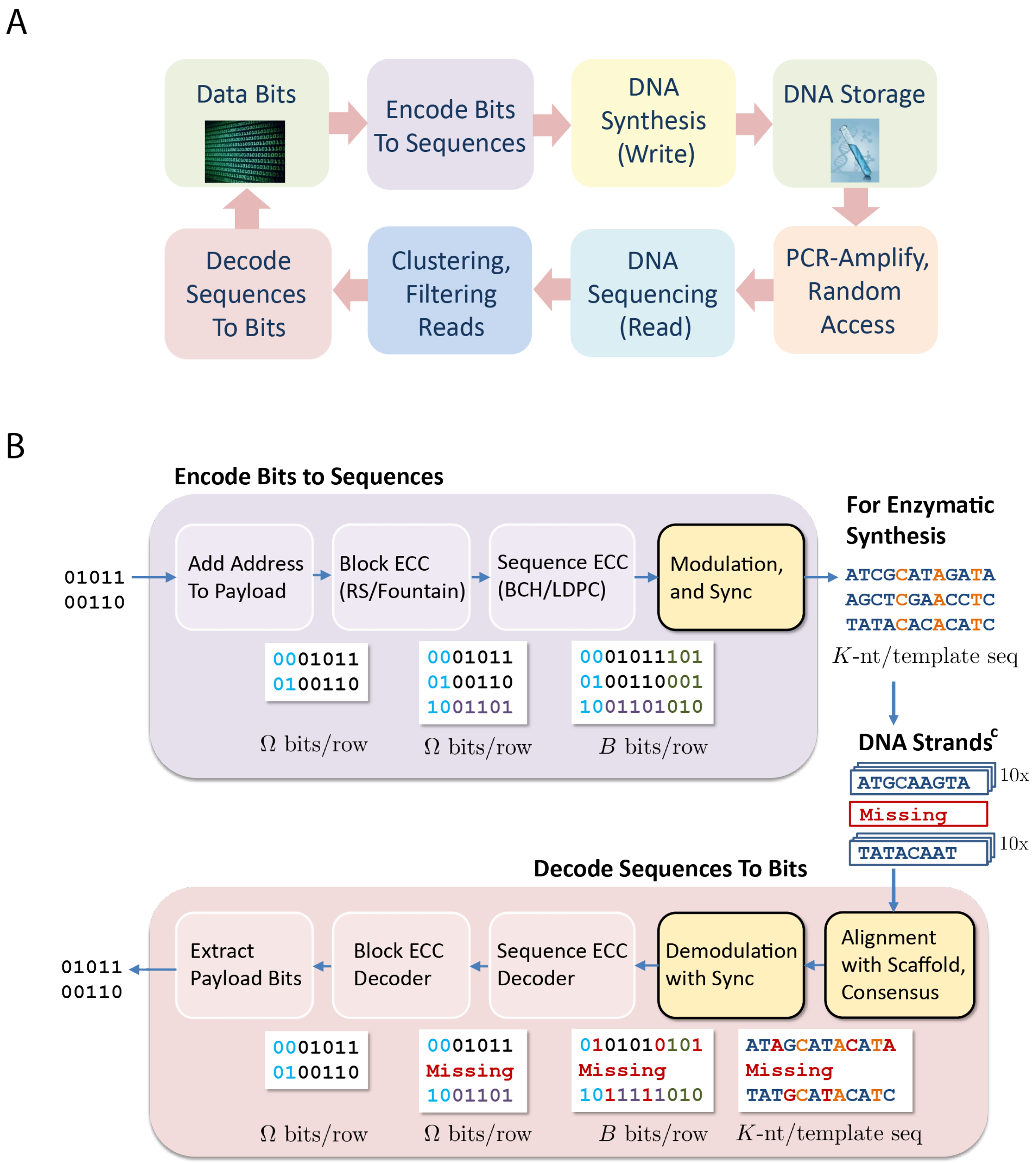

Supplementary Figure 30. System architecture of codec for storing information in DNA.

(A) A high-level block diagram of a DNA storage system. Data is represented as bits of information which are encoded into a set of DNA sequences. *De novo* synthesis (e.g., enzymatic synthesis) of each sequence results in the creation of diverse DNA strands which can be stored at high volumetric density. For random-access retrieval of data, a subset of the DNA strands may be PCR-amplified and then sequenced (e.g., using Illumina or nanopore sequencing technologies). DNA sequencing results in several reads. All reads are clustered, filtered,

processed *in-silico*, and provided to a decoder for reconstruction. The decoder applies several steps to reconstruct the original DNA sequences, and to decode the original bits of information. (B) A detailed block diagram of a codec for robust storage of digital information in DNA (**Supplementary Note 2**). The encoder first partitions payload data into rows of bits. Each row is prefixed with an address (turquoise) to delineate its order. To recover missing rows of data, an error-correction code (ECC) may be applied per block of rows, resulting in redundant rows of information (purple). Additionally, an ECC may be applied per row/sequence of data, resulting in redundant bits per row (light green). Each row of bits is modulated into a DNA sequence of nucleotides (blue) containing interspersed synchronization nucleotides (orange). Synthesis of each sequence results in diverse compressed strands which may contain nucleotide errors (red). We assume that compressed strands can be sufficiently clustered (**Supplementary Note 4**). The decoder fully or partially reconstructs DNA sequences from compressed strands using MAP estimation and consensus algorithms. After demodulation of DNA sequences to rows/sequences of bits, the decoder may apply ECC decoding per row/sequence to correct remaining bit errors (red). The decoder then orders all rows according to their addresses. If any rows are missing, additional error-correction may be applied to recover rows using a block ECC. The final step of the decoder is to extract the original payload data from the ordered rows of bits. Overall, the encoding and decoding pipelines ensure the robust storage of data in DNA.

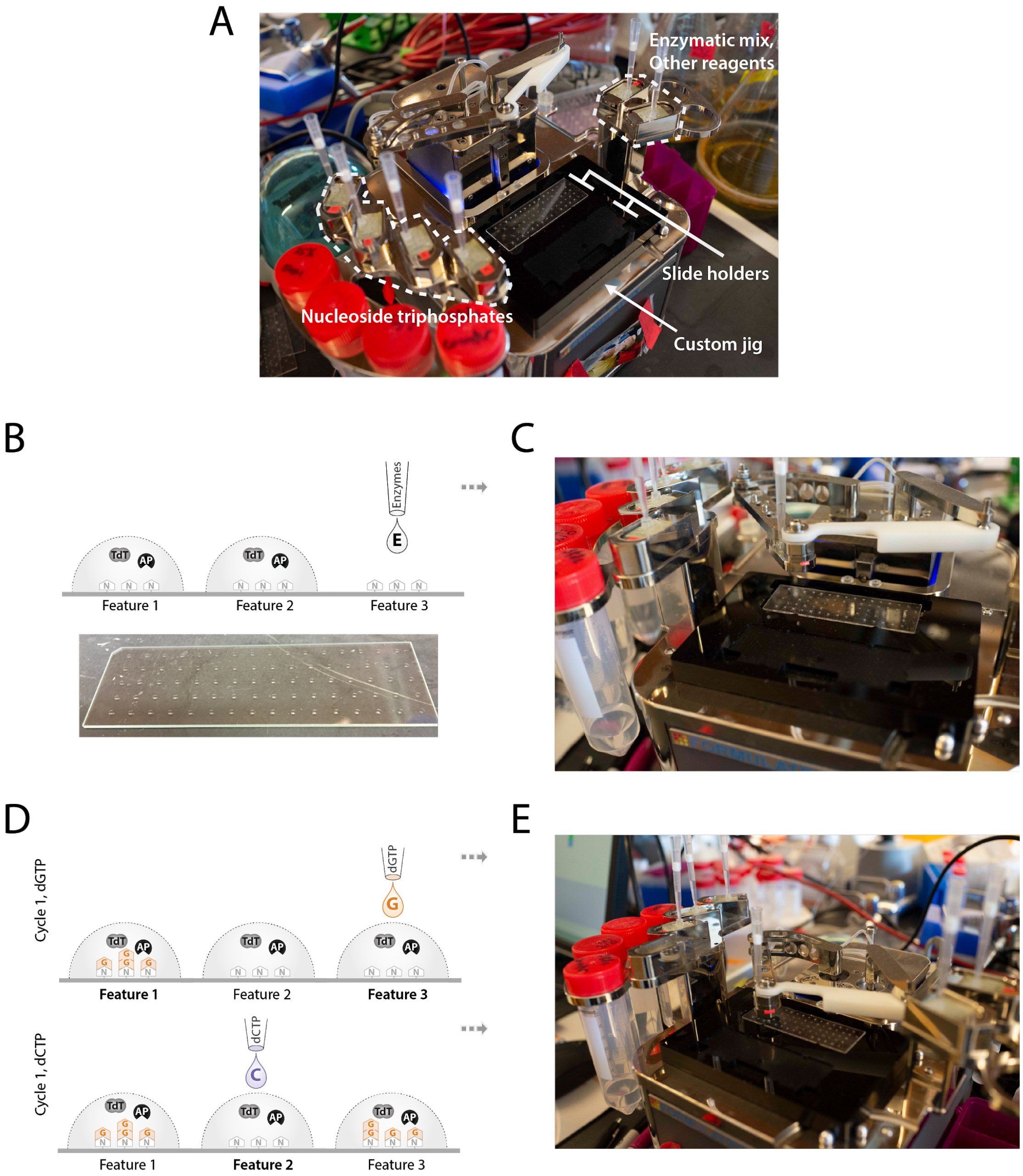

Supplementary Figure 31. Array-format enzymatic synthesis platform.

A prototype for enzymatic synthesis of DNA strands in a 2D array format. (A) The prototype is comprised of two main parts: a Mantis liquid handler, which has a single robotic arm that can be programmed to dispense one of six reagents at a time, and custom jigs, which were either laser cut (Epilog Legend 36EXT) or machined (gift from Formulatrix) to hold the glass slide acting as a solid support substrate for the DNA. In our reagent banks, we have four nucleoside triphosphates and one enzymatic mix. For attaching initiators and detaching synthesized strands

from the slide, other reagents can be dispensed by changing the dispense head. (B) Enzymatic mix is dispensed according to programmed coordinates on the treated slide, resulting in a 2D grid of features. (C) The Mantis places the enzymatic mix, according to programmed coordinates, in serial to all features on the slide. (D) For each synthesis cycle, there are four dispense cycles, one for each of the four nucleoside triphosphates used. The specific nucleoside triphosphate is dispensed only to the desired features (bold). (E) The Mantis has a single dispenser and places the nucleoside triphosphate, according to programmed coordinates, in serial to the desired features on the slide.

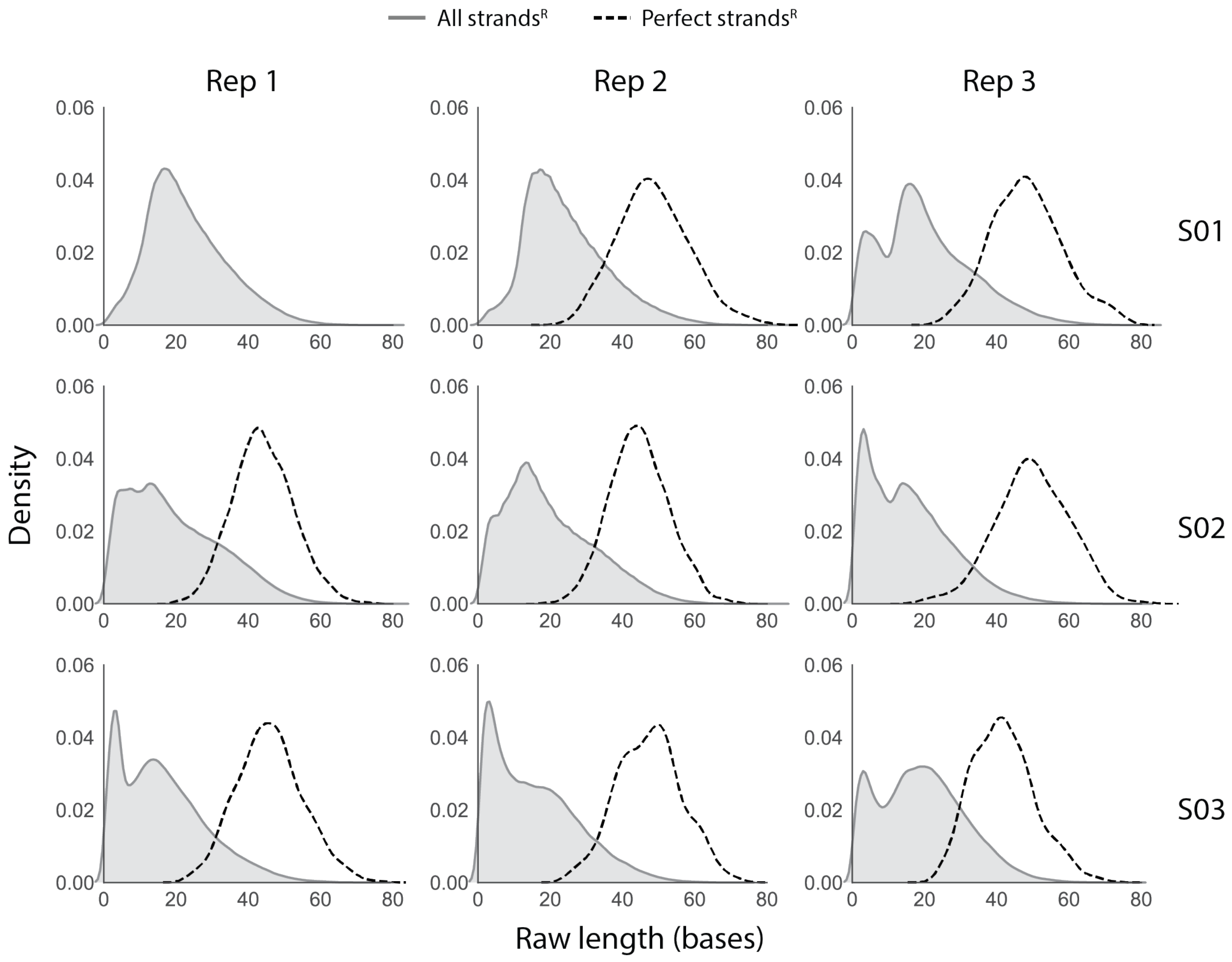

Supplementary Figure 32. Raw lengths for all and perfect raw strands for S01-S03.

Length distribution for the set of all (gray shading) and perfectly (dashed line) synthesized and sequenced raw strands. Distributions are derived via kernel density estimation. As perfect reads are longer, on average, size selection will increase the yield of perfectly synthesized strands. The number of all strands and perfect strands for each template sequence are as follows: S01 rep1 {all: 192989, perfect: 1}, S01 rep 2 {all: 220921, perfect: 684}, S01 rep 3 {all: 153002, perfect: 286}, S02 rep 1 {all: 277897, perfect: 3545}, S02 rep 2 {all: 385615, perfect: 4889}, S02 rep 3 {all: 176680, perfect: 248}, S03 rep 1 {all: 185327, perfect: 464}, S03 rep 2 {all: 169000, perfect: 273}, S03 rep 3 {all: 209018, perfect 898}. The S01 rep 1 distribution for perfect strands is not visible due to the low number of perfect strands.

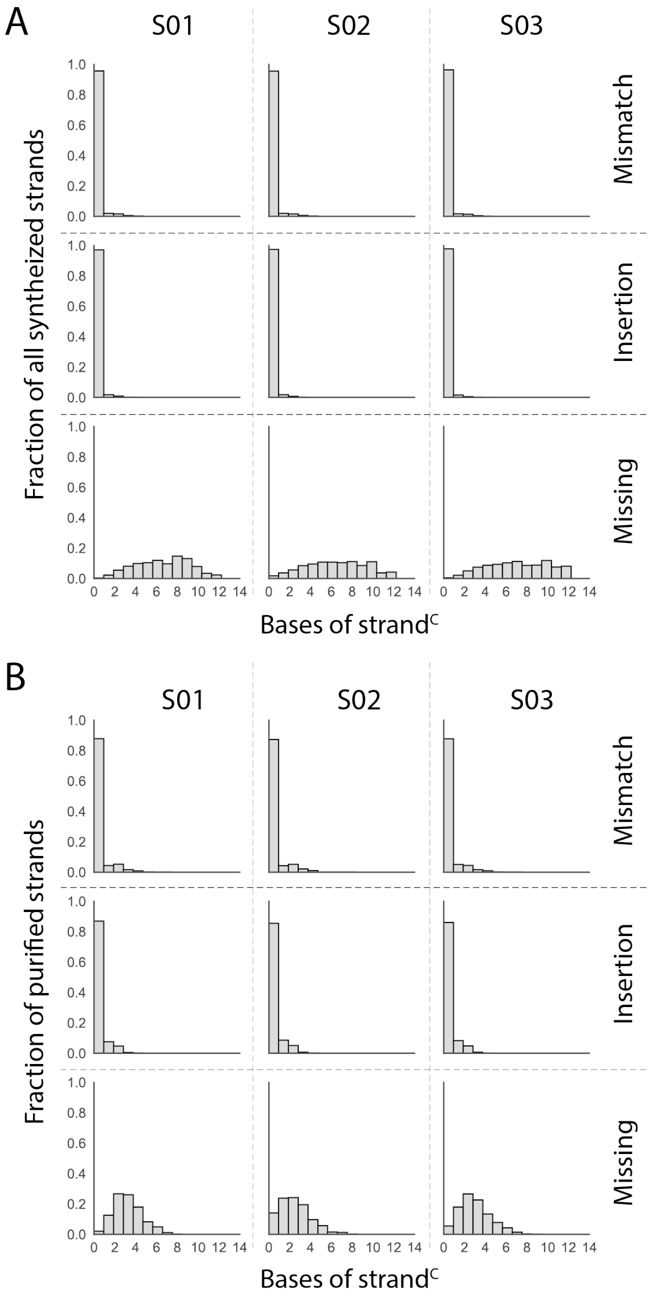

Supplementary Figure 33. Synthesis error analysis for all and purified strands for S01-S03.

All synthesized strands^R were sequenced with Illumina and transitions extracted to form strands^C. Each of these strands^C is aligned, by Needleman-Wunsch, to its respective template sequence. For each alignment, the fraction of strands^C with the indicated number of mismatches,

insertions, and missing nucleotides are tabulated. The set of all strands are evaluated in (A) and the set of purified strands obtained by filtering the length of the corresponding strands^R between 39-52 bases, assuming an extension length of 3 to 4 bases per template nucleotide are evaluated in (B).

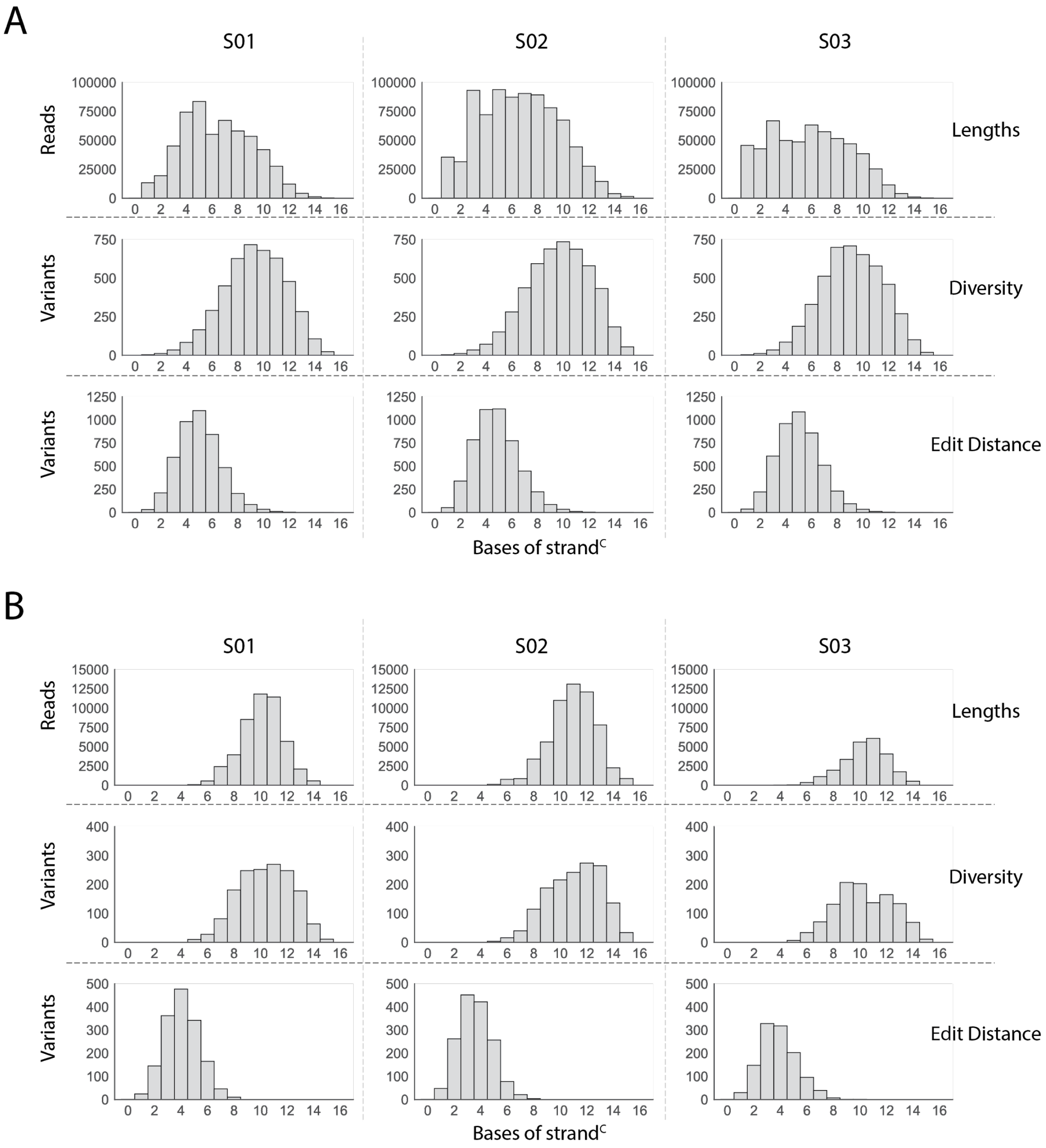

Supplementary Figure 34. Lengths, diversity, and edit distance for all and purified strands for S01-S03.

All synthesized strands^R of S01-S03 were sequenced with Illumina and transitions extracted. Run-length compressed strands (strands^C) were filtered for read counts of at least 3 to remove aberrantly synthesized or sequenced variants. The number of sequencing reads at each length

(number of strand^C nucleotides) is tabulated. Diversity is evaluated as the number of unique variants at each length and the Levenshtein edit distance is computed according to its respective template sequence. These measurements are presented for all synthesized strands^C (A) or a set of purified strands^C obtained by filtering the length of the corresponding strands^R between 39-52 bases, assuming an extension length of 3 to 4 bases per template nucleotide are evaluated in (B).

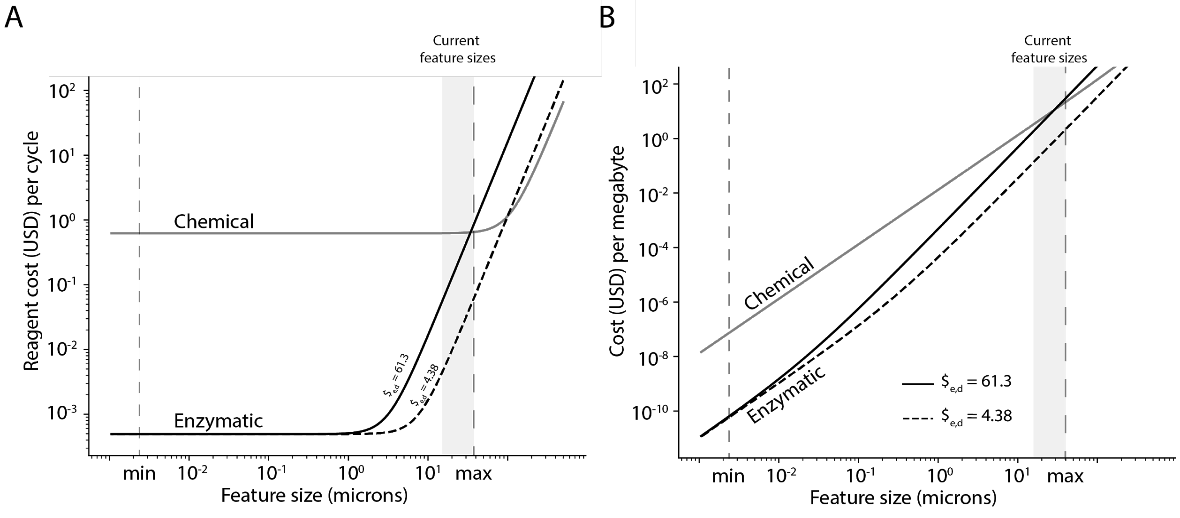

Supplementary Figure 35. Reagent cost projections for phosphoramidite chemistry and enzymatic synthesis.

(A) Reagent cost per cycle projections for phosphoramidite chemistry (gray line) and enzymatic synthesis (black lines) based on estimated number of features ($n = 1{,}000{,}000$) and density of 71,000 features per square cm ($D = 71{,}000$) of the Agilent SurePrint G3 system. Commercial pricing of enzymes $\$_{d,e}$= 61.3 (solid black line) and bulk pricing with enzyme recycling $\$_{d,e}$= 4.38 (dashed black line) are indicated. The minimum feature size is 2.37 nm, which corresponds to the diameter of double-stranded DNA, and the maximum feature size is 37.5 microns, which corresponds to having no gap between features for the given density. Current feature sizes are estimated to be between 15 (left edge, gray box) to 38 (right edge, gray box) microns based on dispense volumes between 1-10 picoliters. See **Supplementary Note 4** for model details. (B) Reagent cost per megabyte projections for phosphoramidite chemistry (gray line) and enzymatic synthesis (black lines) based on maximally packing features into a given 14cm^2 surface area ($A = 14$) and an efficiency rate of storage of 1 bit per template nucleotide. Commercial pricing of enzymes $\$_{d,e}$= 61.3 (solid black line) and bulk pricing with enzyme recycling $\$_{d,e}$= 4.38 (dashed black line) are indicated. The minimum feature size is 2.37 nm, which corresponds to the diameter of double-stranded DNA. The price per megabyte for 1 million features with current feature sizes of between 15 (left edge, gray box) to 38 (right edge, gray box) microns. See **Supplementary Note 4** for model details.

Supplementary Tables

Supplementary Table 1. Oligonucleotides used in this study.

Name	Sequence
f-P5-SBS3	AATGATACGGCGACCACCGAGAT CTACACTCTTTCCCTACACGACGCTCTTCCGATCT
LT2	AGATCAATTAATACGATACCTGCG
LT2+3A	AGATCAATTAATACGATACCTGCG AAA
LT2+3C	AGATCAATTAATACGATACCTGCG CCC
LT2+3G	AGATCAATTAATACGATACCTGCG GGG
LT2+3T	AGATCAATTAATACGATACCTGCG TTT
5Am12-fSBS3-acgtactgag	/5AmMC12/TTTTTTTTTT UCTACACTCTTTCCCTACACGACGCTCTTCCGATCTACGTACTGAG
5P-rSBS9-GGG	/5Phos/AGATCGGAAGAGCACACGTCTGAACTCCAGTCAC T/ideoxyU/CCGATCT GGG/3SpC3/
tSBS3	CTACACTCTTTCCCTACACGAC
ttSBS9	GTGACTGGAGTTCAGACGTG
PR2-P5	TTTCTGTTGGTGCTGATATTGCAATGATACGGCGACCACCGA
3580F-P7	ACTTGCCTGTCGCTCTATCTTCCAAGCAGAAGACGGCATACGA
Bio-U-LT2	/5Biosg/TT/ideoxyU/ AGATCAATTAATACGATACCTGC
5Am12-fSBS3-ctgag	/5AmMC12/TTTTTTTTTT /ideoxyU//ideoxyU/CTACACTCTTTCCCTACACGACGCTCTTCCGATCTCTGAG
10T-3Am	TTTTTTTTTT/3AmMO/
5App-rSBS9-dd	/5rApp/AGATCGGAAGAGCACACGTCTGAACTCCAGTCA/3ddC/

Supplementary Table 2. Conversion of “hello world!” to template sequences.

Letter	Template ID	ASCII Decimal	Index (Binary)	Index (Ternary)	Data (Binary)	Data (Ternary)	Index+Data (Ternary)	Template sequence
**h**	H01	104	"0000"	"000"	"01101000"	"10212"	"00010212"	gAGACTGTG
**e**	H02	101	"0001"	"001"	"01100101"	"10202"	"00110202"	gAGTAGCTG
**l**	H03	108	"0010"	"002"	"01101100"	"11000"	"00211000"	gAGCGTCTC
**l**	H04	108	"0011"	"010"	"01101100"	"11000"	"01011000"	gACTACTCT
**o**	H05	111	"0100"	"011"	"01101111"	"11010"	"01111010"	gACGTAGTC
"space"	H06	32	"0101"	"012"	"00100000"	"01012"	"01201012"	gACAGTCGC
**w**	H07	119	"0110"	"020"	"01110111"	"11102"	"02011102"	gATCGTAGC
**o**	H08	111	"0111"	"021"	"01101111"	"11010"	"02111010"	gATACGACT
**r**	H09	114	"1000"	"022"	"01110010"	"11020"	"02211020"	gATGTAGCT
**l**	H10	108	"1001"	"100"	"01101100"	"11000"	"10011000"	gTCTACTCT
**d**	H11	100	"1010"	"101"	"01100100"	"10201"	"10110201"	gTCGTCAGT
**!**	H12	33	"1011"	"102"	"00100001"	"01020"	"10201020"	gTCAGTCAG

Supplementary Table 3. Extension lengths for perfectly synthesized strands of "hello world!".

Letter	Template ID	Template sequence	Median extension lengths	Average of median extension lengths
**h**	H01	gAGACTGTG	3,11,2,3,3,3,2,3	3.75
**e**	H02	gAGTAGCTG	4,11,2,3,4,2,2,2	3.75
**l**	H03	gAGCGTCTC	4,10,2,5,2,2,3,2	3.75
**l**	H04	gACTACTCT	4,4,3,3,3,4,2,4	3.375
**o**	H05	gACGTAGTC	5,4,9,1,2,4,1,2	3.5
"space"	H06	gACAGTCGC	5,4,11,6,1,2,5,2	4.5
**w**	H07	gATCGTAGC	4,3,2,9,1,3,4,2	3.5
**o**	H08	gATACGACT	4,3,4,3,8,2,3,3	3.75
**r**	H09	gATGTAGCT	4,4,5,2,3,5,2,3	3.5
**l**	H10	gTCTACTCT	2,3,4,3,3,4,2,4	3.125
**d**	H11	gTCGTCAGT	2,3,9,2,2,11,5,2	4.5
**!**	H12	gTCAGTCAG	2,3,12,7,2,3,9,5	5.375

Supplementary Table 4. Final concentration, in micromolar, of nucleoside triphosphates used for synthesizing H01-H12 template sequences.

From \ To	dATP	dCTP	dGTP	dTTP
**A-3'**	na	64	32	64
**C-3'**	256	na	128	512
**G-3'**	32	32	na	64
**T-3'**	64	32	32	na

Supplementary Table 5. Statistics from simulated real-time data reconstruction by nanopore sequencing.

Strand	Time required for robust data retrieval (hrs)	Fraction of total sequencing reads for robust data retrieval	Sequencing reads needed for robust data retrieval	Total sequencing reads
**H01**	24	0.75	511	679
**H02**	8	0.27	326	1210
**H03**	12	0.37	266	726
**H04**	6	0.15	145	1000
**H05**	6	0.16	213	1335
**H06**	12	0.43	248	581
**H07**	4	0.09	117	1360
**H08**	6	0.16	249	1519
**H09**	4	0.1	164	1580
**H10**	4	0.11	175	1647
**H11**	4	0.11	156	1375
**H12**	8	0.26	182	692

Supplementary Table 6. Commercial reagent prices and estimated reaction times for our enzymatic synthesis vs. phosphoramidite chemistry.

Comparison of V_f and V_d represent flowcell and droplet volume respectively (**Supplementary Note 6**). The two highlighted values on the bottom of the phosphoramidite chemistry price column are $\$_{fc}$ and $\$_{dc}$. The two highlighted values on the bottom of the enzymatic synthesis price column are $\$_{fe}$ and $\$_{de}$.

Phosphoramidite chemistry											
Reaction and Reagents				Price						Time	
	Reaction	Category	Reagent(s)	$ / ml	Commercial source	Volume units needed	Volume unit	Flowcell $/cycle.ml	Droplet $/cycle.ml	Flowcell s/cycle	Droplet s/cycle
Step 1	Detritylation	flowcell	3% trichloroacetic acid in dichloromethane	3.44E-02	Sigma L022500-01	1	Vf	3.44E-02		50	
Step 2	Wash	flowcell	Acetonitrile	5.75E-02	Sigma 271004-18L	2	Vf	1.15E-01		20	
Step 3	Flush	flowcell	Argon	1.13E-07	Med-Tech Gases 11J27	5	Vf	5.66E-07		10	
Step 4	Coupling	droplet	0.1M phosphoramidite monomer and 0.5M tetrazole in acetonitrile	1.99E+00	Sigma A111012-01 Sigma 88185-500ML	1	Vd		1.99E+00		60
Step 5	Wash	flowcell	Acetonitrile	5.75E-02	Sigma 271004-18L	2	Vf	1.15E-01		20	
Step 6	Flush	flowcell	Argon	1.13E-07	Med-Tech Gases 11J27	5	Vf	5.66E-07		10	
Step 7	Oxidation	flowcell	THF/Water/Pyridine/Iodine 90.5/9.1/0.4/0.4 (v/v/v/w)	5.34E-02	Sigma L060080-4X4L	1	Vf	5.34E-02		45	
Step 8	Wash	flowcell	Acetonitrile	5.75E-02	Sigma 271004-18L	2	Vf	1.15E-01		20	
Step 9	Flush	flowcell	Argon	1.13E-07	Med-Tech Gases 11J27	5	Vf	5.66E-07		10	
							**Total**	**4.33E-01**	**1.988478**	**185**	**60**

Enzymatic synthesis											
Reaction and Reagents				Price						Time	
	Reaction	Category	Reagent(s)	$ / ml	Commercial source	Volume units needed	Volume unit	Flowcell $/cycle.ml	Droplet $/cycle.ml	Flowcell s/cycle	Droplet s/cycle
Step 1	Extend	droplet	1U/ul TdT	6.05E+01	Enzymatics P7070L	1	Vd		6.05E+01		10
			1mU/ul apyrase	4.62E-01	Sigma A6535-2KU	1	Vd		4.62E-01		
			7mM Tris-acetate	1.44E-03	Sigma T1258-250G	1	Vd		1.44E-03		
			35mM potassium acetate	4.17E-03	Sigma P1190-1KG	1	Vd		4.17E-03		
			7mM magnesium acetate	2.33E-04	Sigma M5661-250G	1	Vd		2.33E-04		
			0.1% Triton X-100	3.00E-04	Sigma T8787-250ML	1	Vd		3.00E-04		
			10% PEG 8000	1.17E-02	Sigma 89510-1KG-F	1	Vd		1.17E-02		
			150uM dNTPs	3.30E-01	Sigma DNTP100A-1KT	1	Vd		3.30E-01		
Step 2	Wash	flowcell	10mM Tris	1.48E-04	Sigma T1503-1KG	2	Vf	2.96E-04		20	
			1mM EDTA	2.78E-05	Sigma 798681-1KG	2	Vf	5.56E-05			
Step 3	Flush	flowcell	Argon	1.13E-07	Med-Tech Gases 11J27	5	Vf	5.66E-07		10	
							**Total**	**3.52E-04**	**6.13E+01**	**30**	**10**

Supplementary Table 7. Parameters of DNA Storage Systems

Parameter	Description
K	Number of nucleotides per template sequence
Ω	Number of bits per template sequence (data and address bits)
μ	Number of address bits per template sequence ($\mu < \Omega$)
p_{del}	Probability of deletion (missing nucleotide)
p_{sub}	Probability of substitution (mismatch)
p_{ins}	Probability of insertion

Supplementary Table 8. Design Specifications of DNA Storage Systems

	Experiment	Experiment	Simulation	Simulation	Simulation
K-nt/sequence	8	16	38	74	152
Ω-bits/sequence	12	16	23	36	57
Total storage (Bits)	2^{μ} * (12 - μ)	2^{μ} * (16 - μ)	2^{μ} * (23 - μ)	2^{μ} * (36 - μ)	2^{μ} * (57 - μ)
Maximum theoretical storage (Bytes)	0.25 KB	4 KB	0.5 MB	4 GB	8 PB
Code design	Uncoded	(i) MAP estimation (ii) Consensus	(i) MAP estimation (ii) Consensus (iii) Sequence ECC	(i) MAP estimation (ii) Consensus (iii) Sequence ECC	(i) MAP estimation (ii) Consensus (iii) Sequence ECC
Diversity (strand[c] variants)	-	10	10	10	10
Error tolerance $p_{\text{ins}} + p_{\text{del}} + p_{\text{sub}}$	-	~ 20-30%	~ 20-30%	~ 20-30%	~ 20-30%

Supplementary Table 9. Modulation and demodulation: Interconversion of bits to nucleotides for “Eureka!” experiment

M1 Bits to Trits

Bits	Trits
000	00
001	01
011	02
010	12
110	10
111	11, 22
101	21
100	20

M2 Trits to Nucleotides

Prev	0	1	2
T	A	C	G
G	T	A	C
C	G	T	A
A	C	G	T

M3 Bits to Nucleotides

Prev	*Next*	0	1
A	A	T	C
T	A	C	G
C	A	G	T
G	A	T	C
A	T	G	C
T	T	A	C
C	T	A	G
G	T	A	C
A	C	G	T
T	C	A	G
C	C	A	T
G	C	A	T
A	G	T	C
T	G	A	C
C	G	A	T
G	G	C	A

Supplementary References

1. Chirpich, T. P. The effect of different buffers on terminal deoxynucleotidyl transferase activity. *Biochim. Biophys. Acta* **518**, 535–538 (1978).
2. Deibel, M. R., Jr & Coleman, M. S. Biochemical properties of purified human terminal deoxynucleotidyltransferase. *J. Biol. Chem.* **255**, 4206–4212 (1980).
3. Chang, L. M. & Bollum, F. J. Multiple roles of divalent cation in the terminal deoxynucleotidyltransferase reaction. *J. Biol. Chem.* **265**, 17436–17440 (1990).
4. Kato, K. I., Gonçalves, J. M., Houts, G. E. & Bollum, F. J. Deoxynucleotide-polymerizing enzymes of calf thymus gland. II. Properties of the terminal deoxynucleotidyltransferase. *J. Biol. Chem.* **242**, 2780–2789 (1967).
5. Grosse, F. & Manns, A. Terminal deoxyribonucleotidyl transferase (EC 2.7.7.31). *Methods Mol. Biol.* **16**, 95–105 (1993).
6. Yang, B., Gathy, K. N. & Coleman, M. S. Mutational analysis of residues in the nucleotide binding domain of human terminal deoxynucleotidyl transferase. *J. Biol. Chem.* **269**, 11859–11868 (1994).
7. Modak, M. J. Biochemistry of terminal deoxynucleotidyltransferase: mechanism of inhibition by adenosine 5'-triphosphate. *Biochemistry* **17**, 3116–3120 (1978).
8. Motea, E. A. & Berdis, A. J. Terminal deoxynucleotidyl transferase: the story of a misguided DNA polymerase. *Biochim. Biophys. Acta* **1804**, 1151–1166 (2010).
9. Delarue, M. *et al.* Crystal structures of a template-independent DNA polymerase: murine terminal deoxynucleotidyltransferase. *EMBO J.* **21**, 427–439 (2002).

10. Gouge, J., Rosario, S., Romain, F., Beguin, P. & Delarue, M. Structures of intermediates along the catalytic cycle of terminal deoxynucleotidyltransferase: dynamical aspects of the two-metal ion mechanism. *J. Mol. Biol.* **425**, 4334–4352 (2013).

11. Erlich, Y. & Zielinski, D. DNA Fountain enables a robust and efficient storage architecture. *Science* **355**, 950–954 (2017).

12. Blawat, M. *et al.* Forward Error Correction for DNA Data Storage. *Procedia Comput. Sci.* **80**, 1011–1022 (2016).

13. Organick, L. *et al.* Random access in large-scale DNA data storage. *Nat. Biotechnol.* **36**, 242–248 (2018).

14. Needleman, S. B. & Wunsch, C. D. A general method applicable to the search for similarities in the amino acid sequence of two proteins. *J. Mol. Biol.* **48**, 443–453 (1970).

15. Smith, T. F. & Waterman, M. S. Identification of common molecular subsequences. *J. Mol. Biol.* **147**, 195–197 (1981).

16. Notredame, C., Higgins, D. G. & Heringa, J. T-Coffee: A novel method for fast and accurate multiple sequence alignment. *J. Mol. Biol.* **302**, 205–217 (2000).

17. Wallace, I. M., O'Sullivan, O., Higgins, D. G. & Notredame, C. M-Coffee: Combining multiple sequence alignment methods with T-Coffee. *Nucleic Acids Res.* **34**, 1692–1699 (2006).

18. Russell, D. J. *Multiple Sequence Alignment Methods*. (Humana Press, 2016).

19. Batu, T., Kannan, S., Khanna, S., McGregor, A. & Reconstructing Strings From Random Traces. Reconstructing Strings From Random Traces. in *Proceedings of the Fifteenth Annual (ACM-SIAM) Symposium on Discrete Algorithms, (SODA), New Orleans, Louisiana, USA, January 11-14, 2004* 910–918

20. Davey, M. C. & Mackay, D. J. C. Reliable communication over channels with insertions, deletions, and substitutions. *IEEE Trans. Inf. Theory* **47**, 687–698 (2001).

21. Mitzenmacher, M. A survey of results for deletion channels and related synchronization channels. *Probab. Surv.* **6**, 1–33 (2009).

22. Kittler, J. Combining classifiers: A theoretical framework. *Pattern Anal. Appl.* **1**, 18–27 (1998).

23. Cover, T. M. & Thomas, J. A. *Elements of Information Theory*. (John Wiley & Sons, 2012).

24. Rashtchian, C. *et al.* Clustering Billions of Reads for DNA Data Storage. in *Advances in Neural Information Processing Systems 30* (eds. Guyon, I. et al.) 3360–3371 (Curran Associates, Inc., 2017).

25. Martin, M. Cutadapt removes adapter sequences from high-throughput sequencing reads. *EMBnet.journal* **17**, 10–12 (2011).

26. Hughes, T. R. *et al.* Expression profiling using microarrays fabricated by an ink-jet oligonucleotide synthesizer. *Nat. Biotechnol.* **19**, 342–347 (2001).

27. LeProust, E. M. *et al.* Synthesis of high-quality libraries of long (150mer) oligonucleotides by a novel depurination controlled process. *Nucleic Acids Res.* **38**, 2522–2540 (2010).

28. Blanchard, A. P., Kaiser, R. J. & Hood, L. E. High-density oligonucleotide arrays. *Biosensors and Bioelectronics* **11**, 687–690 (1996).

29. G4447A | Agilent. Available at: https://www.agilent.com/store/en_US/Prod-G4447A/G4447A. (Accessed: 5th April 2018)

30. FUJIFILM Dimatix collaborates with Agilent in developing inkjet technology for advanced life sciences applications | Press Center | Fujifilm USA. Available at:

http://www.fujifilmusa.com/press/news/display_news?newsID=880165. (Accessed: 4th April 2018)

31. Coughlin, T. The Costs Of Storage. *Forbes Magazine* Available at: https://www.forbes.com/sites/tomcoughlin/2016/07/24/the-costs-of-storage/. (Accessed 4th April 2018).

32. Zhang, Y., Zhu, B., Liu, Y. & Wittstock, G. Hydrodynamic dispensing and electrical manipulation of attolitre droplets. *Nat. Commun.* **7**, 12424 (2016).
